# Impact and Cost-effectiveness of Selective Human Papillomavirus Vaccination of Men Who Have Sex With Men

**DOI:** 10.1093/cid/ciw845

**Published:** 2016-12-23

**Authors:** Allen Lin, Koh J. Ong, Peter Hobbelen, Eleanor King, David Mesher, W. John Edmunds, Pam Sonnenberg, Richard Gilson, Irenjeet Bains, Yoon H. Choi, Clare Tanton, Kate Soldan, Mark Jit

**Affiliations:** 1 Department of Infectious Disease Epidemiology, London School of Hygiene and Tropical Medicine, London, United Kingdom;; 2 Department of Systems Biology, Harvard Medical School, Boston, Massachusetts;; 3 National Infections Service—Colindale, Public Health England, London, United Kingdom;; 4 Research Department of Infection and Population Health, University College London, London, United Kingdom

**Keywords:** human papillomavirus, vaccination, men who have sex with men.

## Abstract

**Background.:**

Men who have sex with men (MSM) have a high lifetime risk of anogenital warts and cancers related to infection with human papillomavirus (HPV). They also benefit less from herd protection than heterosexual males in settings with female-only HPV vaccination.

**Methods.:**

We evaluated the potential health impact and cost-effectiveness of offering vaccination to MSM who visit genitourinary medicine (GUM) clinics. We used a mathematical model of HPV 6/11/16/18 sexual transmission within an MSM population in England, parameterized with sexual behaviour, GUM attendance, HPV prevalence, HIV prevalence, warts, and cancer incidence data. Interventions considered were offering HPV vaccination to either HIV-positive MSM or MSM regardless of HIV status, for age bands 16–25, 16–30, 16–35, and 16–40 years.

**Results.:**

Substantial declines in anogenital warts and male HPV-related cancer incidence are projected to occur following an offer of vaccination to MSM. MSM not attending GUM clinics will partially benefit from herd protection. Offering vaccination to HIV-positive MSM up to age 40 is likely to be cost-effective if vaccine procurement and administration costs are below £96.50 a dose. At £48 a dose, offering vaccination to all MSM up to age 40 is likely to be cost-effective.

**Conclusions.:**

Quadrivalent HPV vaccination of MSM via GUM clinics is likely to be an effective and cost-effective way of reducing the burden of HPV-related disease in MSM.

## Summary

Offering human papillomavirus (HPV) vaccination to men who have sex with men up to age 40 years via genitourinary clinics will have a large impact on HPV-related diseases and is likely to be cost-effective.

Human papillomavirus (HPV) infection causes cervical, anal, penile, oropharyngeal, and oral cavity cancers as well as anogenital warts [1]. Most high-income countries vaccinate girls around 9–14 years old against HPV, but only a few countries (Austria, Australia, the United States, and several Canadian provinces) recommend extending vaccination to males (“gender-neutral vaccination”). When female vaccine coverage is high, heterosexual males are largely protected by herd protection, and hence vaccinating boys becomes less cost-effective [2]. However, men who have sex with men (MSM) benefit far less from this herd protection, despite bearing a disproportionately high burden of male HPV-related disease [3]. These predictions about herd protection have been confirmed empirically by post-female vaccination data from Australia showing large decreases in warts in heterosexual males but not in MSM [4].

Although most economic evaluations of gender-neutral vaccination have only considered heterosexual men, more recent evaluations have incorporated consideration of their impact on MSM [5–7]. Even these evaluations find that extending a female vaccination program to males would not be cost-effective in settings with high female vaccine coverage unless female vaccine coverage and/or vaccine prices are sufficiently low [7]. However, a selective vaccination program for MSM may address the inequity in disease burden and vaccine provision, while still potentially being cost-effective. MSM who are unvaccinated prior to same-sex debut may still benefit through herd protection from vaccination of their male partners. Such a strategy must include a mechanism for identifying and reaching MSM (ideally soon after same-sex debut), such as delivery through a clinical setting where MSM self-identify to health professionals.

In England, HPV vaccination has been offered to girls aged 12–13 since September 2008, with uptake exceeding 80%. At these coverage levels, adding boys to a girls-only HPV vaccination program is unlikely to be cost-effective [8]. Genitourinary medicine (GUM) clinics have historically provided a specialized service to MSM and could provide an avenue for a selective program. However, there is little evidence about the effectiveness and cost-effectiveness of such an approach, because not all MSM attend GUM clinics, and those that do may attend after being exposed to HPV.

To address this gap, we have evaluated the potential health impact and cost-effectiveness of offering vaccination to MSM via GUM clinics, beginning in year 2016, using a model of HPV 6/11/16/18 sexual transmission within an MSM population, and data from these clinics in England.

## METHODS

### Overview

**Figure 1.**
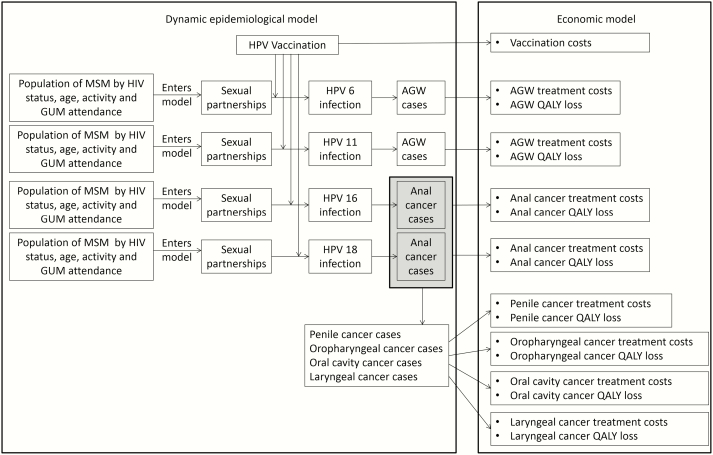
Model flow diagram showing the four dynamic models of HPV 6/11/16/18 infection in MSM, together with economic models of the cost and quality of life implications of their outcomes. Abbreviations: AGW, anogenital warts; HIV, human immunodeficiency virus; HPV, human papillomavirus; GUM, genitourinary clinic; MSM, men who have sex with men; QALY, quality-adjusted life year.

Our analysis consisted of interlinked models of: (i) same-sex HPV 6/11/16/18 transmission within an MSM population as well as the impact of vaccination, (ii) natural history of HPV infection and disease (anogenital warts, anal, penile, oropharyngeal, oral cavity, and laryngeal cancer), and (iii) costs and quality-adjusted life year (QALY) implications of disease outcomes (see Figure 1). A brief model description is given below and key parameters used are shown in Table 1. Full details including model flow diagrams and equations are in the Supplementary Material.

**Table 1.** Summary of Demographic, Epidemiological, Sexual Behavior, and Clinic Attendance Parameters Used in the Models

**Parameter**	**Depends on**	**Value**			**Source**
Demographics parameters					
No. of 10-year-old boys in England		292700			[38]
% of male population that is MSM	Age	Peaks at 3.47 at age 35			[39]
Monthly natural mortality rate without HIV	Age	1.6 × 10^–5^ to 5.4 × 10^–3^			[38]
Mortality rate ratio in HIV-positives and HIV-negatives		2.18			[11]
% of HIV-positive MSM undiagnosed		22.65			[40]
HIV prevalence in MSM	Age	Peaks at 12.7% at age 46			[15, 40]
		Low activity	Mid activity	High activity	
Monthly HIV force of infection	Activity group, age	Max: 5.3 × 10^–4^	1.35 RR vs low	2.37 RR vs low	[18, 41]
Median age of sexual debut	Activity group	17	16	15	[39]
Epidemiological parameters					
% of anogenital warts due to HPV-6/11		90			[42]
% of HPV-6/11-related anogenital warts due to HPV-11		10, 15, or 25			[42–44]
% of anal cancers caused by HPV-16/18		69.4–73.8			[45]
% of HPV-16/18-related cancers due to HPV-18		1.3–4.3			[45]
Partner who governs the probability of HPV transmission per partnership		Either low- or high-activity partner			
HPV vaccine efficacy against HPV-6/11		77.6% (95% CI: 61.4–87.0)			[22]
HPV vaccine efficacy against HPV-16/18		63.7% (95% CI: 44.5–76.2)			[22]
Duration of vaccine-induced immunity		Lifelong or 20 years			
		HIV-negative	HIV-positive		
Duration of HPV natural immunity	HIV	Lifelong, 20, 10, 3, or 0 yrs	Lifelong, 20, 10, 3, or 0 yrs		
HPV clearance rate (cleared episodes/1000 person-months)	HIV	50, 80, 110, 140, or 170	8, 12, 16, 20, or 24		[46, 47]
Percentage of HPV-6/11-infections causing anogenital warts	HIV	10, 20, or 30	10, 20, or 30		[14]
No. of first warts diagnoses at each age	HIV, age	Max: 152	Max: 16		[18]
Prevalence of HPV 16 (ages 18–40)	HIV, age	Mean: 11%	Mean: 33%		[15]
Prevalence of HPV 18 (ages 18–40)	HIV, age	Mean: 4%	Mean: 8%		[15]
Anal cancer incidence (per 100000 py)	HIV, age	Max: 18.5	Max: 282		[3, 48]
Oropharyngeal cancer incidence (per 100000 py)	HIV, age	Max: 6.7	Max: 12.7		[17, 48]
Penile cancer incidence (per 100000 py)	HIV, age	Max: 2.2	Max: 6.3		[17, 48]
Oral cavity cancer incidence (per 100000 py)	HIV, age	Max: 11.2	Max: 21.9		[17, 48]
Laryngeal cancer incidence (per 100000 py)	HIV, age	Max: 9.3	Max: 24.0		[17, 48]
Anal cancer survival	Age	70–91% after 1 year			[34, 49, 50]
Oropharyngeal cancer survival	Age	57–88% after 1 year			[34, 51, 52]
Penile cancer survival	Age	77–94% after 1 year			[53]
Oral cavity cancer survival	Age	64–84% after 1 year			[51]
Laryngeal cancer survival	Age	75–90% after 1 year			[54]
Sexual behaviour parameters					
Age group assortativeness in MSM-MSM partnerships		47%			[39]
Age group assortativeness in MSM-female partnerships		40%			[39]
Activity group assortativeness		0.1, 0.5, or 0.9			
HIV assortativeness		0.1, 0.5, or 0.9			
		Low activity	Mid activity	High activity	
% of MSM population in each activity group		80	15	5	[39]
Same-sex partner change rate (per 3 months)	Activity group, age	Max: 0.6	Max: 4.4	Max: 17.1	[39]
Female partner change rates (per year)	Activity group, age	Max: 0.5	Max: 0.06	Max: 0.05	[39]
Clinic attendance parameters					
		Low activity	Mid activity	High activity	
% MSM attending GUM clinics	Activity group	48	70	79	[10, 18]
Probability of clinic debut	Age	50% debut by age 21			[18]
		HIV-negative	HIV-positive		
Monthly clinic attendance rate in attenders	HIV status, age	Max: 10%	Max: 15%		[18]
No. of clinic visits per episode of anogenital warts	HIV status	1.16	1.20		[18]
		Dose 1	Dose 2	Dose 3	
Vaccine uptake and completion	Dose	89%	69%	49%	[15, 20]

Abbreviations: CI, confidence interval; GUM, genitourinary clinic; HIV, human immunodeficiency virus; HPV, human papillomavirus; MSM, men who have sex with men; py, person-years; RR, relative risk.

### Modeled Population Context

The third National Survey of Sexual Attitudes and Lifestyles (Natsal-3) [9] suggests that 33% of MSM attended GUM clinics in the past year and 52% had ever attended [10]. MSM attending GUM clinics are at higher risk of human immunodeficiency virus (HIV) infection and sexually transmitted infection (STI) [11, 12]. Antiretroviral treatment coverage is high (90%) for the 38432 MSM accessing HIV care [13].

### Transmission

Similar to our previous model of heterosexual HPV 6/11/16/18 transmission [14], we use a SIRS (susceptible-infected-recovered-susceptible) structure, except now with same-sex parameters to exclusively model partnerships between MSM aged 10–74. These MSM are further stratified based on their age (in months), sexual activity-based risk group (based on partner change rates), HIV status, and whether or not they attend GUM clinics. At each age, they are either not yet same-sex active, same-sex active (and hence susceptible to infection), infected by a particular HPV type or having cleared an infection (and obtain natural immunity). The proportion of men who become same-sex active at each year of age was estimated from Natsal-3 [9], assuming all MSM reach same-sex debut before age 35, capturing 95% of Natsal-3 responses. Age- and risk group-specific same-sex and female partner change rates, and mixing between age groups are informed by the same data. Partnerships include oral-genital, anal-genital, and other genital contact, without distinguishing between transmission routes. Projected female HPV prevalence declines, and estimated transmission probabilities from previous modeling [14] were used to calculate infection risk from sex with women amongst MSM. Individuals who clear infections can receive short-term type-specific immunity that can subsequently wane.

### Disease Natural History

Disease outcomes modeled were anogenital warts (for HPV 6/11) and all male cancers (for HPV 16/18) classified by the International Agency for Research on Cancer (IARC) as having evidence of causation by HPV 16, that is, cancers of the anus, penis, oropharynx, and oral cavity [1]. Laryngeal cancers (where “epidemiological evidence is not conclusive to confirm the role of HPV 16 or 18”) were included in sensitivity analysis.

For warts, we adapted a previous model [14], in which 10%–30% of newly infected individuals develop warts and seek GUM clinic treatment. For anal cancer, we developed a de novo model of HPV 16/18 infection progressing to low- and high-grade anal intraepithelial neoplasia, and finally to cancer. Rates governing transitions between different disease stages were estimated from the literature and by fitting to age-standardized anal cancer incidence in English MSM, and age-specific anal HPV prevalence in 511 MSM attending a London-based GUM clinic (both stratified by HIV status) [15]. Non-anal cancers were dealt with in a simpler way due to limited data on their natural history. For each year following the initiation of MSM vaccination, the proportionate reduction in anal cancer incidence by age, HIV status, and HPV type from the pre-vaccination equilibrium predicted by the model was applied to the corresponding incidence of the other cancers. Age-specific incidence of HPV-related cancers was obtained from the Office for National Statistics. Risk of progression to non-anal cancers was assumed to be similar for both HIV-negative MSM and heterosexual men [16] but higher in HIV-positive MSM [17].

### Vaccination

We considered a strategy of offering quadrivalent HPV vaccination to either HIV-positive MSM or MSM regardless of HIV status, and to either 16–25, 16–30, 16–35, or 16–40 year olds. Offering vaccination outside this age range was not modelled because of limited GUM clinic attendance data, and in the case of under 16s, confidentiality constraints.

When vaccination is initiated, all MSM of the eligible age range and HIV status attending GUM clinics are offered vaccination. At subsequent time steps, vaccination is offered only to MSM attending GUM clinics for the first time since the selective vaccination program was initiated. GUM attendance rates were based on 2009–2012 clinic returns [18], stratified by known HIV-positive status [19]. Dose completion for the 3-dose schedule was based on MSM hepatitis B vaccination completion rate reported by a London GUM clinic [20]; surveys of GUM-attending MSM [15] and sexual health professionals [21] suggest similarly high acceptability for HPV vaccines.

Quadrivalent vaccine efficacy against persistent infection from the naive-to-relevant type cohort in trials in males was used [22]. We assumed that vaccinees who fail to complete the schedule, receive all doses but fail to be immunized or lose vaccine protection are not offered revaccination. In the base case, lifelong vaccine protection is assumed based on lack of observed waning in eight years of follow-up for quadrivalent vaccine trials in 9–15 year-old boys and girls [23]. Vaccination is assumed to have no effect on clearance or disease progression of HPV infection acquired prior to receiving the first of 3 doses [24]. However, MSM who clear a prevalent vaccine-type HPV infection subsequent to vaccination are assumed to be protected from later infections of the same type.

### Economic Analysis

We estimated changes in costs (due to both vaccination and health care for HPV-related diseases) and quality adjusted life years (QALYs) following vaccination. The economic evaluation followed the reference case of the National Institute for Health and Care Excellence [25], as interpreted by the Joint Committee on Vaccination and Immunisation (JCVI)’s Working Group on Uncertainty in Vaccine Evaluation and Procurement [26]. In particular, a healthcare provider perspective was adopted. and health outcomes were measured in QALYs. Costs and benefits were discounted to 3.5% in the base case and to 1.5% in a sensitivity analysis. Costs were inflated to 2013/14 prices using the Hospital and Community Health Services index [27]. A time horizon of 100 years was used in line with previous analyses of HPV vaccination [8]. We used a cost-effectiveness threshold of £20 000/QALY. Vaccine procurement and administration was assumed to cost either £96.50/dose or £48/dose. We also calculated the threshold price per dose for vaccination to be cost-effective, as the net (discounted) monetary benefit of vaccination (converting QALYs using a conversion factor of £20 000/QALY) divided by the number of doses delivered.

### Uncertainty Analysis

We constructed 5000 meta-scenarios by altering assumptions governing sexual partnership formation, HPV epidemiology and disease natural history for each HPV type. Each meta-scenario was fitted by varying transmission probability per partnership to minimize the sum of squared residuals between data and model outcomes by age and HIV status (warts incidence for HPV 6/11 and anal HPV prevalence for HPV 16/18). The 1000 best-fitting meta-scenarios were paired with 1000 sets of parameters drawn using Latin hypercube sampling from the distribution of cost and QALY consequences of HPV-related disease.

In addition to probabilistic sensitivity analyses, we also considered the following alternative scenarios: (i) Low/high vaccine efficacy, based on the lower/upper limits of the confidence interval around efficacy [22], (ii) Vaccine protection wanes completely after a fixed duration of 20 years, (iii) Vaccines protect against laryngeal cancer, (iv) Bivalent instead of quadrivalent vaccination, (v) 1.5% instead of 3.5% discount rate, (vi) 100% 3-dose completion rate, (vii) No herd protection, so vaccines only reduce the risk of infection in vaccinees.

## RESULTS

**Figure 2.**
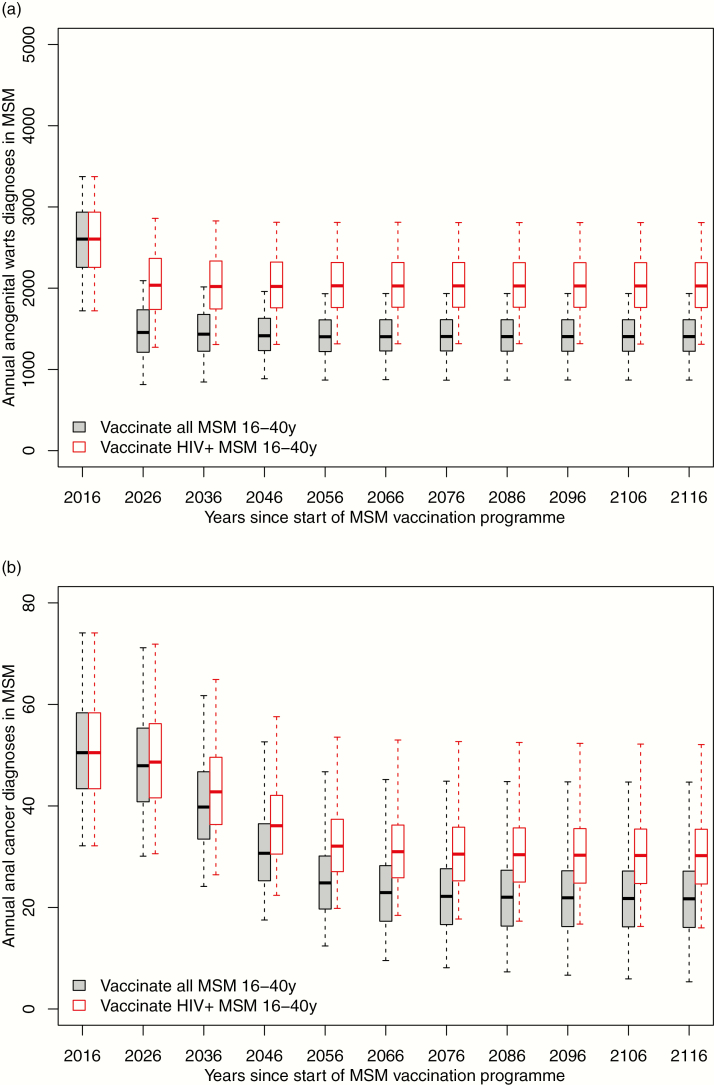
Proportionate reduction over time in annual cases of (a) anogenital warts and (b) anal cancer following quadrivalent human papilloma virus vaccination of MSM attending genitourinary medicine clinics aged 16–40. Boxes show interquartile range (with the notch as the median), while whiskers indicate the entire range across 1000 meta-scenarios. Abbreviations: HIV, human immunodeficiency virus; MSM, men who have sex with men.

Results show rapid declines in warts incidence by 35% (interquartile range 32%–39%) within 5 years of initiating vaccination for 16–40 year old MSM GUM attendees and 15% (12%-18%) if only HIV-positive 16–40 year old MSM are vaccinated (Figure 2). Herd protection is likely to be marked since MSM over a large age range (16–40 years) will receive vaccination in the first year. Declines in cancer take longer, due to the time lag between infection and cancer manifestation. Large cancer incidence reductions (eg, 55% (50%–64%) reduction over 100 years for anal cancer) are eventually expected if all clinic attending MSM aged 16–40 years are offered vaccination. This reduction is smaller (e.g., 40% (36%–45%) over 100 years for anal cancer) if only HIV-positive 16–40 year old MSM are vaccinated (section A11 of Supplementary Material).

With the quadrivalent vaccine costing £96.50/dose, the best option with an incremental cost-effectiveness ratio (ICER) below £20 000/QALY gained would be to vaccinate all HIV-positive MSM 16–40 years (Table 2). If the vaccine costs only £48/dose, vaccination becomes cost saving for this cohort, and could be extended to all MSM 16–40 years.

**Table 2.** Incremental Costs, QALYs Gained and Cost per QALY Gained over 100 years for the Different Vaccination Options

**Vaccination option**	**Vaccine doses**		**Incremental costs (£m**)		**Incremental QALYs gained**			**Incremental cost (£) per QALY gained**	
	**Undiscounted**	**Discounted**	**£96.50/ dose**	**£48/** **dose**	**Due to warts**	**Due to cancers**	**Total**	**£96.50/** **dose**	**£48/** **dose**
No vaccination	0	0							
HIV + 16–25	65 288	19100	−0.39	−1.32 ^a^	172	289	461	Cost saving^a^	Cost saving^a^
HIV + 16–30	126158	18700	0.21	−0.69 ^a^	96	219	315	682	Cost saving
HIV + 16–35	183605	18800	0.58	−0.34 ^a^	61	172	233	2470	Cost saving
HIV + 16–40	234452	18200	0.83	−0.05	37	124	161	5160	Cost saving
All 16–25	941495	207000	19.3	9.23	194	47	241	80100 ^b^	38300 ^b^
All 16–30	1172038	295000	25.8	11.5	323	312	634	40600 ^b^	18100 ^b^
All 16–35	1269048	348000	29.7	12.9	384	477	861	34500 ^b^	14900 ^b^
All 16–40	1335684	395000	33.4	14.3	423	596	1020	32800	14000

Each strategy is compared with the previous most effective nondominated strategy. Number of doses, costs and QALYs are discounted at 3.5% per annum.

Abbreviations: HIV, human immunodeficiency virus; QALY, quality-adjusted life year.

^a^Strongly dominated (costs more and is less effective than another strategy).

^b^Weakly dominated (costs more and is less effective than a combination of other strategies).

Multivariate uncertainty analysis suggests this conclusion is robust (section A13 of Supplementary Material). JCVI considers vaccination cost-effective if the most plausible ICER falls below £20 000/QALY gained, and there is no more than a 10% probability that the ICER exceeds £30 000/QALY gained [26]. At a vaccine cost of £48/dose, in our analysis vaccinating all MSM is more cost-effective than the next best alternative (vaccinating all HIV-positive MSM) in 85.4% of scenarios when the threshold is £20 000/QALY gained, and 99.3% of scenarios when the threshold is £30 000/QALY gained. Hence at £48/dose vaccinating 16–40 year old MSM would satisfy the JCVI conditions. One-way sensitivity analyses suggest that cost-effectiveness is most sensitive to uncertainty around vaccine costs, the disutility around warts episodes, as well as the duration and cost of anal cancer treatment (see details in section A12 of the Supplementary Material).

Table 3 shows how the cost-effectiveness of vaccinating all 16–40 year old MSM, compared with vaccinating the next most expensive non-dominated option, that is, HIV-positive 16–40 year old MSM, changes with alternative scenarios about HPV epidemiology and vaccine action. Threshold price per vaccine dose for such an extension of vaccination to be cost-effective is £63 in the base case, ranging from £33 (if vaccine protection lasts only 20 years) to £97 (if discounting at 1.5%).

**Table 3.** Incremental Cost-effectiveness Ratio and Threshold Vaccine Cost per Dose (for Procurement and Administration) of Vaccinating 16–40 Year old MSM (Compared to the best Alternative Scenario of Vaccinating HIV-positive 16–40 year old MSM) Under Different Assumptions

**Scenario**	**Threshold cost per dose (£**)	**Cost (£) per QALY gained**	
		**£96.50/dose**	**£48/dose**
Base case	63	32800	14000
1.5% discounting	97	19800	7800
Protection against laryngeal cancers	68	30500	12800
Vaccine duration of 20 years	33	66900	31000
Low (61.4%, 44.5%) vaccine efficacy	50	43000	19100
High (87.0%, 76.2%) vaccine efficacy	71	28900	12100
No herd effects	35	62000	28600
100% dose completion	73	27800	11500

Abbreviations: HIV, human immunodeficiency virus; MSM, men who have sex with men; QALY, quality-adjusted life year.

Using the bivalent vaccine instead of the quadrivalent vaccine is only likely to be the most cost-effective option if the bivalent vaccine is £41 or cheaper per dose than the quadrivalent vaccine.

## DISCUSSION

Quadrivalent HPV vaccination of 16–40 year old MSM attending GUM clinics is cost-effective if the vaccine can be procured and delivered at no more than £63/dose in the base case (£33–£97 across scenario sensitivity analyses). Although HPV vaccine tender prices in England are unknown, equivalent prices in high-income countries submitting data to the World Health Organization range from £20.90 to £48.00 [28], while delivery costs of £10/dose may be reasonable [29]. Even with vaccine costs at £96.50/dose, a more limited program offering vaccination to HIV-positive 16–40 year old MSM would be cost-effective. A nonavalent vaccine at the same price is likely to have a similar cost-effectiveness profile because almost all male HPV-related cancers are caused by HPV 16/18. However, a bivalent vaccine is unlikely to be cost-effective in a selective MSM program given that it needs to be at least £41/dose cheaper to procure and deliver than the quadrivalent vaccine.

Besides vaccine costs, results are sensitive to uncertainty in the disutility caused by warts or anal cancer, and the cost of treating anal cancer. Warts’ disutility is especially influential and is driven particularly by variability not just in the measured quality of life of someone with warts but also the duration of time spent with warts [30].

The cost-effectiveness of MSM vaccination may be even better than reported due to additional benefits of vaccination not fully captured. First, although we assumed that HIV increases the rate of HPV-related disease progression, we assumed for computational simplicity that HPV has no effect on HIV acquisition, despite some evidence to the contrary [31]. Second, our model was fitted to recent cross-sectional data on sexual behavior, GUM attendance among MSM, HIV prevalence, and cancer incidence. In the future, these data may change, although the direction of change is difficult to predict. However, both anal [32] and oropharyngeal [33] cancer incidence has been increasing, and the increase may be particularly pronounced among HIV-positive MSM due to improved survival in the era of antiretroviral therapy. Third, we only modeled disease occurring in the ages 10–74 years. An estimated 24%, 30%, and 8% of anal, penile, and oropharyngeal cancers, respectively, in 2008–12 in England [34] occurred in men aged 75 and older. However, the importance of these cancers to the cost-effectiveness of vaccination is reduced by discounting because vaccination occurs much earlier and because these men have lower life expectancies. Fourth, it is possible that offering HPV vaccination at GUM clinics may increase attendance rates among young MSM. A survey among 16–20 year old MSM in Australia found that 86% would be willing to disclose their sexual orientation to a healthcare provider in order to receive HPV vaccination if it were free of charge [35]. Such an effect would increase vaccine uptake as well as potentially uptake of other sexual health and health promotion services that may reduce the incidence of other sexually transmitted diseases, albeit at increased costs.

Our modelling has limitations because we model compartments of the MSM population rather than discrete individuals. In particular, we divide the population into 3 sexual activity tiers, within which individuals have the same number of partners. Thus, we do not separately model those rare individuals with many partners, even compared to the 5% most same-sex active individuals. In addition, we do not vary all parameters in the model, such as those obtained from Natsal-3 or GUMCAD. However, by widely varying other correlated epidemiological and vaccination uptake parameters, we likely capture the uncertainty in outcomes. Furthermore, the data requirements of an individual-based model may not be justified given the sparsity of data on MSM. Lastly, we do not model separate disease risks for diagnosed and undiagnosed HIV-positive MSM.

Only 2 cost-effectiveness analyses of selective MSM HPV vaccination programs have been previously conducted, both in the United States [36, 37]. Both used static models and hence did not consider potential herd protection from vaccinating only a proportion of MSM. One study [37] only explored a limited strategy of vaccination as adjunct prevention in HIV-negative MSM following treatment for high-grade anal neoplasia and concluded that it may be cost-effective. A second study assumed that all MSM would be vaccinated at a certain age and found that at a composite vaccination cost of US$500 (about £100/dose in 2014 GBP) per vaccinated individual, vaccinating MSM up to age 26 was cost-effective at a threshold of US$50000 (£30000 in 2014 GBP) per QALY gained. To our knowledge, our study is the first cost-effectiveness evaluation of selective HPV vaccination for MSM explicitly considering a delivery pathway to reach MSM. It is also the first to use a transmission model, which was critical to identifying the most cost-effective strategy. By doing so, we show that such a strategy may bring substantial population-level benefits even though not all MSM attend GUM clinics.

Our work suggests that MSM HPV vaccination can be delivered in a feasible and cost-effective way in settings where MSM regularly attend specialist sexual health services. Although our analysis considered GUM clinics only, the results likely apply to other sexual health service providers able to deliver vaccines attended by MSM with a similar HPV infection risk profile. Although we only considered vaccination between the ages of 16 and 40 due to data limitations, vaccinating younger MSM is highly likely to also be cost-effective, and our analysis does not preclude that vaccinating beyond age 40 could also be cost-effective.

Although GUM clinic-based HPV vaccination for MSM was found to be cost-effective with large impact on disease incidence, the largest reductions in HPV-related disease will only occur through universal vaccination of 12–13 year-old boys, because many MSM initiate same-sex activity and hence are at risk of HPV infection before attending such clinics. However, introducing gender-neutral vaccination does not preclude offering vaccination to MSM up to a higher age, particularly because many MSM were not born in England and may be missed by an adolescent program.

## Supplementary Material

Supplementary Data

## Notes

***Author contributions.*** A.L. and K.J.O. contributed equally to this work. A.L., K.J.O., M.J., K.S., P.H., E.K., and D.M. conceptualized the model and interpreted the results. AL coded and parameterized the model with input from K.J.O., P.H., Y.H.C., M.J., and K.S. D.M., E.K., K.S., W.J.E., P.S., R.G., I.B., and C.T. provided and analyzed key data sources for the model. K.J.O., A.L., and M.J. conducted the economic evaluation. A.L., K.J.O., and M.J. wrote the manuscript with input from all other authors. All authors approved the final version of the manuscript. M.J. is the guarantor.

***Acknowledgments.*** We thank Mary Ramsay, Vanessa Saliba, Andrew Earnshaw, Jonathan Crofts, and Cath Mercer for helpful comments. We also thank Mona Jeffreys and Rouran Li for sharing unpublished data from their studies. We thank all clinics that report data to the Genitourinary Medicine Clinic Activity Dataset (GUMCAD), Survey of Prevalent HIV Infections Diagnosed (SOPHID), and HIV and AIDS Reporting System (HARS), as well as the GUMCAD/SOPHID/HARS team at Public Health England for collating and validating the data.

***Disclaimer.*** The views expressed are those of the authors and not necessarily those of the National Health System, the National Institute for Health Research (NIHR), the Department of Health or Public Health England. The funders had no role in study design; in the collection, analysis, and interpretation of data; in the writing of the report; and in the decision to submit the article for publication.

***Funding.*** This work was partially supported by the National Institute for Health Research Health Protection Research Unit (NIHR HPRU) in Immunisation at the London School of Hygiene and Tropical Medicine in partnership with Public Health England (grant IS_HPU_1112_100). A.L. was supported by a British Marshall Scholarship.

Natsal-3 is a collaboration between University College London, the London School of Hygiene and Tropical Medicine, NatCen social research, Public Health England (formerly the Health Protection Agency), and the University of Manchester. The study was supported by grants from the Medical Research Council [G0701757]; and the Wellcome Trust [084840]; with contributions from the Economic and Social Research Council and Department of Health. We thank the study participants and the team of interviewers from NatCen social research who carried out the fieldwork. The Natsal study was approved by the Oxford Research Ethics Committee A [Ref [10]:/H0604/27].

***Potential conflicts of interests.***  W.J.E.’s partner works for GlaxoSmithKline; all other authors have no other relationships or activities that could appear to have influenced the submitted work. All authors have submitted the ICMJE Form for Disclosure of Potential Conflicts of Interest. Conflicts that the editors consider relevant to the content of the manuscript have been disclosed.

***Ethics.*** Ethics approval was not required for this study. The Natsal study was approved by the Oxford Research Ethics Committee A [Ref [9]:/H0604/27].
