# Supplementary material for: Impact and Cost-effectiveness of Selective Human Papillomavirus Vaccination of Men Who Have Sex With Men
Source: Clin Infect Dis. 2016 Dec 23;64(5):580–8. doi: 10.1093/cid/ciw845 (PMC5404831; doi:10.1093/cid/ciw845)
Supplement: Supplementary Data [file ciw845_Supplementary_Data.zip › HPV_MSM_CEA_CIDMS82454_Revision_2_Appendix.docx]

**Technical Appendix for “Human papillomavirus vaccination of men who have sex with men via genitourinary medicine clinics: a transmission model-based cost-effectiveness analysis”**

This appendix gives further details about the epidemiological and economic data sources and analysis used to parameterise the model, as well as the detailed model structure. Further details about the model scenarios that best fit data are also provided.

Table of Contents

[A1. Detailed model description 3](#_Toc468110438)

[A2. Epidemiological and demographic parameters 5](#_Toc468110439)

[A2.1. Size of the men who have sex with men (MSM) population 5](#_Toc468110440)

[A2.2. Age at same-sex debut 6](#_Toc468110441)

[A2.3. Same-sex partner change rates 8](#_Toc468110442)

[A2.4. Population mixing 11](#_Toc468110443)

[A2.5. Female-to-male HPV transmission 13](#_Toc468110444)

[A2.6. GUM attendance and anogenital warts diagnoses 16](#_Toc468110445)

[A2.7. HIV status 18](#_Toc468110446)

[A2.8. Vaccine efficacy 20](#_Toc468110447)

[A2.9. HPV-related cancer incidence 21](#_Toc468110448)

[A2.10. HPV attributable fraction in cancer 22](#_Toc468110449)

[A3. Scenario selection and calibration 23](#_Toc468110450)

[A3.1. Model calibration 23](#_Toc468110451)

[A3.2. HPV prevalence 23](#_Toc468110452)

[A3.3. Scenarios 24](#_Toc468110453)

[A3.4. HPV transmission 26](#_Toc468110454)

[A3.5. Natural history parameters for anal cancer 27](#_Toc468110455)

[A4. Parameters affecting disease burden and costs 32](#_Toc468110456)

[A4.1. Quality of life 32](#_Toc468110457)

[A4.1.1. Anogenital warts 32](#_Toc468110458)

[A4.1.2. Cancer: combining utility values during treatment, recovery, and post-cancer survival 32](#_Toc468110459)

[A4.2. Survival estimates and utility values post-cancer 32](#_Toc468110460)

[A4.2.1. Anal cancer survival rates 33](#_Toc468110461)

[A.4.2.2. Oropharyngeal cancer survival rates 34](#_Toc468110462)

[A4.2.3. Penile cancer survival rates 35](#_Toc468110463)

[A4.2.4. Oral cavity and laryngeal cancer survival rates 36](#_Toc468110464)

[A4.3. Utility values for the post-cancer period 37](#_Toc468110465)

[A4.4. Costs 38](#_Toc468110466)

[A.4.4.1. Anogenital wart (AGW) treatment costs 38](#_Toc468110467)

[A.4.4.2. Cancer treatment costs 38](#_Toc468110468)

[A5. Summary tables of parameters 39](#_Toc468110469)

[A6. Model equations 41](#_Toc468110470)

[A7. Computer simulations 44](#_Toc468110471)

[A9. Meta-scenario fits and transmission probabilities 45](#_Toc468110472)

[A8. Best fitting meta-scenarios: assumptions selected 50](#_Toc468110473)

[A9. Sample model outcomes 55](#_Toc468110474)

[A10. Model outcomes by HPV type 60](#_Toc468110475)

[A11. Model outcomes summarized 69](#_Toc468110476)

[A12. One-way sensitivity analyses 70](#_Toc468110477)

[A13. Cost-effectiveness acceptability curves 73](#_Toc468110478)

[References 76](#_Toc468110479)

# A1. Detailed model description

We developed a set of population-based, discrete-time compartmental transmission dynamic models with monthly time steps. The models were stratified by HPV type (6, 11, 16 and 18), monthly age (i), HIV status (h), sexual activity-based risk group (l), GUM clinic attendance behaviour (c), and vaccination status (v). The models for each HPV type (6/11/16/18) follow the same basic SIRS structure, in which individuals can be susceptible to a type-specific HPV infection (S), infected (I), recovered and temporarily immune (R), and back to susceptible. There is an additional non-sexually active state (NA) at the beginning. Individuals enter this state in the model from the age of 10 and subsequently become sexually active and hence susceptible at their sexual debut with probability *a*. Susceptible individuals are infected at a force of HPV infection of λ_HPV_, subsequently lose their infection to become recovered at rate (per monthly time step) *r*, then return to being susceptible again at rate μ.

Unvaccinated individuals (v=0) may be vaccinated at a probability *v*, a proportion γ of whom move to the successfully immunised state (v=1). The remaining unsuccessfully immunised vaccinees move to the vaccine failure state (v=2). In addition, individuals who experience waning of vaccine-induced immunity also move to the vaccine failure state at probability *w*, dependent on the duration of vaccine-induced immunity. Any sexually active MSM is only offered vaccination (with three doses, in the base case) once in his lifetime, regardless of his subsequent state of vaccine protection.

In addition, individuals exit the models through natural mortality, and can become HIV positive at age- and activity group-dependent rates.

**Figure**. Basic structure of the SIRS model for each HPV type.


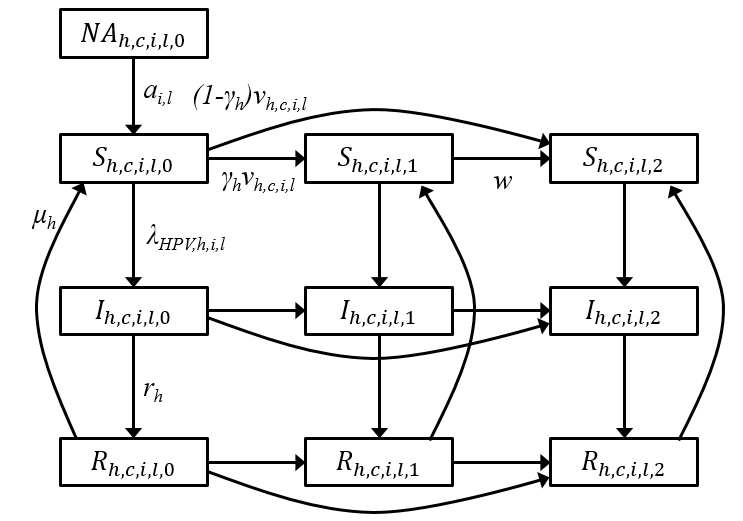


HPV-6 and HPV-11 models followed the same structure, which, in addition to the core model described above, account for the fact that a proportion of newly infected MSM (β) develop anogenital warts and as a result start attending GUM clinics.

**Figure.** Basic structure of the SIRS model for HPV-6 and 11.


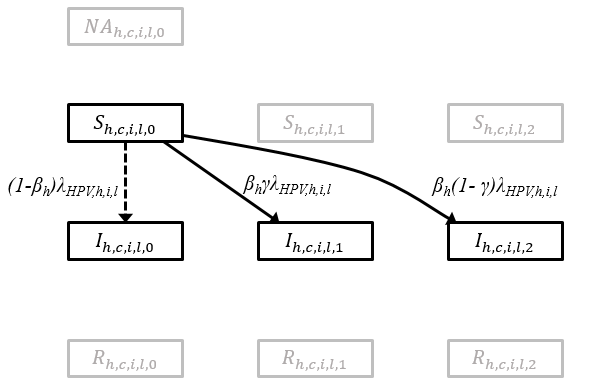


HPV-16 and HPV-18 models also follow the same structure, which, in addition to the core model described above, account for the fact that infected individuals may transition from HPV-16/18 infection to low-grade anal intraepithelial neoplasia (LGAIN) (probability σ), from LGAIN to high-grade anal intraepithelial neoplasia (HGAIN) (probability χ) and from HGAIN to anal cancer (probability ω) as shown in the figure below. The infection may clear naturally. This moves individuals from LGAIN and HGAIN to the immune state at probabilities of ρ and ϕ, respectively.

Once someone acquires cancer he is assumed to stay in that state permanently. Quality of life and costs associated with cancer mortality, survival and recurrence and modelled in the interlinked economic model.

**Figure.** Basic structure of the SIRS model for HPV-16 and 18.


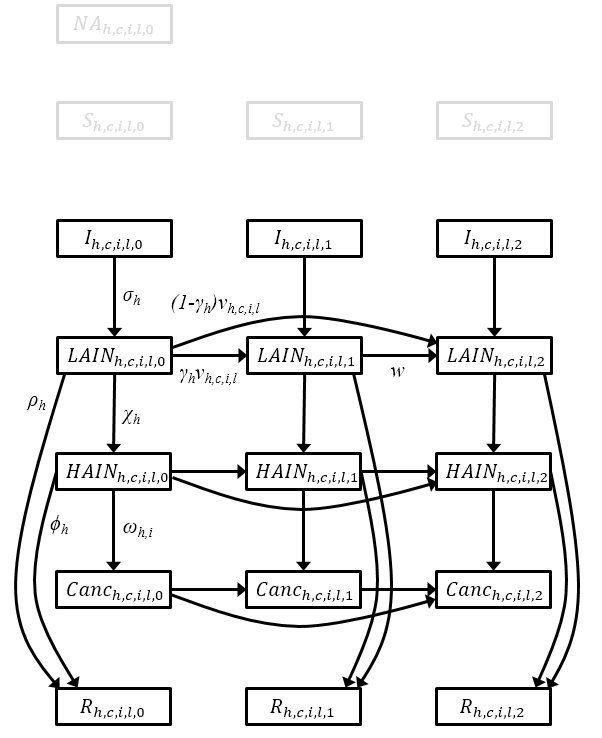


# A2. Epidemiological and demographic parameters

## A2.1. Size of the men who have sex with men (MSM) population

The proportion of men who are MSM was estimated from the third National Survey of Sexual Attitudes and Lifestyles (Natsal-3) [1]. Natsal-3 is the most recent large-scale survey of sexual behaviour in Britain. Individuals aged 16-74, resident in Britain were sampled using a multistage clustered, and stratified probability sample design in 2010-2012, with a response rate of 58%, and individual respondent-level data was weighted for differential selection probability and non-response [2].

MSM were defined as men who reported having had at least one male sexual partner within the last five years, with a partner defined as someone with whom they have had same-sex oral or anal sex or other genital contact (*sam5yrs* in the Natsal-3 data dictionary). This includes oral-genital, anal-genital, and other genital contact, without distinguishing between routes of transmission. This definition was chosen to capture individuals who were more likely to be currently active MSM (and hence be at risk of subsequent HPV infection through same-sex contact), in line with previous publications [1][3]. A total of 190 MSM were binned into age groups of 16-19, 20-24, 25-34, 35-44, 45-54, and 55-74 years. These age bins were based on the default Natsal-3 age groupings of 16-17, 18-19, 20-24, 25-34, 35-44, 45-54, 55-64, 65-74. Age bins 16-17 and 18-19 were combined, and age bins 55-64 and 65-74 were combined. This was done to avoid having small numbers in any age bin, such that any age bin had at least 20 MSM respondents. (There were 8 MSM respondents aged 16-17, and 6 MSM respondents aged 65-74.)

The percentage of all men meeting our definition of being MSM is 2.82% in 16-19 year olds, increases to 2.92% in 20-24 year olds, and peaks at 3.47% in 25-34 year olds, but then subsequently reduces to 2.31% in 35-44 year olds, 2.86% in 45-54 year olds, and 1.68% in 55-74 year olds (although 95% confidence intervals for each age group overlap and variation by age group is only borderline statistically significant). The reduction in older age groups may be due to age cohort effects, or due to individuals who had same-sex partners at younger ages, but stopped at older ages. For simplicity, we assumed that the proportion of MSM remains at 3.47% after age 35 years, and that, in our model, MSM do not lose their MSM status once they acquire it (even though our definition of MSM is based on sexual activity in the past five years rather than sexual identity). Thus, the model only encompasses the 3.47% of the male population in England who will eventually become MSM. However, we assume that some of these MSM may have zero partners at older ages (see below).

Only the MSM population aged 10-74 year olds (divided into 780 monthly birth cohorts) was considered in the model, for comparability to our previous work [4] and to coincide with the upper age bound of Natsal-3 and GUMCAD.

HIV-negative MSM are assumed to be subject to the same age-specific mortality rates as the general population, based on ONS 2012 population data [5]. HIV-positive MSM have an increased monthly mortality risk ratio of 2.18, calculated from an all-cause mortality rate of 4.8 per 1,000 in HIV diagnosed men aged 15-59 compared to 2.2 per 1,000 in all men in 2013 [6].

## A2.2. Age at same-sex debut

Each month, 846 individuals enter the model, based on 3.47% of the Office for National Statistics (ONS) estimate of the number of 10-year-old males in England in year 2012 [5] and the proportion of men who are MSM from Natsal-3 (see above).

All individuals enter the model sexually inactive at age 10. In Natsal-3, MSM reported having their first same-sex experience (involving genital contact) at ages ranging from 5 to 59 years (*agegsam* in the Natsal-3 data dictionary). In line with our previous HPV modelling work [4], individuals aged 12 or younger were assumed to have no sexual partners or risk of HPV infection. Hence the 8 MSM in Natsal-3 reporting same-sex experience before age 12 were assumed to have same-sex debut at age 12. By age 35, 95% of the MSM as surveyed by Natsal-3 have reached same-sex sexual debut. Since few individuals debut at old ages, and given that the MSM percentage peaks in the 25-34 age group, we assume that all MSM in our model (3.47% of the population) debut by age 35. We thus proportionally scaled the percentage debuting each year such that all MSM debut by age 35.

The adjusted cumulative distributions of age at same-sex debut by sexual activity group is shown by dots in the figures below. (Please see the next subsection for the definition of the sexual activity groups.) To remove fluctuations between yearly age groups in the survey data, we fitted log-normal cumulative distribution functions to the proportions that are sexually active (shown by dashed lines).

**Figure.** Cumulative distribution of age at same-sex debut by sexual activity group reported in Natsal-3, and corresponding best fit cumulative lognormal curve to data.

The table below shows the average number of partners between sexual and clinic debut for different activity groups, and the figure shows the rise and decline in the number of individuals in between these two debuts with age.

**Table.** Average number of partners between sexual debut and first attendance at GUM clinic, by activity group.

| **Partnership** | **Low Activity Group** | **Mid Activity Group** | **High Activity Group** |
| --- | --- | --- | --- |
| Same-sex partners | 1.44 | 6.76 | 12.35 |
| Female partners | 0.35 | 0.05 | 0.03 |
| Total partners | 1.78 | 6.81 | 12.38 |

**Figure.** Number of sexually active individuals who are attending clinic, or have not yet started attending clinic but eventually will, for each activity group. Individuals start to sexually debut at age 12, and all individual who eventually will attend clinic do so by age 35.

## A2.3. Same-sex partner change rates

Age-dependent same-sex partner change rates were informed by Natsal-3 data for MSM aged 16-74 years, based on the reported number of same-sex partners in the last 3 months (*sam3mnt* in Natsal-3 data dictionary).

As with our prior work about HPV infection in a heterosexual population [4], the MSM population was further stratified into three activity groups with age-dependent partner change rates, with 80% of the population in the lowest activity group, 15% in the middle group and 5% in the highest group. These percentages were chosen such that MSM in each of the three activity groups account for roughly one-third of the total number of sexual partnerships in the last three months, according to NATSAL-3 data. For the first cutoff, we saw that 76% of MSM had 0-1 partners in the last three months, accounting for 26% of all partnerships. 84% of MSM had 0-2 partners in the last three months, accounting for 36% of all partnerships. Thus, we chose 80% of MSM as the cutoff for the first activity group. Next, we saw that 94% of MSM had 0-5 partners in the last three months, accounting for 63% of all partnerships. 96% of MSM had 0-6 partners in the last three months, accounting for 68% of all partnerships. Thus, we choose 95% (= 80% + 15%) of MSM as the second cutoff. The figure below shows the cutoffs used.

**Figure.** Cumulative proportion of MSM population versus cumulative proportion of same-sex partnerships.

Partner change rates of MSM in the 80% low-activity, 15% medium-activity, and 5% high-activity groups for age groups 16-19, 20-24, 25-34, 35-44, 45-54, and 55-74 were obtained from Natsal-3 [7]. In our model, we assumed that the percentage of individuals who were MSM remained constant after peaking at 3.47% in ages 25-34. However, as mentioned previously, in Natsal-3, the percentage of MSM, defined as having same-sex activity in the last five years, in ages 35-44, 45-54, and 55-74 is lower at 2.31%, 2.86%, and 1.68%, respectively. We assume that difference of these lower percentages with 3.47% (1.16%, 0.61%, and 1.79%, respectively) reflect individuals who were previously sexually active, but are no longer. However, these individuals remain in our model. To account for this, we modify the partner change rates in the model to include these “missing” individuals with zero partners a year.

In order to eliminate boundary effects between age groups, we fitted a log-normal curve to these six data points. Partner change rates for individuals in the lowest activity group peaked at age 29 with 0.56 partners every 3 months, for those in the middle at age 33 with 4.38 partners every 3 months, and for those in the highest at age 34 with 17.10 partners every 3 months. Since Natsal-3 only collected information on respondents age 16 or above, for age groups 12-13 and 14-15, we scaled down partner change rates of age group 16-19 by the fraction of respondents reaching sexual debut by the younger ages, as was done in our previous work [4] in heterosexuals. The figure below shows the fit of our log-normal curves to the data:

**Figure.** Male sexual partner change rates for each activity group from Natsal-3 (orange dots) and best fitting log-normal curves used as inputs for the model (blue lines).

a) Low-activity group

b) Medium-activity group

c) High activity-group

Male sexual partner change rates for MSM at the midpoint of age group for each activity group are shown below.

**Table.** Male sexual partner change rates for each activity group. Partner change rates at the midpoint for each age group are shown.

| **Age** | **Number of male sexual partners over the last 3 months** | | |
| --- | --- | --- | --- |
|  | **Low activity** | **Medium activity** | **High activity** |
| 12-13 | 0.06 | 0.18 | 0.27 |
| 14-15 | 0.16 | 0.48 | 0.75 |
| 16-19 | 0.32 | 1.17 | 1.95 |
| 20-24 | 0.49 | 2.73 | 4.97 |
| 25-34 | 0.56 | 4.24 | 15.21 |
| 35-44 | 0.49 | 4.10 | 14.42 |
| 45-54 | 0.38 | 3.16 | 7.17 |
| 55-74 | 0.25 | 1.88 | 2.63 |

## A2.4. Population mixing

Individuals mix across age, HIV, and activity groups. Mixing across these three attributes was assumed to be independent. An age mixing matrix was constructed using Natsal-3’s most recent male respondent (*rafsmr*) and same-sex partner ages (*r1ptage*) (described below). Assortativity parameters determined mixing across HIV and activity groups. A parameter of 1 indicates completely assortative mixing, and 0 indicates completely proportionate mixing. Given lack of data, assortativeness across activity groups was varied amongst 0.1, 0.5, and 0.9 through scenario analysis, as with our previous heterosexual model [4]. Assortativeness across HIV statuses was also varied amongst 0.1, 0.5, and 0.9. This spans the results from a cross-sectional survey of 11,876 MSM recruited over years 2000 to 2013 in gay social venues in London, which reported that 27% of HIV negative MSM and 30% of diagnosed HIV positive MSM reported exclusively serosorting during unprotected anal sex [8]. For comparison, assortativeness of mixing across age groups for MSM from Natsal-3 data was 0.47. We assumed that there was random mixing between GUM attenders and non-attenders, and between vaccinees and non-vaccinees within the same age, HIV status and activity group.

For mixing across ages, an age-mixing matrix was first constructed using Natsal-3 responses, such that all cells summed to 100%. The matrix was then symmetrized by averaging it with its transpose. The mixing matrix was smoothed with a generalized additive model using a full tensor product smooth (functions gam and te in R). However, to keep the mixing within each age group (the assortativity) equal to Natsal-3 data, the leading diagonal of the original mixing matrix was preserved. The smoothed non-assortative values were then proportionately adjusted, such that their sum matched the total non-assortative mixing found in the original matrix. Each row was divided by the sum across that row, such that each row summed to 100%, as shown in the tables below. The difference in the cost-effectiveness of vaccination using the unsmoothed Natsal-3 mixing matrix instead of the smoothed matrix was limited (e.g. £13,900 versus £14,000 in ICER for vaccinating all 16-40 MSM vs HIV-positive 16-40 MSM at £48/dose). We decided to use the smoothed mixing matrix because cells in the unsmoothed matrix with zeroes (i.e. respondent/partner categories with no Natsal-3 respondents) are likely to be due to small sample size, as opposed to never occurring in the population.

**Table**. Unsmoothed age mixing matrix obtained from Natsal-3. Rows sum to 100%.

|  |  | **Partner** | | | | | | | |
| --- | --- | --- | --- | --- | --- | --- | --- | --- | --- |
|  |  | **12-13** | **14-15** | **16-19** | **20-24** | **25-34** | **35-44** | **45-54** | **55-74** |
| **Respondent** | **12-13** | 0.492 | 0.000 | 0.000 | 0.000 | 0.508 | 0.000 | 0.000 | 0.000 |
|  | **14-15** | 0.000 | 0.321 | 0.679 | 0.000 | 0.000 | 0.000 | 0.000 | 0.000 |
|  | **16-19** | 0.000 | 0.089 | 0.535 | 0.123 | 0.126 | 0.033 | 0.065 | 0.028 |
|  | **20-24** | 0.000 | 0.000 | 0.092 | 0.459 | 0.373 | 0.042 | 0.034 | 0.000 |
|  | **25-34** | 0.009 | 0.000 | 0.064 | 0.253 | 0.536 | 0.061 | 0.040 | 0.037 |
|  | **35-44** | 0.000 | 0.000 | 0.027 | 0.046 | 0.098 | 0.538 | 0.234 | 0.057 |
|  | **45-54** | 0.000 | 0.000 | 0.085 | 0.059 | 0.102 | 0.376 | 0.310 | 0.067 |
|  | **55-74** | 0.000 | 0.000 | 0.055 | 0.000 | 0.141 | 0.136 | 0.099 | 0.569 |

**Table**. Smoothed age mixing matrix. Rows sum to 100%.

|  |  | **Partner** | | | | | | | |
| --- | --- | --- | --- | --- | --- | --- | --- | --- | --- |
|  |  | **12-13** | **14-15** | **16-19** | **20-24** | **25-34** | **35-44** | **45-54** | **55-74** |
| **Respondent** | **12-13** | 0.492 | 0.508 | 0.000 | 0.000 | 0.000 | 0.000 | 0.000 | 0.000 |
|  | **14-15** | 0.005 | 0.321 | 0.339 | 0.202 | 0.072 | 0.028 | 0.020 | 0.012 |
|  | **16-19** | 0.000 | 0.086 | 0.535 | 0.177 | 0.121 | 0.055 | 0.021 | 0.005 |
|  | **20-24** | 0.000 | 0.039 | 0.135 | 0.459 | 0.226 | 0.105 | 0.032 | 0.004 |
|  | **25-34** | 0.000 | 0.011 | 0.070 | 0.173 | 0.536 | 0.137 | 0.060 | 0.014 |
|  | **35-44** | 0.000 | 0.005 | 0.038 | 0.096 | 0.164 | 0.538 | 0.128 | 0.030 |
|  | **45-54** | 0.000 | 0.008 | 0.033 | 0.066 | 0.162 | 0.289 | 0.310 | 0.130 |
|  | **55-74** | 0.000 | 0.008 | 0.013 | 0.014 | 0.062 | 0.115 | 0.220 | 0.569 |

## A2.5. Female-to-male HPV transmission

Some MSM also have sexual partnerships with females (and thus are men who have sex with men and women, or MSMW). The force of HPV infection from female partners is the product of age-specific female HPV prevalence, the age mixing matrix between MSM and females, and the probability of transmission per partnership. Female HPV prevalence in England has been declining since the introduction of female HPV vaccination in 2008 [9]. We used the output of a previously published model of HPV in heterosexuals [4] to predict the decline in female prevalence of HPV 6, 11, 16, and 18 by age group and by year following the introduction of the bivalent and later quadrivalent HPV vaccine in England (see Figure below). We used Natsal-3 data to determine the number of female sex partners for MSM by age group and activity group (*het1yr* in the data dictionary), to which we fitted a log-normal function. We found that MSM in the low activity group for same-sex relationships were more likely to have female partners than MSM in higher activity groups.

**Figure.** Predicted prevalence of HPV 6, 11, 16, or 18 in females in England [4]. Colour gradient for years is in log scale.


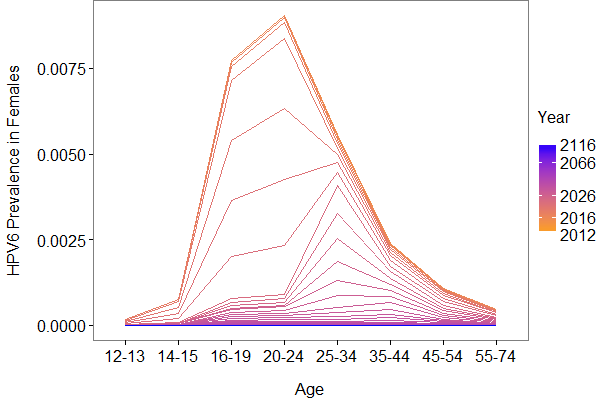

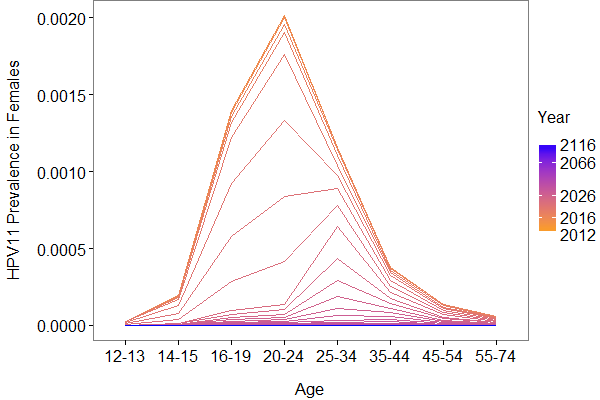


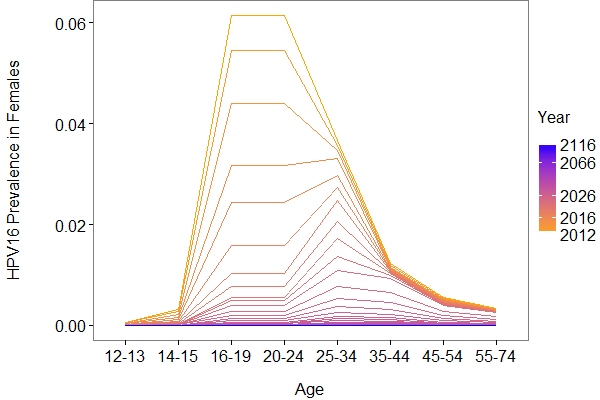

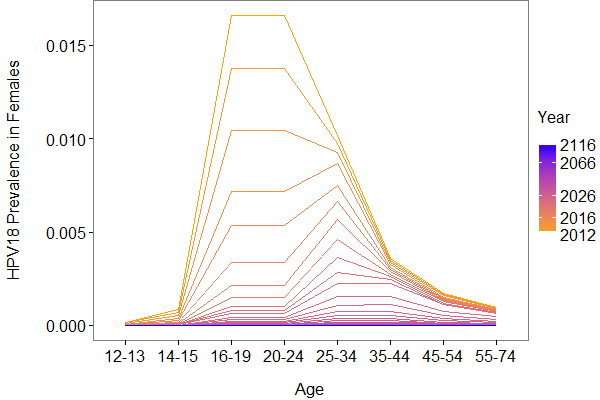


We used Natsal-3 to obtain age mixing of MSM to females, and found an assortativity by age group of 0.40. This age mixing matrix from Natsal-3 was used directly in the model. To determine the probability of transmission per partnership, we chose the probability found in our previous heterosexual model [4] for the heterosexual activity group that was closest in number of total partners by age group to MSM activity groups in our model, by least squared error. Thus, for the low MSM activity group, we used the transmission probabilities associated with the middle heterosexual activity group (0.08 for HPV 6, 0.05 for HPV 11, 0.15 for HPV 16, 0.06 for HPV 18). For the middle and high MSM activity groups, we used the transmission probabilities associated with the high heterosexual activity group (0.02 for HPV 6, 0.02 for HPV 11, 0.08 for HPV 16, 0.03 for HPV 18).

**Table**. Female sexual partner change rates for each MSM activity group. Partner change rates at the midpoint for each age group are shown.

| **Age** | **Number of female sexual partners per year** | | |
| --- | --- | --- | --- |
|  | **Low activity** | **Medium activity** | **High activity** |
| 12-13 | 0.006 | 0.014 | 0.05 |
| 14-15 | 0.021 | 0.047 | 0.16 |
| 16-19 | 0.052 | 0.062 | 0.36 |
| 20-24 | 0.033 | 0.062 | 0.49 |
| 25-34 | 0.009 | 0.062 | 0.48 |
| 35-44 | 0.001 | 0.062 | 0.36 |
| 45-54 | 0.000 | 0.062 | 0.25 |
| 55-74 | 0.000 | 0.062 | 0.14 |

**Figure**. Number of female partners per year by MSM in the low activity group.

**Figure**. Number of female partners per year by MSM in the middle activity group.

**Figure**. Number of female partners per year by MSM in the high activity group.

**Table**. Age mixing of MSM and females. This table was not smoothed. Rows sum to 100%.

|  |  | **Female Partner** | | | | | | | |
| --- | --- | --- | --- | --- | --- | --- | --- | --- | --- |
|  |  | **12-13** | **14-15** | **16-19** | **20-24** | **25-34** | **35-44** | **45-54** | **55-74** |
| **MSM**  **Respondent** | **12-13** | 1.000 | 0.000 | 0.000 | 0.000 | 0.000 | 0.000 | 0.000 | 0.000 |
|  | **14-15** | 0.000 | 0.466 | 0.534 | 0.000 | 0.000 | 0.000 | 0.000 | 0.000 |
|  | **16-19** | 0.000 | 0.029 | 0.971 | 0.000 | 0.000 | 0.000 | 0.000 | 0.000 |
|  | **20-24** | 0.000 | 0.000 | 0.569 | 0.195 | 0.236 | 0.000 | 0.000 | 0.000 |
|  | **25-34** | 0.000 | 0.000 | 0.127 | 0.388 | 0.282 | 0.203 | 0.000 | 0.000 |
|  | **35-44** | 0.000 | 0.242 | 0.000 | 0.471 | 0.070 | 0.089 | 0.127 | 0.000 |
|  | **45-54** | 0.000 | 0.000 | 0.000 | 0.000 | 0.410 | 0.365 | 0.225 | 0.000 |
|  | **55-74** | 0.000 | 0.000 | 0.000 | 0.000 | 0.625 | 0.181 | 0.195 | 0.000 |

**Table**. Total (male and female) sexual partner change rates for each MSM activity group. Partner change rates at the midpoint for each age group are shown.

| **Age** | **Total number of sexual partners per year** | | |
| --- | --- | --- | --- |
|  | **Low activity** | **Medium activity** | **High activity** |
| 12-13 | 0.2 | 0.7 | 1.1 |
| 14-15 | 0.7 | 2.0 | 3.2 |
| 16-19 | 1.3 | 4.7 | 8.2 |
| 20-24 | 2.0 | 11.0 | 20.4 |
| 25-34 | 2.2 | 17.0 | 61.3 |
| 35-44 | 2.0 | 16.5 | 58.0 |
| 45-54 | 1.5 | 12.7 | 28.9 |
| 55-74 | 1.0 | 7.6 | 10.7 |

## A2.6. GUM attendance and anogenital warts diagnoses

MSM attendance rates at GUM clinics (and therefore eligibility for vaccination) were estimated from 2009-2012 data in the Genitourinary Medicine Clinic Activity Dataset (GUMCAD), an anonymised patient-level electronic dataset collecting information on diagnoses made and services provided by GUM clinics [10], stratified by known HIV status. Data completeness of GUM attendances reported to GUMCAD at PHE is likely to be high, as reporting to GUMCAD is mandatory for all commissioned sexual health services, including anogenital warts treatment. However, we did not include MSM attending “level 2 providers” or sexual health services outside of GUM clinics such as enhanced general practices, sexual and reproductive health clinics and young people’s clinics.

Based on Natsal-3 data, 52% of MSM reported ever having attended a GUM clinic. These data also indicated that 48%, 70%, and 79% of MSM in the low, middle, and high activity groups, respectively, ever attended, such that a greater fraction of higher risk groups attend. We consider these individuals to be clinic attenders. We assumed that 77.4% of MSM acquiring HIV are diagnosed, based on multi-parameter evidence synthesis modelling by Public Health England [11]. We assume that all diagnosed HIV-positive MSM attend GUM clinics, so HIV-positive MSM attend clinics at a weighted average of diagnosed and undiagnosed rates. MSM are assumed to be offered three doses of HPV vaccine, with first dose uptake of 89% and completion rates of 69% for dose two and 49% for dose 3, based on previously reported MSM hepatitis B vaccination completion rate reported by a London Hospital [12]; surveys of GUM-attending MSM suggest that acceptability of HPV vaccination will be similarly very high [13].

GUMCAD was used to determine the frequency of attendance for those who do attend clinic. For the age categories of <25 years old, 26-35 years old, 36-45 years old, and >45 years old, the number of MSM attending between 2009 and 2012 and the number of revisits within a period of 2 years after their initial visit were available. Assuming that HIV-positive MSM attend twice a year [10,14,15], the average HIV-negative MSM has 0.37 reattendances 1 to 12 months after their initial visit, and 0.65 reattendances 13 to 24 months after their initial visit. This reattendance rate did not vary between the age groups.

In Natsal-3, the percentages of MSM respondents ever having attending a GUM clinic was 23.2%, 38.5%, 56.9%, 56.7%, 39.4%, 36.6%, for the age groups 16-19, 20-24, 25-34, 35-44, 45-54, and 55-74 years old, respectively. Since this percentage peaks in the age group of 25-34, and since we assume that all MSM sexually debut by age 35, we assume that all MSM that will ever attend a GUM clinic will have started by the same age, 35. Natsal-3 did not ask respondents about their age at first GUM clinic attendance. Thus, the number of GUM attendees in 2012 by HIV-negative individuals in GUMCAD was used. The rise in the number of attendees by age is lower than the cumulative distribution of HIV-negative individuals who have started attending clinics, because some individuals do not reattend each year. From our previous analysis, we assumed that only 65% attend a subsequent year, and thus, as an approximation, the number of attendees was inflated by 100% – 65% = 35% to estimate the number of HIV negative MSM who have started attending clinics. A log-normal cumulative distribution function was then fit to this data up to age 20, with the assumption that all clinic attenders start doing so by age 35. As a check, we find that this log-normal cumulative fit approximates the percentages of clinic attenders from Natsal-3.

**Figure**. Proportion of clinic attendees who have started attending clinics.

The relationship between sexual activity and age at first GUM clinic attendance could not be determined in Natsal-3, since respondents were not asked about their age at first GUM clinic attendance. If MMC respondents (which are skewed towards higher sexual activity groups) are partitioned into thirds by sexual activity, no pairwise statistical differences in the cumulative distribution of age at first GUM clinic attendance are found between the thirds by the Kolmogorov–Smirnov test. Thus, in our model, all sexual activity groups have the same age at first clinic attendance distribution.

To calculate the yearly rate of HIV-negative clinic attendees, we divided the number of GUM attendees at each age in GUMCAD by the number that had started attending clinic at each age in our model, multiplied by 1.37 to account for reattendances in the same year. The monthly probability of clinic attendance for all (diagnosed and undiagnosed) HIV-positive MSM is the weighted average of the monthly probability of clinic attendance for diagnosed HIV-positive MSM in GUMCAD (who attend twice a year) and the monthly probability of clinic attendance for undiagnosed HIV-positive MSM (who attend at the HIV-negative MSM rate).

In HPV 6 and 11 models, individuals with warts present themselves to GUM clinics and hence may receive HPV vaccination. To ensure that the total number of clinic attenders and attendances remains the same across models, for HPV 6 and 11 models, two adjustments were made. First, HIV-negative MSM who attend clinic for the first time because of wart symptoms count towards the distribution of age at first clinic attendance in our model (see Appendix A2.2). Thus, the distribution of age at first clinic attendance remains the same regardless of HPV type. We do not need this adjustment for HIV-positive MSM, as we assume that all HIV-positive MSM attend clinic. Second, the clinic attendance rate is reduced by the number of warts attendances. That is,

Adjusted attendance rate = (GUMCAD attendance rate * population size – warts attendances)/population size

These individuals are then vaccinated based on their GUM clinic attendance rate. We assume that all recorded anogenital warts episodes in MSM involve a GUM clinic visit and hence an offer of vaccination. Hence we do not consider about 5% of recorded anogenital warts episodes in all men and women that involve GP visits only [16].

**Figure.** GUM clinic attendance rates by age for HIV-positive and HIV-negative MSM.

## A2.7. HIV status

Individuals in the model were stratified by HIV status. Being HIV-positive was defined as being serologically HIV-positive, regardless of whether the HIV status has been diagnosed. The age-dependent rate of seroconversion from HIV-negative to HIV-positive was estimated by fitting to HIV prevalence data for diagnosed and undiagnosed HIV among MSM in the UK [17]. HIV prevalence was obtained from Survey of Prevalent HIV Infections Diagnosed (SOPHID) and HIV and AIDS Reporting System (HARS) for those resident in England [18]. These figures were then inflated by 22.65% to account for the estimated proportion of HIV infections that are believed to be undiagnosed in MSM, based on statistical modelling of census, surveillance and survey data [19].

We used data in the 2008 UK Gay Men’s Sex Survey research report [20] to determine the relationship between activity group and HIV status. This was a cross-sectional survey of 7,461 MSM living in the UK, with 89.8% living in England, and a median age of 35 years old (range 14 to 83 years). Results from this survey were binned by number of partners in the last 12 months into the following categories: none, 1, 2 to 4, 5 to 12, 13 to 29, and more than 30. Based on NATSAL-3 data, we labeled individuals with 0 to 12 partners in the last year as in the low activity group, individuals with 13 to 29 partners as in the middle activity group, and individuals with more than 30 as in the high activity group. From the survey, 9.5% (95% CI 8.8% - 10.3%), 12.9% (10.7% - 15.0%) and 22.6% (19.6% - 25.5%) of the individuals in low, middle, and high activity group were living with diagnosed HIV, respectively. Because this sex survey did not capture the percentage of undiagnosed HIV in respondents, we calculated the ratio of percentages of diagnosed HIV between the activity groups, and assumed that this ratio is the same for undiagnosed HIV between the activity groups. Thus, individuals in the middle and high activity groups have a HIV force of infection that is 1.35 times and 2.37 times greater than those in the low activity group, respectively.

For simplicity, it was assumed that all HIV-positive MSM have the same increased mortality risk and HPV progression rates regardless of whether their HIV has been diagnosed. HIV prevalence peaks at 12.7% at age 46, likely due to cohort effects. Hence we assume that no new HIV infections occur in older ages. A quartic model (fourth-degree polynomial) was fitted to the prevalence of HIV for MSM in GUMCAD aged 16-46 years to smooth out fluctuations (e.g. declines in prevalence from age 34 to 35). We then calculated the age-dependent force of infection that is needed to generate this prevalence. We assumed that reductions in the observed prevalence of HIV-positive individuals after age 46 are due to cohort effects, and assumed that no new HIV infections occur after age 46, and so the prevalence of HIV-positive individuals after age 46 only changes due to death. This assumption does not have a large effect on model predictions of vaccine impact since we only examined scenarios offering vaccination to MSM up to age 40 years.

The figures below show the prevalence of HIV in the MSM population and the monthly force of HIV infection that was used in the model.

**Figure.** Prevalence of HIV in the MSM population according to GUMCAD (orange dots) and using with a best-fitting quartic model up to age 46 years (blue line).

**Figure.** Age-dependent force of HIV infection used in the model, by activity group.

## A2.8. Vaccine efficacy

Vaccine efficacy was estimated from the FUTURE II trial of HPV vaccines in men [21]. This was a randomized, placebo-controlled, double-blind trial of quadrivalent vaccination in 4065 men aged 16-25 years from 18 countries. We used efficacy against persistent infection, defined as detection of the same HPV type in an anogenital swab or biopsy specimen collected on at least two consecutive visits at least six months apart. Efficacy was taken from the naïve-to-relevant-type (NRT) cohort, defined as those who received at least one dose of vaccine or placebo, and were PCR negative and seronegative to the HPV type analyzed at time of enrolment. We use NRT instead of intention-to-treat (ITT) because our model already accounts for lack of therapeutic efficacy in individuals who are infected when they take their vaccine. Vaccine protection was assumed to be all-or-nothing (i.e. vaccinated individuals were assumed to be either completely immunised or to gain no vaccine protection at all, rather than to gain partial protection).

The confidence intervals for HPV-6/11/16/18 type-specific vaccine efficacy were wide and overlapping for HPV-6 and HPV-11, as well as HPV-16 and HPV-18. Hence we obtained a combined efficacy value for HPV-6/11, by weighting type-specific efficacy for HPV-6 and HPV-11 by their attributable fraction of disease endpoint. We similarly obtained a combined efficacy value for HPV-16 and HPV-18. The final vaccine efficacy values used in the model for HPV-6/11 and HPV-16/18 were 77.6% (95% CI: 61.4% to 87.0%) and 63.7% (95% CI: 44.5% to 76.2%), respectively. Type-specific vaccine efficacy in all men was used, since the breakdown for MSM only was not available and there was no statistical significant difference in vaccine efficacy against HPV-6/11/16/18-related persistent infection between heterosexual and MSM. HIV-positive males respond well immunologically to quadrivalent HPV vaccination, so vaccine efficacy was assumed to be independent of HIV status [22]. We used vaccine efficacy against persistent infection from the naïve-to-relevant type cohort in trials of Gardasil in males.

We did not consider cross protection to non-vaccine HPV types as this was not reported in the trials. The lower attributable fraction of HPV-related cancers in males to high risk HPV types other than HPV-16/18 is lower than in females (~20% of anal cancers, compared with 20-30% of cervical cancers) [23]. As there is currently no evidence that a two-dose regime is sufficient in adult males, we only considered a three-dose vaccine schedule in the base case. There are currently no efficacy data in males for the bivalent vaccine, so we assumed that it would have equivalent efficacy to the quadrivalent against HPV-16/18, but give no protection against HPV-6/11.

## A2.9. HPV-related cancer incidence

ONS does not record anal cancer incidence by MSM or HIV status. The age-standardized incidence of all male anal cancers in England was averaged across 2008-2012 ONS data (ICD-10 code C21). This was then inflated to represent the relative risk of anal cancer in HIV-negative and HIV-positive MSM compared to all men. For this calculation, we used data from a systematic review covering studies up to November 2011 [24], which found anal cancer incidence in HIV-negative MSM was 5.1 (95%CI 0–11.5) per 100,000 person-years, and 77.8 (95% CI 59.4–96.2) per 100,000 person-years in HIV-positive MSM after antiretroviral therapy introduction (1996). Increased risk in MSM by HIV status was then calculated by computing the ratio of these rates over that of all males in ONS (1.35 per 100,000 person-years). The calculated risk ratio was thus 3.78 for HIV-negative MSM and 57.63 for HIV-positive MSM compared to men in England.

**Table.** Estimated incidence of anal cancer in males, HIV-negative MSM, and HIV-positive MSM in England.

| **Age** | **Incidence in Males**  **(per 100,000**  **person-yrs)** | **Incidence in HIV-negative MSM (per 100,000 person-yrs)** | **Incidence in HIV-positive MSM (per 100,000 person-yrs)** |
| --- | --- | --- | --- |
| 20-24 | 0.0 | 0.0 | 0.0 |
| 25-29 | 0.1 | 0.2 | 2.9 |
| 30-34 | 0.1 | 0.2 | 2.9 |
| 35-39 | 0.3 | 1.2 | 18.7 |
| 40-44 | 0.7 | 2.7 | 41.8 |
| 45-49 | 1.3 | 4.8 | 73.5 |
| 50-54 | 2.0 | 7.5 | 113.8 |
| 55-59 | 2.4 | 9.1 | 138.3 |
| 60-64 | 3.7 | 13.8 | 210.3 |
| 65-69 | 4.0 | 15.1 | 230.5 |
| 70-74 | 4.9 | 18.5 | 282.4 |

Age-specific incidence data for the other cancers in men in England were obtained from ONS 2008-2012 data using ICD-10 codes C60 for penile, C09-10 for oropharyngeal (C09 malignant neoplasm of tonsil, C10 malignant neoplasm of oropharynx), C01-05 for oral cavity and C32 for laryngeal cancers.

As with anal cancer, we may suspect that incidence of these cancers is different in MSM compared to other men, and particularly in MSM attending GUM clinics, due to different partner acquisition and/or sexual behaviour patterns. However, the HIMS study conducted in Brazil, Mexico and the USA found no statistically significant associations in HIV negative men between penile or scrotal HPV, and status as MSM or MSW (men who have sex with women) [25]. The same study estimated the incidence and clearance of HPV using oral rinse-and-gargle samples and found that the acquisition of oral oncogenic HPV was not associated with reported sexual behaviours (including number of partners and oral sex behaviour) [26]. After adjusting for covariates, the same study also reported no statistically significant increased risk of oral HPV and reporting 20 or more lifetime sexual partners (adjusted OR 1·73, 95% CI 0·87–3·45). Therefore, we assumed that oropharyngeal (as well as oral cavity and laryngeal) cancer incidence was similar for MSM and heterosexual men.

HPV-related cancer risk for penile, oropharyngeal, oral and laryngeal cancers in MSM was adjusted based on their HIV status. For oropharyngeal and oral cancers, HIV positive men were at 2.32 fold increased risk of progression to these cancers compared with HIV negative men according to a recent meta-analysis [27]. For penile and laryngeal cancer, the risk was 4.42 fold and 2.72 fold higher respectively [27].

## A2.10. HPV attributable fraction in cancer

The proportion of penile cancers attributable to HPV, and to HPV-16 and 18 in particular, was obtained from a literature review by Miralles-Guri et al. [28] For oropharyngeal, oral and laryngeal cancers, we obtained the same breakdown from the European figures reported in a review by Kreimer et al. [29] We used European-specific estimates for oropharyngeal, oral cavity and laryngeal cancers because of the geographical heterogeneity in HPV prevalence in these cancers, which is partly thought to be attributable to the regional differences in distribution of risk factors other than HPV infection. For example, North America tends to have higher percentages attributable to HPV, and within Europe, Northern Europe tends to have higher percentages. Note that for oropharynx notably different and higher percentages have been reported in other publications [30], which may partly reflect increases over time as well as variations between populations studied in the anatomical sites included in the definition of oropharynx and in the prevalence of the other causes of oropharyngeal cancer (especially smoking and alcohol).

The tables below summarise approximate estimates of the number of cancers related to HPV and HPV-16/18 in all men, and in MSM aged 10-74 years (i.e. the modelled population).

**Table.** Estimated number of cancers related to HPV and HPV-16/18 in all males, based on the average annual cases for 2008-2012 from ONS cancer registration data.

| **Site** | **% HPV** | **% HPV-16/18** | **Annual number of cancers in men aged 10-74 yrs** | | | **% of all HPV-16/18-related cancers in men aged 10-74 yrs** | |
| --- | --- | --- | --- | --- | --- | --- | --- |
|  |  |  | **All** | **HPV-related** | **HPV-16/18-related** | **Excluding larynx** | **All** |
| Penis | 47.0% | 35.1% | 309 | 145 | 108 | 15% | 12% |
| Oropharynx | 28.2% | 25.0% | 837 | 236 | 209 | 29% | 23% |
| Anus | 84.0% | 66.0% | 269 | 226 | 177 | 25% | 19% |
| Larynx | 21.3% | 17.8% | 1126 | 240 | 200 | - | 22% |
| Oral cavity | 16.0% | 15.2% | 1477 | 236 | 225 | 31% | 24% |
| Total |  |  | 4018 | 1083 | 920 |  |  |

**Table.** Estimated number of cancers related to HPV and HPV-16/18 in MSM aged 10-74 years, based on the average annual cases for 2008-2012 from ONS cancer registration data. This assumes that HPV prevalence and type distribution is the same in cancers in MSM and non-MSM males.

| **Site** | **% HPV** | **% HPV-16/18** | **Annual number of cancers in MSM aged 10-74 yrs** | | | **% of all HPV-16/18-related cancers in MSM aged 10-74 yrs** | |
| --- | --- | --- | --- | --- | --- | --- | --- |
|  |  |  | **All** | **HPV-related** | **HPV-16/18-related** | **Excluding larynx** | **All** |
| Penis | 47.0% | 35.1% | 12 | 6 | 4 | 5% | 5% |
| Oropharynx | 28.2% | 25.0% | 31 | 9 | 8 | 9% | 8% |
| Anus | 84.0% | 66.0% | 99 | 83 | 66 | 76% | 70% |
| Larynx | 21.3% | 17.8% | 41 | 9 | 7 | - | 8% |
| Oral cavity | 16.0% | 15.2% | 54 | 9 | 8 | 10% | 9% |
| Total |  |  | 238 | 115 | 93 |  |  |

# A3. **Scenario selection and cal**ibration

## A3.1. Model calibration

5,000 HPV-16 and 5,000 HPV-18 models with varying scenarios were fitted to anal cancer incidence (from ONS) and HPV prevalence (from Mortimer Market Centre (MMC), see next section), stratified by both age and HIV status. Fitting was conducted using a two-step process. In the first step, all 5,000 HPV-16 and 5,000 HPV-18 disease models were fitted to minimise the sum of squared residuals between model HPV prevalence and HPV prevalence from MMC. The top 5% of best-fitting models (with the lowest sum of squared residuals) were selected, and an age-dependent exponential HGAIN → Cancer rate was calculated for each model that best match ONS cancer data. The HGAIN → Cancer rates were then averaged, and all 5,000 disease cancer models for each HPV type were then refitted using both MMC HPV prevalences and ONS cancer data (with both data sets contributing equally to the sum of squared residuals). The top 20% of best-fitting models were then used for subsequent vaccination analysis.

5,000 HPV 6 and 5,000 HPV 11 models with varying scenarios were fitted by minimising the sum of squared residuals between modelled warts incidence and 2009-2012 average age-specific first-time warts incidence in GUMCAD for both HIV-positive and negative MSM. We did not use primary data on HPV-6/11 prevalence since any available data would have much smaller sample sizes than warts incidence in GUMCAD. The top 20% of best-fitting models were then used for subsequent vaccination analysis.

In calibrating models, female HPV-type specific prevalences in the year 2012 as estimated in previous work [4] were used.

## A3.2. HPV prevalence

Type-specific HPV prevalence was estimated from a cross-sectional prevalence study of 511 MSM in age groups 18-25, 26-30, 31-35, and 36-40 attending the Mortimer Market Centre sexual health clinic in London between October 2010 and July 2012 (57). For HPV-16/18, individuals were assumed to be positive for an HPV strain when the DNA of this strain was detected in an anal sample. 430 HIV-negative MSM and 24 HIV-positive contributed anal samples.

The MSM sampled in the MMC cohort are more sexually active than the general England MSM population. 6%, 12%, and 82% of the MMC MSM were in the low, middle, and high activity groups, respectively, as defined by NATSAL-3 data. Because of the few number of HIV-positive individuals sampled, the distribution of activity groups were assumed to be the same amongst HIV-negatives and HIV-positives. To fit HPV prevalence in the model to that in the MMC population, HPV prevalence in the model was first reweighted to resemble the MMC population, where model prevalence amongst the low, middle, and high activity group contributed 6%, 12%, and 82% to the average, respectively.

The reweighted HIV-negative HPV prevalence in the model was fitted to the MMC age-categorized HIV-negative HPV prevalence. Because of the few number of HIV-positive MSM in the MMC cohort, we multiplied the MMC age-categorized HIV-negative prevalence by the increased HPV risk ratio between HIV-positive and HIV-negative individuals in the MMC cohort. For HPV-16, this ratio was 2.92, and for HPV-18, this ratio was 1.99. The reweighted HIV-positive HPV prevalence in the model was then fitted to these values.

## A3.3. Scenarios

*HPV clearance rates.* A study of 247 HIV-positive Montreal MSM found clearance rates for different HPV types to vary around 12 to 20 cleared episodes/1000 person-months [31]. Another study of 1159 HIV-negative men in USA, Brazil, and Mexico found that clearance rates for different HPV types varies approximately 80 to 150 cleared episodes/1000 person-months [32]. For HIV-positive MSM, the HPV clearance rate was varied amongst 8, 12, 16, 20, and 24 cleared episodes/1000 person-months. For HIV-negative MSM, the HPV clearance rate was varied amongst 50, 80, 110, 140, and 170 cleared episodes/1000 person-months. Clearance was assumed to happen at a constant rate, resulting in an exponential fall in HPV persistence with time.

*Natural immunity.* Details of subsequent natural immunity are relatively unknown (58). Since SIS, SIR, and SIRS structures are all plausible, the structural uncertainty was accounted for by varying mean duration of natural immunity, separately for HIV-negative and HIV-positive MSM, amongst 0, 3, 10, 20 years and lifelong. We also added the constraint that the duration of natural immunity of HIV-negative MSM must be longer or equal to that of HIV-positive MSM. For 3-20 years, waning of natural immunity was assumed to happen at a constant rate, resulting in an exponential fall in HPV persistence with time.

*Proporton of HPV-infected individuals with anogenital warts.* To our knowledge, there is no study on the proportion of HPV-infected, HIV-negative individuals symptomatic with warts. Thus, this value was varied amongst 10%, 20%, and 30% for HIV-negative MSM as with our female HPV vaccination modelling which matched age-specific warts incidence with a HPV-6/11 UK seroprevalence study [4]. Since there was no evidence that HPV-infected, HIV-positive individuals differ in symptoms, this value was also varied amongst 10%, 20%, and 30% for HIV-positive MSM

*Proportion of anogenital warts due to HPV-6 or HPV-11.* Studies on HPV warts causality are limited, as swabbing wart surfaces does not appear to determine causative types. For the following calculations, in individual with multiple HPV types, causality was assigned to the more prevalent type. Hence individuals with warts infected with both HPV-6 and HPV-11 have their warts ascribed to HPV-6. A study in the UK of 23 warts biopsies found that 90% were HPV-6/11-related, of which 15% were attributable to HPV-11 [35]. An older UK study of 31 warts biopsies found that 10% of HPV-6/11-related warts were attribute to HPV-11 [36], while an older US study of 65 warts biopsies found 25% [37]. The percentage of HPV-6/11-related warts caused by HPV-11 were varied amongst these values in scenario analysis.

*Proportion of anal cancer cases due to HPV-16 or 18.* A study testing of 169 invasive anal cancer specimens from Europe [34] found that 87.6% (95% CI 81.6-92.1) were HPV-positive. Of these 80.7% were HPV-16-positive (but only 75.8% had HPV-16 and no other type), while 3.6% were HPV-18-positive (but only 3.4% had HPV-16 and no other type. We used these results to generate three scenarios for the causal HPV types in anal cancer cases, shown below.

**Table.** Scenarios used for the proportion of anal cancer cases due to HPV-16/18.

|  | **Proportion of anal cancers due to HPV-16/18** | **Proportion of HPV-16/18 anal cancers due to HPV-18** |
| --- | --- | --- |
| **Use singly typed specimens only** | 87.6% x (75.8% + 3.4%) = 69.4% | 3.4%/(75.8% + 3.4%) = 4.3% |
| **Use both singly and multiply typed specimens** | 87.6% x (80.7% + 3.6%) = 73.8% | 3.6%/(80.7% + 3.6%) = 4.3% |
| **Use both singly and multiply typed specimens, but make a correction for double counting** | 87.6% x 80.7% + 87.6% x 3.6% x (1-87.6% x 80.7%) = 71.6% | 1.1%*/(80.7% + 1.1%) = 1.3% |

*Here 1.1% is the proportion of HPV-18 specimens after correcting for double-counting, i.e. 3.6% x (1-87.6% x 80.7%) = 1.1%

**Table.** Scenarios that are varied for each model. Parameters affecting only HPV-6/11 or HPV-16/18 are italicized.

| **Parameter** | **HIV-negative** | **HIV-positive** |
| --- | --- | --- |
| Natural immunity duration | Lifelong, 20, 10, 3, 0 years | Lifelong, 20, 10, 3, 0 years |
| HPV clearance rate (cleared episodes/1000 person-months) | 50, 80, 110, 140, 170 | 8, 12, 16, 20, 24 |
| *Percentage of HPV-6/11-infections causing warts* | *10%, 20%, 30%* | *10%, 20%, 30%* |
| Activity group assortativeness | 0.1, 0.5, 0.9 | |
| HIV assortativeness | 0.1, 0.5, 0.9 | |
| *Percentage of HPV-6/11-related warts due to HPV-11* | *10%, 15%, 25%* | |
| *Proportion of anal cancers due to HPV-16/18, and percentage of HPV-16/18-related anal cancers due to HPV-18* | *(69.4%, 4.3%), (73.8%, 4.3%), (71.6%, 1.3%)* | |
| Transmission probability per partnership | Governed by low-activity or high-activity partner | |

5,000 scenarios for each HPV type were selected with Latin hypercube sampling.

## A3.4. HPV transmission

For each HPV-16/18 and HPV-6/11 scenario, age-specific equilibrium outcomes in the models were fitted to their targets (HPV prevalence, anal cancer incidence, warts incidence) by varying two parameters that governed HPV transmission per partnership (see Table below). As with our previous model of heterosexual HPV transmission [4], we define *p* to be the HPV transmission probability between two low-activity infected and susceptible individuals, and α to be the fraction of that transmission probability a partnership in the higher activity group faces. Thus, partnerships of two medium-activity individuals have *p*α transmission probabilities, and partnerships of two high-activity individuals have a *p*α^2^ transmission probability. Both parameters are bounded between 0 and 1. α upper-bounded by 1, since individuals in higher-activity group will likely have fewer sexual acts per partnership, so HPV transmission probability in that partnership alone is likely than lower longer-term partnerships. Because individuals in partnerships might be in different activity groups, transmission probabilities can be governed by the high-activity or low-activity partner, which is considered through scenario analysis. Thus, given lack of data on HPV transmission partnership, parameters governing transmission were varied to best fit the data.

For each scenario, numerical fitting of HPV transmission parameters α and p was done by minimizing a weighted sum of squared errors (SSE) measure, using the Brent method [31]. For HPV-6 and 11, the SSE between model prediction and observed data of warts incidence for each yearly age group was first calculated, separately for HIV-positive and negative individuals. The two SSE were then weighted by the variance of the observed data in that HIV status, and then summed, such that each HIV status contributes equally to half of the overall goodness of fit metric. This weighting ensures the model fits to HIV-positive data, despite having fewer individuals in it. For HPV-16 and 18, the SSE between model prediction and observed data of anal HPV prevalence and anal cancer incidence for each yearly age group were calculated, separately for HIV-positive and HIV-negative individuals. The four SSE were then weighted by the variances of the observed data and the number of datapoints such that each equally contribute to a quarter of the goodness of fit.

**Table.** Transmission probability governance.

| a) Governed by high-activity partner | | | | |
| --- | --- | --- | --- | --- |
|  |  | Uninfected | | |
|  |  | Low-activity | Med-activity | High-activity |
| Infected | Low-activity | *p* | *pα* | *pα^2^* |
|  | Med-activity | *pα* | *pα* | *pα^2^* |
|  | High-activity | *pα^2^* | *pα^2^* | *pα^2^* |
|  |  |  |  |  |
| b) Governed by low-activity partner | | | | |
|  |  | Uninfected | | |
|  |  | Low-activity | Med-activity | High-activity |
| Infected | Low-activity | *p* | *p* | *p* |
|  | Med-activity | *p* | *pα* | *pα* |
|  | High-activity | *p* | *pα* | *pα^2^* |

## A3.5. Natural history parameters for anal cancer

The rates of progression and regression of HPV infection to AIN and anal cancer were based on studies reviewed in a systematic review of the natural history of anal HPV infection [24]. Because of lack of data, anal cancer parameters for HPV-16 and 18 were assumed to be identical other than progression of HGAIN to invasive anal cancer.

*Rate of progression from Infected to LGAIN (Infected → LGAIN)*

The Infected → LGAIN rate for HIV-negative MSM was calculated from the placebo arm of the quadrivalent vaccine in MSM [38]. We used the rate of progression from persistent infection to AIN for HPV-16/18, from the per-protocol efficacy population. The per-protocol efficacy population was chosen to be certain that these individuals did not have HPV prior to start of the trial, which might otherwise inflate the Infected → LGAIN rate.

Of the 24 cases with HPV-16/18 infection, 10 transitioned into AIN during the course of the study. Accounting for the fact that persistence in the study was defined as consecutive detection of HPV at least 4 months apart, the calculated rate of Infected → LGAIN for HIV-negative is 0.36/person-year (10 cases in 27.4 person-years).

Data on the Infected → LGAIN rate for HIV-positive MSM was not found in recent studies. Thus, we used data from a 1998 study by Palefsky et al. which measured AIN progression in 143 HIV positive and 131 HIV negative individuals for 2 years and also the number of HPV strains they were infected with [39]. Progression here was not distinguished between Infected → LGAIN and LGAIN → HGAIN. The rate of progression in HIV positive individuals infected with either a single or multiple HIV strains was 0.58/person-year (95 cases in 165 person-years), and the rate of progression in HIV negative individuals was 0.30/person-year (32 cases in 106 person-years). Thus, the rate of Infected → LGAIN for HIV-positive MSM is 1.9 times that of HIV-negative MSM. The rates of progression in HIV negative individuals we estimated were similar to those in the placebo arm of the quadrivalent vaccine trial in MSM (0.36/person-year [38] vs 0.30/person-year [39]).

*Rate of progression from LGAIN to HGAIN (LGAIN → HGAIN)*

Data on the LGAIN → HGAIN rate for MSM by HIV status was not found in recent studies. Thus, we used data from a 1998 study by Palefsky et al. which measured incident HSIL (high-grade squamous intraepithelial lesion) in 277 HIV positive and 211 HIV negative individuals with 4 years of follow-up [39]. Of 135 HIV-positive MSM and 34 HIV-negative with LSIL (low-grade squamous intraepithelial lesion) or ASCUS (atypical squamous cells of undetermined significance) at baseline, 62 and 11 had HSIL by either cytology or histology within 4 years, for yearly rate of progression of 0.149 (416 person-years) and 0.096 (114 person-years), respectively. Thus, the rate of LGAIN → HGAIN for HIV-positive MSM is 1.5 times that of HIV-negative MSM.

*Rate of progression from HGAIN to cancer (HGAIN → cancer)*

Annual rates estimated from Machalek et al. [24], of 1 in 377 in HIV-positive MSM and 1 in 4196 in HIV-negative MSM, were used.

*Anal HPV clearance*

For HPV-16 and 18, only the natural clearance rates of HPV at any stage of anal cancer progression (r_tot_) has been measured (see A3.3. Scenarios, HPV clearance rates). The individual natural clearance rates of HPV infected without any AIN (r_I_), HPV with LGAIN (r_L_), and HPV with HGAIN (r_H_) have not been independently investigated. In our model, r_tot_ is varied through scenario analysis. The other three parameters were solved in relation to r_tot_ through several assumptions.

First, the total number of individuals who exit any HPV infected state through either clearance or death must be related to the number of individuals who exit each HPV infected stated through clearance or death (and ignoring the cancer state due to its rarity, for simplicity). This results in the following:

$l_{tot}N_{tot}\left( r_{tot}+d \right)=l_{I}N_{tot}\left( r_{I}+d \right)+l_{L}N_{tot}\left( r_{L}+d \right)+l_{H}N_{tot}\left( r_{H}+d \right)$ (1)

N_tot_ represents the total number of individuals in the population, l_X_ represents the prevalence of stage X in the population (where X is I for HPV infected without AIN, L for LGAIN and H for HGAIN, and C for anal cancer), r_X_ represents the monthly clearance probability from state X to immune, and d represents the monthly probability of dying. Equation 1 can be simplified to:

$l_{tot}r_{tot}=l_{I}r_{I}+l_{L}r_{L}+l_{H}r_{H}$ (2)

Equation 2 reflects that individuals who have LGAIN and HGAIN are still actively infected with HPV, and thus the rate of clearance of any HPV state relates to r_tot_.

In addition, a simple sum of the proportion of individuals with HPV in any state results in the following equation:

$l_{tot}=l_{I}+l_{L}+l_{H}$ (3)

Second, at steady state, the number of individuals in either the LGAIN or HGAIN must stay constant, such that the number of enter through progression must eventually leave through regression, progression, or death. This results in the following two equations:

$p_{L}l_{I}={\left( r_{L}+p_{H}+d \right)l}_{L}$ (4)

$p_{H}l_{L}={\left( r_{H}+p_{C}+d \right)l}_{H}$ (5)

Here, p_X_ represents the monthly progression probability to a more severe state X.

The values for the progression variables were derived in the previous section. The clearance rate (r_tot_) from any HPV state, as well of the death rate (d) are available. This results in seven variables remaining (l_tot_, l_l_, l_L_, l_H_, r_I_, r_L_, r_H_), but only four equations (Eqs 2-5).

Since the goal is to derive the clearance rates in relation to r_tot_ for use in the anal cancer model, we assume that the ratio of the infected only to LGAIN prevalences is fixed (Equation 6), and the ratio of LGAIN to HGAIN prevalences is also fixed (Equation 7), as r_tot_ is varied through scenario analysis.

| $\alpha=\frac{l_{I}}{l_{L}}$ | (6) |
| --- | --- |
| $\beta=\frac{l_{L}}{l_{H}}$ | (7) |

Thus, using equation 6 and 7, equations 2 and 3 can be combined into the following equation:

| $r_{tot}\left( 1+\frac{1}{\alpha}+\frac{1}{\alpha\beta} \right)=r_{I}+\frac{r_{L}}{\alpha}+\frac{r_{H}}{\alpha\beta}$ | (8) |
| --- | --- |

Also, equations 4 and 5 can be combined into the following equation:

| $\frac{p_{L}}{p_{H}}\alpha=\frac{\left( r_{L}+p_{H}+d \right)}{\left( r_{H}+p_{C}+d \right)}\beta$ | (9) |
| --- | --- |

Finally, we assume that r_I_ is proportionate to r_tot_ – specifically, r_I_ = 0.8 × r_tot_. This parameter fixes the proportion of clearances coming from the Infected state vs LGAIN or HGAIN states. Note that the prevalence of Infected over prevalence of any HPV state is 0.690 for HIV negative and 0.634 for HIV positive (see next section), so this parameter should at least be larger than either of these values.

Lastly, equation 8 and 9 can be calculated to solve r_L_ and r_H_, as a function of r_tot_, with the following values for the prevalences and death rate.

*Prevalences*

To calculate anal cancer clearance rates, we needed HPV prevalence from a general MSM population. We used HPV prevalence reported in a systematic review conducted in 2012 by Machalek [24]. For prevalence of any HPV state, since this anal cancer model is mixed for HPV-16/18, average of the extremes of assuming that HPV-16 and HPV-18 infections all occurred in the same individuals or in different individuals was used.

To determine what percentages of LGAIN and HGAIN found in Machalek 2012 were associated with HPV-16/18, values from De Vuyst et al. [23] were used, which found that 91.5% of AIN1 and 93.9% of AIN2/3 were associated with HPV. To evaluate the percentage of those associated with HPV-16/18, the average of the extremes of assuming that HPV-16 and HPV-18 infections all occurred in the same individuals or in different individuals was used.

*Mortality rate*

The death rate of a 50-year-old (0.000251/month) was used for HIV negatives [5]. The death rate of HIV positives was 2.18 time greater [6] (see Natural Mortality section).

*Rate of progression from HGAIN to anal cancer (HGAIN → cancer)*

We fitted the following formula for the transition probability from HGAIN to cancer (using estimated cancer incidence in HIV-negative and HIV-positive MSM) to ONS 2008-2012 anal cancer incidence data, where age is in years:

ω = µ_1_·exp(µ_2_·age)

This produced the following parameter values:

**Table.** Parameters for the transition probabilities from HGAIN to cancer by HPV type and HIV status.

| **HPV Type** | **HIV-negative** | **HIV-positive** |
| --- | --- | --- |
| *HPV-16* |  |  |
| µ_1_ | 7.6 × 10^-6^ | 2.2 × 10^-5^ |
| µ_2_ | 0.070 | 0.057 |
| *HPV-18* |  |  |
| µ_1_ | 3.4 × 10^-6^ | 7.3 × 10^-6^ |
| µ_2_ | 0.068 | 0.065 |

The resulting equations are shown in the figure below:

**Figure.** Monthly probability of transition from HGAIN to cancer for HPV-16/18 by HIV status. Note that the y-axis is a log scale.

# A4. Parameters affecting disease burden and costs

## A4.1. Quality of life

### A4.1.1. Anogenital warts

Quality of life loss (disutility) experienced by men with anogenital warts was measured in the Qoligen study [40]. This study surveyed 494 men receiving anogenital warts treatment in eight UK GUM clinics using the EuroQoL EQ-5D questionnaire, and combined them with estimates of the duration of anogenital warts episodes from a case notes review of 213 other men. Disutility weights in all men regardless of MSM status were used, since there were no statistically significant differences in EQ-5D values by MSM status (Sarah Woodhall, personal communication). However, while the time to attendance at genitourinary clinic after noticing anogenital warts were the same, the duration of each episode of care, treatment and recovery time were different for MSM. Hence these specific values were used to calculate the final QALY values, estimated at 0.022 (range 0.005-0.051) QALY loss per anogenital warts episode. We accounted for recurrence of anogenital warts by multiplying the number of first-time anogenital warts episodes by 1.156 for HIV-negative MSM and 1.204 for HIV-positive MSM, from GUMCAD.

### A4.1.2. Cancer: combining utility values during treatment, recovery, and post-cancer survival

Disutilities associated with cancer outcomes were estimated as 1 minus the utility weight measured in quality of life studies (rather than the usual practice of using the difference between the utility weight of people with a disease and the average utility weight in the general population). This is because the studies assumed a utility weight of 1 for full health, and did not measure utilities in a UK population (so we did not use UK population norms for the average utility weight in the general population). QALY losses were calculated by combining the utility decrement during (1) treatment phase (2) recovery phase (3) survival post-cancer. The treatment phase was estimated to last 1.4 months [41]. Patients with anal and penile cancers were assumed to recover linearly over 18 months after treatment [41]. During the 18-month recovery period, patients with oropharyngeal/oral cavity/laryngeal cancers have a disutility of 0.25 (normal mean, SD 0.02) [42].

## A4.2. Survival estimates and utility values post-cancer

We assumed that following cancer treatment, patients had reduced survival and health-related quality of life compared to the general population. No such assumptions were made for anogenital warts. Relative cancer survival data for men were obtained from the ONS [43,44] and other published data [45–47]. This was applied to mortality in the general population to obtain absolute cancer survival in HIV-negative MSM.

The assumed treatment and recovery period were 1.4 months and 18 months, respectively, for anal/penile and oropharyngeal/oral cavity/laryngeal cancers. These periods were deducted to ensure that utility during treatment and recovery were not double counted, because ONS statistics and the related published data from ONS represented survival post-cancer diagnosis, not post-treatment and recovery. No deductions were made if survival is less than the treatment and recovery period.

### A4.2.1. Anal cancer survival rates

We combined information from two papers [47,48] to estimate anal cancer survival.

The first paper by Jeffreys et al. [47] reported the survival rate among patients with ano-rectal cancer diagnosed between 1986 and 2001, and reported that five-year relative survival is lower in men (48%), compared with women (51%). Additional analysis on age-stratified one-year anal cancer survival rates in men diagnosed between 1991 and 1999 was provided by the authors (Mona Jeffreys, personal communication).

**Table.** Relative one-year anal cancer survival in men diagnosed between 1991 and 1999.

| **Age group** | **Proportion of patients (%)** | **Relative survival (%)** | **95% CI (lower)** | **95% CI (upper)** |
| --- | --- | --- | --- | --- |
| **Overall** |  | **73.3** | **71.4** | **75.2** |
| 15-59 | 29.4 | 83.2 | 80.2 | 85.7 |
| 60-69 | 28.9 | 76.0 | 72.5 | 79.1 |
| 70-79 | 27.6 | 64.9 | 60.8 | 68.6 |
| 80-89 | 12.5 | 54.4 | 47.7 | 60.6 |
| 90-99 | 1.6 | 64.4 | 39.6 | 81.1 |

The second paper by James et al. [48] reported survival rates in a trial of 940 UK anal cancer patients who were assigned to different treatment regimens (Mitomycin or Cisplatin, with or without maintenance therapy) and followed for a median of 5.1 years. The trial period was between June 2001 and December 2008. For our analysis, we used the reported overall survival among the 246 patients who were given Mitomycin with no maintenance therapy [48], which is the current standard treatment in the UK. One- and five-year survival was 91% and 81%, respectively. However, this is for men and women combined, and for all ages.

Hence, we used age distribution of ano-rectal cancer in men, ratio of worse survival in men (compared to women), and, for the one-year survival analysis, their one-year odds of survival in Jeffreys et al. [47] and combined these information with the overall survival estimates from the more recent study in anal cancer patients i.e. James et al. [48].

For one-year survival estimates, we first calculated the odds of survival, based on data from Jeffreys et al. [47], using their age-group distribution. For example, for age group 60-69, the relative survival was 75.99%, so the odds of survival is (75.99)/(100-75.99) = 3.1649. We then weighted these odds by the proportion of men falling into this age group, compared to all ages. This gives a weighted contribution to the overall odds of survival of 3.1649 x 28.91% = 0.8733. We did the same calculations for all other age-groups and arrived at a total weighted odds of survival of 2.5492, which is the weighted average odds of survival for all men. Hence, the odds of survival compared to overall weighted average odds of survival for men with anal cancer in the age-group 60-69 was 3.1649/2.5492 = 1.2415.

Next, since the one-year survival in men and women reported in James et al. [48] was 91%, we estimated that one-year survival in men would be lower at 91% x 48%/51% = 85.54%, giving an odds of survival of (85.54)/(100-85.54) = 5.9156. This was used to calculate the uprated odds of one-year survival compared to weighted average survival. For the 60-69 age group, this was 5.9156 x 1.2415 = 7.34. This gives an uprated survival estimate of (7.34)/(1+7.34) = 88.02%.

We used a similar approach to estimate five-year survival estimates. However, we did not have male-only anal cancer survival estimates. Hence, we used five-year relative ano-rectal survival data for both men and women reported in Table 2 of Jeffreys et al. [47] and adjusted them down by the same ratio of 48%/51% = 94% used above, to give estimates of survival in men only. We then calculated the odds of survival, weighted average survival, and the odds of survival compared to this weighted average survival. Finally, we used the five-year survival estimates from James et al. [48] of 81% and adjusted it down to 81% x 48%/51% = 76.14% for a men-only estimate, we calculated the uprated odds of survival, before calculating five-year survival estimate.

The final survival estimates we used are given below:

Table. Estimated one-year and five-year net survival for anal cancer (%), with 95% confidence intervals (CI), for men (15–99 years) diagnosed between 2001–2008 in England.

| **Age group** | **One-year survival** | | | **Five-year survival** | | |
| --- | --- | --- | --- | --- | --- | --- |
|  | **Relative survival (%)** | **95% CI** | | **Relative survival (%)** | **95% CI** | |
| 15-59 | 90.5 | 88.7 | 92.1 | 79.9 | 79.1 | 80.7 |
| 60-69 | 86.0 | 83.6 | 88.0 | 77.9 | 77.1 | 78.7 |
| 70-79 | 78.1 | 75.0 | 80.9 | 73.6 | 72.7 | 74.5 |
| 80-89 | 69.8 | 63.9 | 74.9 | 63.3 | 61.2 | 65.3 |
| 90-99 | 77.8 | 56.0 | 89.3 | 42.1 | 32.6 | 51.1 |

We could not find HPV-specific anal cancer survival rates estimates. Nevertheless, given that about 84% of anal cancers are HPV-related, anal cancer survival rates should be largely representative of HPV-specific anal cancer survival rates.

### A.4.2.2. Oropharyngeal cancer survival rates

Survival outcomes for people with HPV-positive oropharyngeal cancer (including oropharynx and tonsil) appear to be better than those with oropharyngeal cancer not linked to HPV infection [45,49–51]. There were no age-specific survival rates for the UK available from published literature. Hence, we used ONS survival estimates for oropharyngeal cancers diagnosed in 1996-1999 in England and Wales [43] and adjusted the values based on US data published in Ang et al. [51]. Ang et al.’s data were used to adjust for better survival estimates in HPV-related oropharyngeal cancer due to the large study sample size although we recognise that there are other estimates available.

The following formulae were applied:

Survival rate in HPV-positive oropharyngeal cancer = (s1/s2 × OS) / (s1/s2 × p1 + p2)

where s1 and s2 are, respectively, the survival rate in HPV-positive and HPV-negative oropharyngeal cancers in Ang et al. [51], OS is the unadjusted survival rates in ONS data, and p1 and p2 are, respectively, the proportion of oropharyngeal cancers in Ang et al. [51] that are HPV-positive and HPV-negative.

For one-year survival rates, we have from Figure 1A of Ang et al. [51], s1=94, s2=76, p1=206/323, p2=117/323. For five-year survival rates, we have s1=77, s2=49, p1=73/95, p2=22/95. For OS, the original ONS survival rates are as follows:

Table. One- and five-year survival of men diagnosed with oropharyngeal cancer in 1996-99 in England and Wales (adapted from ONS [43]).

| **Age group** | **One-year survival** | | | **Five-year survival** | | |
| --- | --- | --- | --- | --- | --- | --- |
|  | **Relative survival (%)** | **95% CI** | | **Relative survival (%)** | **95% CI** | |
| 15-49 | 82 | 78 | 86 | 59 | 53 | 65 |
| 50-69 | 71 | 68 | 74 | 42 | 38 | 46 |
| 70-99 | 53 | 47 | 59 | 26 | 18 | 35 |

When adjusted for higher survival rates using Ang et al. [51], these estimates become:

**Table.** One-year and five-year relative HPV-specific survival for male patients diagnosed in 1996-99 with oropharyngeal cancer.

| **Age group** | **One-year survival** | | | **Five-year survival** | | |
| --- | --- | --- | --- | --- | --- | --- |
|  | **Relative survival (%)** | **95% CI** | | **Relative survival (%)** | **95% CI** | |
| 15-49 | 88.1 | 83.8 | 92.4 | 64.4 | 57.9 | 71.0 |
| 50-69 | 76.3 | 73.1 | 79.5 | 45.9 | 41.5 | 50.2 |
| 70-99 | 57.0 | 50.5 | 63.4 | 28.4 | 19.7 | 38.2 |

### A4.2.3. Penile cancer survival rates

Data corresponding to Figure 6 of a recently published study [46] were obtained from one of the co-authors (Ruoran Li, personal communication on 15 July 2014) for one-year and five-year survival estimates. These were for patients in England diagnosed in 2006-2010 and followed-up to 2011.

Table. One-year and five-year survival in penile cancer patients diagnosed 2006-2010 and followed up to 2011, England ([46] and data from Ruoran Li, personal communication)

| **Age group** | **One-year survival** | | | **Five-year survival** | | |
| --- | --- | --- | --- | --- | --- | --- |
|  | **Relative survival (%)** | **95% CI** | | **Relative survival (%)** | **95% CI** | |
| 15-49 | 93.9 | 90.3 | 96.2 | 77.2 | 70.3 | 82.6 |
| 50-59 | 91.8 | 88.2 | 94.3 | 76.5 | 70.0 | 82.0 |
| 60-69 | 89.5 | 86.4 | 92.0 | 74.1 | 68.3 | 79.0 |
| 70-79 | 84.8 | 80.8 | 88.0 | 69.8 | 61.3 | 76.9 |
| 80-99 | 77.0 | 71.0 | 82.0 | 53.0 | 38.7 | 65.4 |

Again, we could not find HPV-specific estimates. Survival for penile cancer is fairly high but we do not know if penile cancers caused by HPV have better or worse survival rates.

### A4.2.4. Oral cavity and laryngeal cancer survival rates

For oral cavity cancers, we used ONS survival data from men diagnosed between 1996 and 1999 [43]. Survival data for laryngeal cancer was obtained from ONS data from men diagnosed between 2007 and 2011 ([52]).

Table. One-year and five-year survival in oral cavity cancer patients diagnosed 1996-1999 [43]

| **Age group** | **One-year survival** | | | **Five-year survival** | | |
| --- | --- | --- | --- | --- | --- | --- |
|  | **Relative survival (%)** | **95% CI** | | **Relative survival (%)** | **95% CI** | |
| 15-49 | 84 | 80 | 88 | 59 | 52 | 64 |
| 50-69 | 78 | 75 | 80 | 50 | 47 | 54 |
| 70-99 | 64 | 60 | 68 | 40 | 33 | 46 |

 Table. One-year and five-year survival in laryngeal cancer patients (adults aged 15-99 years) diagnosed during 2007-2011, England [52]

| **Age group** | **One-year survival** | | | **Five-year survival** | | |
| --- | --- | --- | --- | --- | --- | --- |
|  | **Relative survival (%)** | **95% CI** | | **Relative survival (%)** | **95% CI** | |
| 15–49 | 90 | 87 | 93 | 76 | 72 | 81 |
| 50–59 | 89 | 87 | 90 | 71 | 68 | 74 |
| 60–69 | 86 | 85 | 88 | 66 | 63 | 68 |
| 70–79 | 83 | 81 | 84 | 64 | 60 | 68 |
| 80–99 | 75 | 72 | 78 | 56 | 47 | 64 |

## A4.3. Utility values for the post-cancer period

Upon recovery from anal or oropharyngeal/oral cavity/laryngeal cancer, disutility was assumed to take triangular distribution with mode 0.0305 (min 0, max 0.061) [41]. This was based on quality of life in women with cervical cancer, taken as a proxy. Following recovery from penile cancer, disutility was estimated to be 0.051 using estimates from the Institute of Medicine [53], assuming that 90% of penile cancers were squamous cell penile cancer or carcinoma in-situ, 5% locally invasive at diagnosis, 5% advanced at diagnosis [54]. This was taken as a mean with its 95% CI as 25% lower and 25% higher than this estimate.

**Figures.** Utility values applied to different phases of cancer.

(a) Anal and penile cancers

| Survival post-cancer minus time from cancer diagnosis to the end of recovery  Recovery after treatment over 18 months  0  1  Death  Cancer diagnosis and treatment  Full health  Time  Utility |
| --- |

(b) Oropharyngeal/oral cavity/laryngeal cancers

| Survival post-cancer minus time from cancer diagnosis to the end of recovery  Recovery after treatment over 18 months  0  1  Death  Cancer diagnosis and treatment  Full health  Time  Utility |
| --- |

## A4.4. Costs

### A.4.4.1. Anogenital wart (AGW) treatment costs

We applied treatment costs per AGW episode (excluding STI screening costs) in men, regardless of MSM or HIV status, as reported in the QOLIGEN study in eight English sexual health clinics (19). The same cost per episode was applied to first and recurrent AGW cases, since the estimated (and inflated) values of £105.54 (95% CI £94.66 – £116.42) include both first and recurrent episodes treated in GUM clinics, general practitioner offices and hospitals.

### A.4.4.2. Cancer treatment costs

We used a recent estimate of the treatment cost for anal cancer in England, calculated using Hospital Episode Statistics data and Markov model. The average cost of £16,281 (range £14,143 to £22,884) in 2011 GBP is higher than the estimate used in our previous economic evaluation of female HPV vaccination of £13,050 in 2009/10 values [41], the latter of which was calculated relative to the treatment cost of cervical cancer. We adjusted the values to 2013/14 prices of £17,093 (range £14,848 to £24,025).

Treatment costs for head and neck cancers is an area with high uncertainty [55]. We have therefore used the cost estimate used in our previous cost-effectiveness analysis of female HPV vaccination, and applied the same cost for treating oropharyngeal cancers as for oral cavity cancers [41]. Therefore, the cost of managing oropharyngeal cancer, which was calculated relative to the cost of cervical cancer, was £15,000 (2008-9 prices; £16,223 in 2013/14 prices).

For penile cancers, we used the cost estimates in a recent retrospective study [56], which reported that the estimated cost of treating this cancer in secondary care of £7,421 (range £5,930 to £10,104) in 2010/11 values (using the scenario with no inflation or correction for regional market forces). These were adjusted to 2013/14 prices of £7,791 (range £6,226 to £10,608). The authors concluded that these costs were similar to the treatment cost for other urological cancers. This estimate is lower than that used in our previous cost-effectiveness analysis of female HPV vaccination in 2011 [41].

# A5. Summary tables of parameters

Parameters not already shown in Table 1 of the main text are shown below.

Table. Monthly transition probabilities for anal cancer progression.

| **Parameter** | **Transition** | **Value in HIV-negatives** | **Reference** | **Value in HIV-positives** | **Reference** |
| --- | --- | --- | --- | --- | --- |
| σ (p_L_) | Infected → LGAIN | 0.030 | [38] | 0.056 | [38][57] |
| χ (p_H_) | LGAIN → HGAIN | 0.0082 | [57] | 0.0127 | [58] |
| p_c_ | HGAIN → Cancer | 0.00002 | [24] | 0.00022 | [24] |
| ω | HGAIN → Cancer | Age-dependent exponential | Fitted | Age-dependent exponential | Fitted |
| *r* (r_I_) | Infected → Immune | 0.061-0.191 | Calculated | 0.010-0.030 | Calculated |
| ρ (r_L_) | LGAIN → Immune | 0.033-0.116 | Calculated | 0.005-0.016 | Calculated |
| φ (r_H_) | HGAIN → Immune | 0.005-0.015 | Calculated | 0.004-0.007 | Calculated |

Table. Prevalences used in calculation of clearance probabilities.

| **Parameter** | **HPV-16/18-related Prevalence of** | **HIV-negative** | **Reference** | **HIV-positive** | **Reference** |
| --- | --- | --- | --- | --- | --- |
| l_tot_ | Any HPV state | 0.150 | [24] | 0.447 | [24] |
| I_I_ | Infected | 0.103 | Calculated | 0.284 | Calculated |
| I_L_ | LGAIN | 0.029 | [24][23] | 0.120 | [24][23] |
| l_H_ | HGAIN | 0.017 | [24][23] | 0.043 | [24][23] |

Table. Calculated monthly clearance probabilities as a function of the duration of infection.

|  | **HIV-negative** | | | | | **HIV-positive** | | | | |
| --- | --- | --- | --- | --- | --- | --- | --- | --- | --- | --- |
| Duration of Infection (months) | 5.9 | 7.1 | 9.1 | 12.5 | 20.0 | 41.7 | 50.0 | 62.5 | 83.3 | 125.0 |
| r from any HPV state | 0.156 | 0.131 | 0.104 | 0.077 | 0.049 | 0.024 | 0.020 | 0.016 | 0.012 | 0.008 |
| r_I_ | 0.191 | 0.161 | 0.128 | 0.095 | 0.061 | 0.030 | 0.025 | 0.020 | 0.015 | 0.010 |
| r_L_ | 0.116 | 0.095 | 0.074 | 0.053 | 0.033 | 0.016 | 0.013 | 0.011 | 0.008 | 0.005 |
| r_H_ | 0.015 | 0.013 | 0.010 | 0.007 | 0.005 | 0.006 | 0.006 | 0.005 | 0.004 | 0.004 |

**Table.** Economic parameters and their distributions. All values have been converted to 2013/14 GBP using HCHS Index [59].

| **Parameter** | **Distribution** | **Base-case** | **Range** | **Reference** |
| --- | --- | --- | --- | --- |
| ***Costs*** |  |  |  |  |
| Cancer treatment |  |  |  |  |
| Anal cancer | Gamma | £17,093 | £14,848-£24,025 | [41] |
| Oropharyngeal cancer | Gamma | £16,223 | £14,114-£17,845 | [41] |
| Penile cancer | Gamma | £7,791 | £6,226-£10,608 | [56] |
| Oral cavity cancer | Gamma | £16,223 | £14,114-£17,845 | [41] |
| Laryngeal cancer | Gamma | £16,223 | £14,114-£17,845 | [41] |
| Warts treatment | Gamma | £106 | £95-£116 | [40] |
| Vaccination/dose |  |  |  |  |
| Administration | Uniform | £10.00 |  | Assumption, [59] |
| Gardasil | Uniform | £86.50 |  | [60] |
|  |  |  |  |  |
| ***QALY losses*** |  |  |  |  |
| Cancer treatment duration | Log-normal | 42.3 days | 1.03-163 days | [41] |
| Treatment for |  |  |  |  |
| Anal cancer | Beta | 0.43 | 0.38-0.48 | [61] |
| Oropharyngeal cancer | Beta | 0.42 | 0.37-0.47 | [61] |
| Penile cancer | Beta | 0.21 | 0.16-0.26 | [61] |
| Oral cavity cancer | Beta | 0.42 | 0.37-0.47 | [61] |
| Laryngeal cancer | Beta | 0.42 | 0.37-0.47 | [61] |
| Recovery from |  |  |  |  |
| Anal cancer | Linear recovery over 18 months | | | [41] |
| Oropharyngeal cancer | Beta | 0.25 | 0.21-0.29 | [42] |
| Penile cancer | Linear recovery over 18 months | | | [41] |
| Oral cavity cancer | Beta | 0.25 | 0.21-0.29 | [42] |
| Laryngeal cancer | Beta | 0.25 | 0.21-0.29 | [42] |
| Survival from |  |  |  |  |
| Anal cancer | Triangular | 0.031 | 0-0.061 | [41] |
| Oropharyngeal cancer | Triangular | 0.031 | 0-0.061 | [41] |
| Penile cancer | Triangular | 0.051 | 0.038-0.064 | [53,54] |
| Oral cavity cancer | Triangular | 0.031 | 0-0.061 | [41] |
| Laryngeal cancer | Triangular | 0.031 | 0-0.061 | [41] |
| Episode of warts | Beta | 0.022 | 0.005-0.051 | [40] |

# A6. Model equations

*HPV-16/18*

Not sexually active:

$${NA}_{0,0,i+1,l,0}=\left( 1-d_{0,i}-a_{i,l} \right){NA}_{0,0,i,l,0}$$

Sexually active, HIV negative, not attending clinics, not vaccinated:

$$S_{0,0,i+1,l,0}=\left( 1-d_{0,i}-\lambda_{HIV,i,l}-c_{i}-\lambda_{HPV,0,yi,l} \right)S_{0,0,i,l,0}+\mu_{0}R_{0,0,i,l,0}+a_{i,l}{NA}_{0,0,i,l,0}$$

$$I_{0,0,i+1,l,0}=\left( 1-d_{0,i}-\lambda_{HIV,i,l}-c_{i}-r_{0}-\sigma_{0} \right)I_{0,0,i,l,0}+\lambda_{HPV,0,yi,l}S_{0,0,i,l,0}$$

$${LAIN}_{0,0,i+1,l,0}=\left( 1-d_{0,i}-\lambda_{HIV,i,l}-c_{i}-\chi_{0}-\rho_{0} \right){LAIN}_{0,0,i,l,0}+\sigma_{0}I_{0,0,i,l,0}$$

$${HAIN}_{0,0,i+1,l,0}=\left( 1-d_{0,i}-\lambda_{HIV,i,l}-c_{i}-\omega_{0,i}-\phi_{0} \right){HAIN}_{0,0,i,l,0}+\chi_{0}{LAIN}_{0,0,i,l,0}$$

$${Canc}_{0,0,i+1,l,0}=\left( 1-d_{0,i}-\lambda_{HIV,i,l}-c_{i} \right){Canc}_{0,0,i,l,0}+\omega_{0,i}{HAIN}_{0,0,i,l,0}$$

$$R_{0,0,i+1,l,0}=\left( 1-d_{0,i}-\lambda_{HIV,i,l}-c_{i}-\mu_{0} \right)R_{0,0,i,l,0}+r_{0}I_{0,0,i,l,0}+\rho_{0}{{LAIN}_{0,0,i,l,0}+\phi}_{0}{HAIN}_{0,0,i,l,0}$$

Sexually active, HIV negative, attending clinics, not vaccinated:

$$S_{0,1,i+1,l,0}=\left( 1-d_{0,i}-\lambda_{HIV,i,l}-v_{0,i}-\lambda_{HPV,0,yi,l} \right)S_{0,1,i,l,0}+\mu_{0}R_{0,1,i,l,0}+c_{i}S_{0,0,i,l,0}$$

$$I_{0,1,i+1,l,0}=\left( 1-d_{0,i}-\lambda_{HIV,i,l}-v_{0,i}-r_{0}-\sigma_{0} \right)I_{0,1,i,l,0}+\lambda_{HPV,0,yi,l}S_{0,1,i,l,0}+c_{i}I_{0,1,i,l,0}$$

$${LAIN}_{0,1,i+1,l,0}=\left( 1-d_{0,i}-\lambda_{HIV,i,l}-v_{0,i}-\chi_{0}-\rho_{0} \right){LAIN}_{0,1,i,l,0}+\sigma_{0}I_{0,1,i,l,0}+c_{i}{LAIN}_{0,0,i,l,0}$$

$${HAIN}_{0,1,i+1,l,0}=\left( 1-d_{0,i}-\lambda_{HIV,i,l}-v_{0,i}-\omega_{0,i}-\phi_{0} \right){HAIN}_{0,1,i,l,0}+\chi_{0}{LAIN}_{0,1,i,l,0}+c_{i}{HAIN}_{0,0,i,l,0}$$

$${Canc}_{0,1,i+1,l,0}=\left( 1-d_{0,i}-\lambda_{HIV,i,l}-v_{0,i} \right){Canc}_{0,1,i,l,0}+\omega_{0,i}{HAIN}_{0,1,i,l,0}+c_{i}{Canc}_{0,0,i,l,0}$$

$$R_{0,1,i+1,l,0}=\left( 1-d_{0,i}-\lambda_{HIV,i,l}-v_{0,i}-\mu_{0} \right)R_{0,1,i,l,0}+r_{0}I_{0,1,i,l,0}+c_{i}R_{0,1,i,l,0}+\rho_{0}{{LAIN}_{0,1,i,l,0}+\phi}_{0}{HAIN}_{0,1,i,l,0}$$

Sexually active, HIV positive, attending clinics, not vaccinated:

$$S_{1,1,i+1,l,0}=\left( 1-d_{1,i}-v_{1,i}-\lambda_{HPV,1,yi,l} \right)S_{1,1,i,l,0}+\mu_{1}R_{1,1,i,l,0}+ \lambda_{HIV,i,l}(S_{0,0,i,l,0}+S_{0,1,i,l,0})$$

$$I_{1,1,i+1,l,0}=\left( 1-d_{1,i}-v_{1,i}-r_{1}-\sigma_{1} \right)I_{1,1,i,l,0}+\lambda_{HPV,1,yi,l}S_{1,1,i,l,0}+ \lambda_{HIV,i,l}(I_{0,0,i,l,0}+I_{0,1,i,l,0})$$

$${LAIN}_{1,1,i+1,l,0}=\left( 1-d_{1,i}-\lambda_{HIV,i}-v_{1,i}-\chi_{1}-\rho_{1} \right){LAIN}_{1,1,i,l,0}+\sigma_{1}I_{1,1,i,l,0}+\lambda_{HIV,i,l}({LAIN}_{0,0,i,l,0}+{LAIN}_{0,1,i,l,0})$$

$${HAIN}_{1,1,i+1,l,0}=\left( 1-d_{1,i}-\lambda_{HIV,i}-v_{1,i}-\omega_{1,i}-\phi_{1} \right){HAIN}_{1,1,i,l,0}+\chi_{1}{LAIN}_{1,1,i,l,0}+\lambda_{HIV,i,l}({HAIN}_{0,0,i,l,0}+{HAIN}_{0,1,i,l,0})$$

$${Canc}_{1,1,i+1,l,0}=\left( 1-d_{1,i}-\lambda_{HIV,i}-v_{1,i} \right){Canc}_{1,1,i,l,0}+\omega_{1,i}{HAIN}_{1,1,i,l,0}+\lambda_{HIV,i,l}({Canc}_{0,0,i,l,0}+{Canc}_{0,1,i,l,0})$$

$$R_{1,1,i+1,l,0}=\left( 1-d_{1,i}-v_{1,i}-\mu_{1} \right)R_{1,1,i,l,0}+r_{1}I_{1,1,i,l,0}+\rho_{1}{{LAIN}_{1,1,i,l,0}+\phi}_{1}{HAIN}_{1,1,i,l,0}+ \lambda_{HIV,i,l}(R_{0,0,i,l,0}+R_{0,1,i,l,0})$$

Sexually active, HIV negative, attending clinics, vaccinated:

$$S_{0,1,i+1,l,1}=\left( 1-d_{0,i}-\lambda_{HIV,i,l}-w_{0,i} \right)S_{0,1,i,l,1}+{v_{0,i}{\gamma S}_{0,1,i,l,0}+\mu}_{0}R_{0,1,i,l,1}$$

$$I_{0,1,i+1,l,1}=\left( 1-d_{0,i}-\lambda_{HIV,i,l}-w_{0,i}-r_{0}-\sigma_{0} \right)I_{0,1,i,l,1}+v_{0,i}{\gamma I}_{0,1,i,l,0}$$

$${LAIN}_{0,1,i+1,l,1}=\left( 1-d_{0,i}-\lambda_{HIV,i,l}-w_{0,i}-\chi_{0}-\rho_{0} \right){LAIN}_{0,1,i,l,1}+\sigma_{0}I_{0,1,i,l,1}+v_{0,i}\gamma{LAIN}_{0,1,i,l,0}$$

$${HAIN}_{0,1,i+1,l,1}=\left( 1-d_{0,i}-\lambda_{HIV,i,l}-w_{0,i}-\omega_{0,i}-\phi_{0} \right){HAIN}_{0,1,i,l,1}+\chi_{0}{LAIN}_{0,1,i,l,1}+v_{0,i}\gamma{HAIN}_{0,1,i,l,0}$$

$${Canc}_{0,1,i+1,l,1}=\left( 1-d_{0,i}-\lambda_{HIV,i,l}-w_{0,i} \right){Canc}_{0,1,i,l,1}+\omega_{0,i}{HAIN}_{0,1,i,l,1}+v_{0,i}\gamma{Canc}_{0,1,i,l,0}$$

$$R_{0,1,i+1,l,1}=\left( 1-d_{0,i}-\lambda_{HIV,i,l}-w_{0,i}-\mu_{0} \right)R_{0,1,i,l,1}+v_{0,i}{\gamma R}_{0,1,i,l,0}+r_{0}I_{0,1,i,l,0}+\rho_{0}{{LAIN}_{0,1,i,l,1}+\phi}_{0}{HAIN}_{0,1,i,l,1}$$

Sexually active, HIV negative, attending clinics, vaccine failure:

$$S_{0,1,i+1,l,2}=\left( 1-d_{0,i}-\lambda_{HIV,i,l}-\lambda_{HPV,0,yi,l} \right)S_{0,1,i,l,2}+{v_{0,i}{\left( 1-\gamma\right)S}_{0,1,i,l,0}+\mu}_{0}R_{0,1,i,l,1}+w_{0,i}S_{0,1,i,l,1}$$

$$I_{0,1,i+1,l,2}=\left( 1-d_{0,i}-\lambda_{HIV,i,l}-r_{0}-\sigma_{0} \right)I_{0,1,i,l,2}+v_{0,i}{(1-\gamma)I}_{0,1,i,l,0}+\lambda_{HPV,0,yi,l}S_{0,1,i,l,2}+w_{0,i}I_{0,1,i,l,1}$$

$${LAIN}_{0,1,i+1,l,2}=\left( 1-d_{0,i}-\lambda_{HIV,i,l}-\chi_{0}-\rho_{0} \right){LAIN}_{0,1,i,l,2}+\sigma_{0}I_{0,1,i,l,2}+v_{0,i}(1-\gamma){LAIN}_{0,1,i,l,0}+w_{0,i}{LAIN}_{0,1,i,l,1}$$

$${HAIN}_{0,1,i+1,l,2}=\left( 1-d_{0,i}-\lambda_{HIV,i,l}-\omega_{0,i}-\phi_{0} \right){HAIN}_{0,1,i,l,2}+\chi_{0}{LAIN}_{0,1,i,l,2}+v_{0,i}(1-\gamma){HAIN}_{0,1,i,l,0}{+w}_{0,i}{HAIN}_{0,1,i,l,1}$$

$${Canc}_{0,1,i+1,l,2}=\left( 1-d_{0,i}-\lambda_{HIV,i,l} \right){Canc}_{0,1,i,l,2}+\omega_{0,i}{HAIN}_{0,1,i,l,2}+v_{0,i}(1-\gamma){Canc}_{0,1,i,l,0}{+w}_{0,i}{Canc}_{0,1,i,l,1}$$

$$R_{0,1,i+1,l,2}=\left( 1-d_{0,i}-\lambda_{HIV,i,l}-\mu_{0} \right)R_{0,1,i,l,2}+v_{0,i}(1-{\gamma)R}_{0,1,i,l,0}+r_{0}I_{0,1,i,l,2}+\rho_{0}{{LAIN}_{0,1,i,l,2}+\phi}_{0}{HAIN}_{0,1,i,l,2}+w_{0,i}R_{0,1,i,l,1}$$

Sexually active, HIV positive, attending clinics, immunised:

$$S_{1,1,i+1,l,1}=\left( 1-d_{1,i}-w_{0,i} \right)S_{1,1,i,l,1}+{v_{1,i}{\gamma S}_{1,1,i+1,l,0}+\mu}_{1}R_{1,1,i,l,1}+\lambda_{HIV,i,l}S_{0,1,i,l,1}$$

$$I_{1,1,i+1,l,1}=\left( 1-d_{1,i}-w_{1,i}-r_{1}-\sigma_{1} \right)I_{1,1,i,l,1}+v_{1,i}{\gamma I}_{1,1,i+1,l,0}+\lambda_{HIV,i,l}I_{0,1,i,l,1}$$

$${LAIN}_{1,1,i+1,l,1}=\left( 1-d_{1,i}-w_{1,i}-\chi_{1}-\rho_{1} \right){LAIN}_{1,1,i,l,1}+\sigma_{1}I_{1,1,i,l,1}+v_{1,i}\gamma{LAIN}_{1,1,i,l,0}+\lambda_{HIV,i,l}{LAIN}_{0,1,i,l,1}$$

$${HAIN}_{1,1,i+1,l,1}=\left( 1-d_{1,i}-w_{1,i}-\omega_{1,i}-\phi_{1} \right){HAIN}_{1,1,i,l,1}+\chi_{1}{LAIN}_{1,1,i,l,1}+v_{1,i}\gamma{HAIN}_{1,1,i,l,0}+\lambda_{HIV,i,l}{HAIN}_{0,1,i,l,1}$$

$${Canc}_{1,1,i+1,l,1}=\left( 1-d_{1,i}-w_{1,i} \right){Canc}_{1,1,i,l,1}+\omega_{1,i}{HAIN}_{1,1,i,l,1}+v_{1,i}\gamma{Canc}_{1,1,i,l,0}+\lambda_{HIV,i,l}{Canc}_{0,1,i,l,1}$$

$$R_{1,1,i+1,l,1}=\left( 1-d_{1,i}-w_{1,i}-\mu_{1} \right)R_{1,1,i,l,1}+v_{1,i}{\gamma R}_{1,1,i+1,l,0}+r_{1}I_{1,1,i,l,0}+\rho_{1}{{LAIN}_{1,1,i,l,1}+\phi}_{1}{HAIN}_{1,1,i,l,1}+\lambda_{HIV,i,l}R_{0,1,i,l,1}$$

Sexually active, HIV positive, attending clinics, vaccine failure:

$$S_{1,1,i+1,l,2}=\left( 1-d_{1,i}-\lambda_{HPV,1,yi,l} \right)S_{1,1,i,l,2}+{v_{1,i}{(1-\gamma)S}_{1,1,i+1,l,0}+\mu}_{1}R_{1,1,i,l,1}+w_{1,i}S_{1,1,i,l,1}+\lambda_{HIV,i,l}S_{0,1,i,l,2}$$

$$I_{0,1,i+1,l,2}=\left( 1-d_{1,i}-w_{1,i}-r_{1}-\sigma_{1} \right)I_{1,1,i,l,2}+v_{1,i}{(1-\gamma)I}_{1,1,i+1,l,0}+\lambda_{HPV,1,yi,l}S_{1,1,i,l,2}+w_{1,i}I_{1,1,i,l,1}+\lambda_{HIV,i,l}I_{0,1,i,l,2}$$

$${LAIN}_{1,1,i+1,l,2}=\left( 1-d_{1,i}-\chi_{1}-\rho_{1} \right){LAIN}_{1,1,i,l,2}+\sigma_{1}I_{1,1,i,l,2}+v_{1,i}(1-\gamma){LAIN}_{1,1,i,l,0}{+ w}_{1,i}{LAIN}_{1,1,i,l,1}+\lambda_{HIV,i,l}{LAIN}_{0,1,i,l,2}$$

$${HAIN}_{1,1,i+1,l,2}=\left( 1-d_{1,i}-\omega_{1,i}-\phi_{1} \right){HAIN}_{1,1,i,l,2}+\chi_{1}{LAIN}_{1,1,i,l,2}+v_{1,i}(1-\gamma){HAIN}_{1,1,i,l,0}{+ w}_{1,i}{HAIN}_{1,1,i,l,1}+\lambda_{HIV,i,l}{HAIN}_{0,1,i,l,2}$$

$${Canc}_{1,1,i+1,l,2}=\left( 1-d_{1,i} \right){Canc}_{1,1,i,l,2}+\omega_{1,i}{HAIN}_{1,1,i,l,2}+v_{1,i}(1-\gamma){Canc}_{1,1,i,l,0}{+ w}_{1,i}{Canc}_{1,1,i,l,1}+\lambda_{HIV,i,l}{Canc}_{0,1,i,l,2}$$

$$R_{0,1,i+1,l,2}=\left( 1-d_{1,i}-w_{1,i}-\mu_{1} \right)R_{1,1,i,l,2}+v_{1,i}(1-{\gamma)R}_{1,1,i+1,l,0}+r_{1}I_{1,1,i,l,2}{+\rho_{1}{{LAIN}_{1,1,i,l,2}+\phi}_{1}{HAIN}_{1,1,i,l,2}+w}_{1,i}R_{1,1,i,l,1}+\lambda_{HIV,i,l}R_{0,1,i,l,2}$$

*HPV-6/11*

Not sexually active:

$${NA}_{0,0,i+1,l,0}=\left( 1-d_{0,i}-a_{i,l} \right){NA}_{0,0,i,l,0}$$

Sexually active, HIV negative, not attending clinics, not vaccinated:

$$S_{0,0,i+1,l,0}=\left( 1-d_{0,i}-\lambda_{HIV,i,l}-\left( c_{i}+\lambda_{HPV,0,i,l}\beta_{0} \right)-\lambda_{HPV,0,i,l} \right)S_{0,0,i,l,0}+\mu_{0}R_{0,0,i,l,0}+a_{i,l}{NA}_{0,0,i,l,0}$$

$$I_{0,0,i+1,l,0}=\left( 1-d_{0,i}-\lambda_{HIV,i,l}-c_{i}-r_{0} \right)I_{0,0,i,l,0}+\lambda_{HPV,0,i,l}{(1-\beta_{0})S}_{0,0,i,l,0}$$

$$R_{0,0,i+1,l,0}=\left( 1-d_{0,i}-\lambda_{HIV,i,l}-c_{i}-\mu_{0} \right)R_{0,0,i,l,0}+r_{0}I_{0,0,i,l,0}$$

Sexually active, HIV negative, attending clinics, not vaccinated:

$$S_{0,1,i+1,l,0}=\left( 1-d_{0,i}-\lambda_{HIV,i,l}-v_{0,i}-\lambda_{HPV,0,i,l} \right)S_{0,1,i,l,0}+\mu_{0}R_{0,1,i,l,0}+\left( c_{i}-\lambda_{HPV,0,i,l}\beta_{0} \right)S_{0,0,i,l,0}$$

$$I_{0,1,i+1,l,0}=\left( 1-d_{0,i}-\lambda_{HIV,i,l}-v_{0,i}-r_{0} \right)I_{0,1,i,l,0}+\lambda_{HPV,0,i,l}(1-\beta_{0})S_{0,1,i,l,0}+c_{i}I_{0,1,i,l,0}$$

$$R_{0,1,i+1,l,0}=\left( 1-d_{0,i}-\lambda_{HIV,i,l}-v_{0,i}-\mu_{0} \right)R_{0,1,i,l,0}+r_{0}I_{0,1,i,l,0}+c_{i}R_{0,1,i,l,0}$$

Sexually active, HIV positive, attending clinics, not vaccinated:

$$S_{1,1,i+1,l,0}=\left( 1-d_{1,i}-v_{1,i}-\lambda_{HPV,1,i,l} \right)S_{1,1,i,l,0}+\mu_{1}R_{1,1,i,l,0}+ \lambda_{HIV,i,l}(S_{0,0,i,l,0}+S_{0,1,i,l,0})$$

$$I_{1,1,i+1,l,0}=\left( 1-d_{1,i}-v_{1,i}-r_{1} \right)I_{1,1,i,l,0}+\lambda_{HPV,1,i,l}{(1-\beta_{1})S}_{1,1,i,l,0}+ \lambda_{HIV,i,l}(I_{0,0,i,l,0}+I_{0,1,i,l,0})$$

$$R_{1,1,i+1,l,0}=\left( 1-d_{1,i}-v_{1,i}-\mu_{1} \right)R_{1,1,i,l,0}+r_{1}I_{1,1,i,l,0}+ \lambda_{HIV,i,l}(R_{0,0,i,l,0}+R_{0,1,i,l,0})$$

Sexually active, HIV negative, attending clinics, vaccinated:

$$S_{0,1,i+1,l,1}=\left( 1-d_{0,i}-\lambda_{HIV,i,l}-w_{0,i} \right)S_{0,1,i,l,1}+{v_{0,i}{\gamma S}_{0,1,i,l,0}+\mu}_{0}R_{0,1,i,l,1}$$

$$I_{0,1,i+1,l,1}=\left( 1-d_{0,i}-\lambda_{HIV,i,l}-w_{0,i}-r_{0} \right)I_{0,1,i,l,1}+v_{0,i}{\gamma I}_{0,1,i,l,0}+\lambda_{HPV,0,i,l}{\beta_{0}\gamma(S}_{0,0,i,l,0}+S_{0,1,i,l,0})$$

$$R_{0,1,i+1,l,1}=\left( 1-d_{0,i}-\lambda_{HIV,i,l}-w_{0,i}-\mu_{0} \right)R_{0,1,i,l,1}+v_{0,i}{\gamma R}_{0,1,i,l,0}+r_{0}I_{0,1,i,l,0}$$

Sexually active, HIV negative, attending clinics, vaccine failure:

$$S_{0,1,i+1,l,2}=\left( 1-d_{0,i}-\lambda_{HIV,i,l}-\lambda_{HPV,0,i,l} \right)S_{0,1,i,l,2}+{v_{0,i}{\left( 1-\gamma\right)S}_{0,1,i,l,0}+\mu}_{0}R_{0,1,i,l,1}+w_{0,i}S_{0,1,i,l,1}$$

$$I_{0,1,i+1,l,2}=\left( 1-d_{0,i}-\lambda_{HIV,i,l}-r_{0} \right)I_{0,1,i,l,2}+v_{0,i}{(1-\gamma)I}_{0,1,i,l,0}+{\lambda_{HPV,0,i,l}{\beta_{0}(1-\gamma)(S}_{0,0,i,l,0}+S_{0,1,i,l,0})+\lambda}_{HPV,0,i,l}S_{0,1,i,l,2}+w_{0,i}I_{0,1,i,l,1}$$

$$R_{0,1,i+1,l,2}=\left( 1-d_{0,i}-\lambda_{HIV,i,l}-\mu_{0} \right)R_{0,1,i,l,2}+v_{0,i}(1-{\gamma)R}_{0,1,i,l,0}+r_{0}I_{0,1,i,l,2}+w_{0,i}R_{0,1,i,l,1}$$

Sexually active, HIV positive, attending clinics, vaccinated:

$$S_{1,1,i+1,l,1}=\left( 1-d_{1,i}-w_{0,i} \right)S_{1,1,i,l,1}+{v_{1,i}{\gamma S}_{1,1,i+1,l,0}+\mu}_{1}R_{1,1,i,l,1}+\lambda_{HIV,i,l}S_{0,1,i,l,1}$$

$$I_{1,1,i+1,l,1}=\left( 1-d_{1,i}-w_{1,i}-r_{1} \right)I_{1,1,i,l,1}+v_{1,i}{\gamma I}_{1,1,i+1,l,0}+{\lambda_{HPV,1,i,l}{\beta_{1}\gamma S}_{1,1,i,l,0}+\lambda}_{HIV,i,l}I_{0,1,i,l,1}$$

$$R_{1,1,i+1,l,1}=\left( 1-d_{1,i}-w_{1,i}-\mu_{1} \right)R_{1,1,i,l,1}+v_{1,i}{\gamma R}_{1,1,i+1,l,0}+r_{1}I_{1,1,i,l,0}+\lambda_{HIV,i,l}R_{0,1,i,l,1}$$

Sexually active, HIV positive, attending clinics, vaccine failure:

$$S_{1,1,i+1,l,2}=\left( 1-d_{1,i}-\lambda_{HPV,1,i,l} \right)S_{1,1,i,l,2}+{v_{1,i}{(1-\gamma)S}_{1,1,i+1,l,0}+\mu}_{1}R_{1,1,i,l,1}+w_{1,i}S_{1,1,i,l,1}+\lambda_{HIV,i,l}S_{0,1,i,l,2}$$

$$I_{0,1,i+1,l,2}=\left( 1-d_{1,i}-w_{1,i}-r_{1} \right)I_{1,1,i,l,2}+v_{1,i}{(1-\gamma)I}_{1,1,i+1,l,0}+{\lambda_{HPV,1,i,l}{\beta_{1}(1-\gamma)S}_{1,1,i,l,0}+\lambda}_{HPV,1,i,l}S_{1,1,i,l,2}+w_{1,i}I_{1,1,i,l,1}+\lambda_{HIV,i,l}I_{0,1,i,l,2}$$

$$R_{0,1,i+1,l,2}=\left( 1-d_{1,i}-w_{1,i}-\mu_{1} \right)R_{1,1,i,l,2}+v_{1,i}(1-{\gamma)R}_{1,1,i+1,l,0}+r_{1}I_{1,1,i,l,2}+w_{1,i}R_{1,1,i,l,1}+\lambda_{HIV,i,l}R_{0,1,i,l,2}$$

# A7. Computer simulations

Custom written C++ code (compiled using GCC v4.8.3 from the GNU Project) was used for fitting baseline HPV-6/11/16/18-related anogenital warts and anal cancer incidences and for modelling the effect of vaccination introduction on these disease outcomes. Custom written R code (using R version 3.2.2 from the R Foundation for Statistical Computing, Vienna, Austria, including packages bblme, dplyr, functional, ggplot2, grid, lazyeval, lhs, mgcv, plyr, RColorBrewer, reshape2, scales, stringr and triangle) was used for the economic analysis of the disease outcomes. Computation was conducted on the High Performance Cluster at the London School of Hygiene and Tropical Medicine.

# A9. Meta-scenario fits and transmission probabilities

Fits of all 5,000 meta-scenarios to the observed number of GUMCAD wart cases in HIV- and HIV+ MSM for HPV 6/11, and to incidence of anal cancer in England and prevalence at the Mortimer Market Centre (MMC), a GUM clinic in London, in HIV- and HIV+ MSM for HPV 16/18 are shown below. The 20% of meta-scenarios with lowest sum of least squared error are selected for further analysis. Warts and anal cancer scenarios describe the three scenarios with differing percentage contribution of HPV 6 and HPV 11 to HPV 6/11-related warts and HPV 16 and HPV 18 to HPV 16/18-related anal cancers. 95% confidence intervals are shown for HPV prevalences at MMC.

**Figure**. HPV 6 fits.


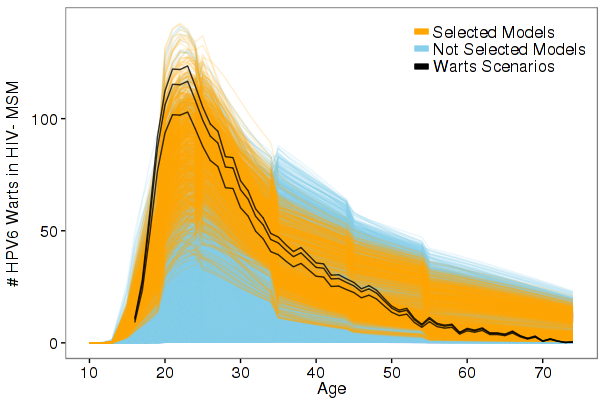

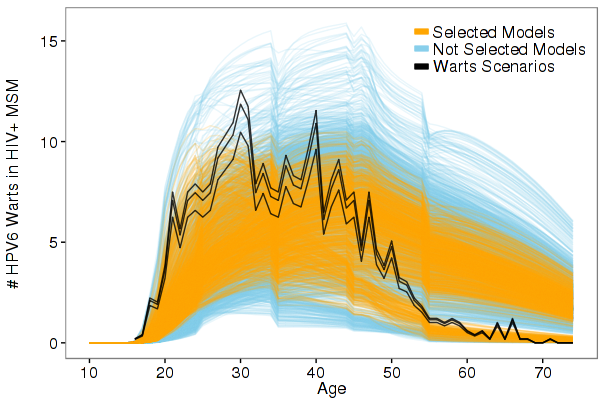


**Figure**. HPV 11 fits.


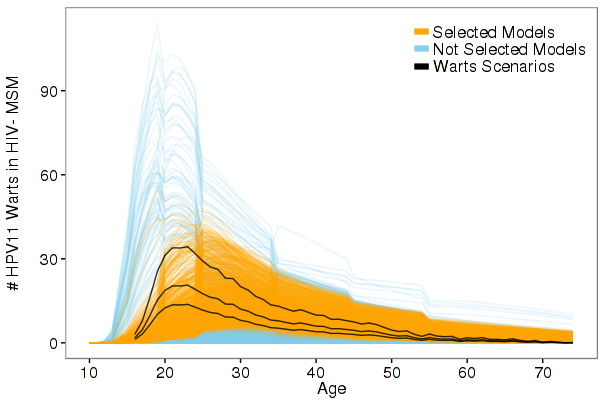

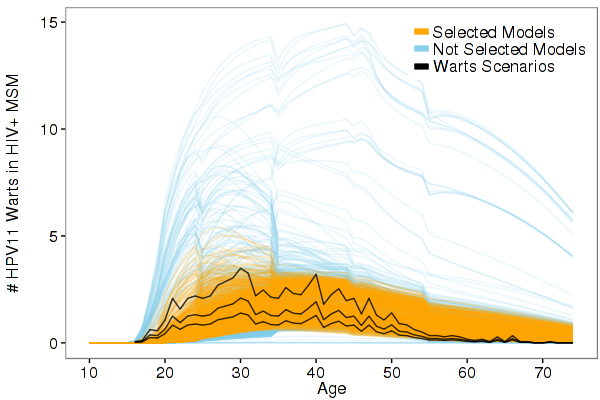


**Figure**. HPV 16 fits.


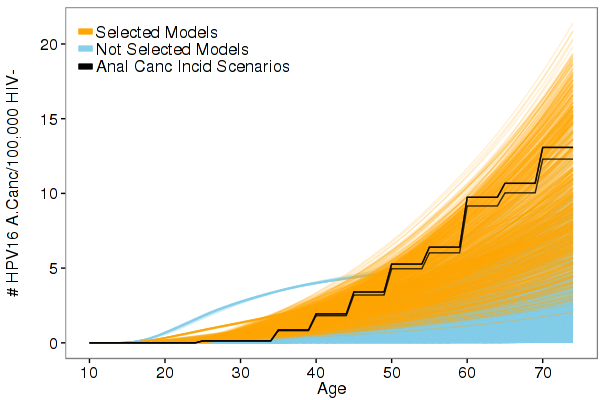

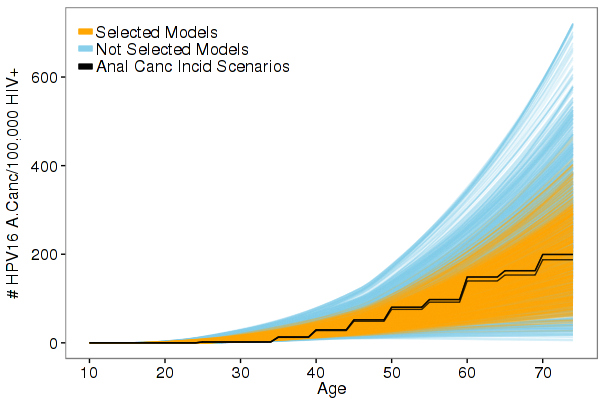


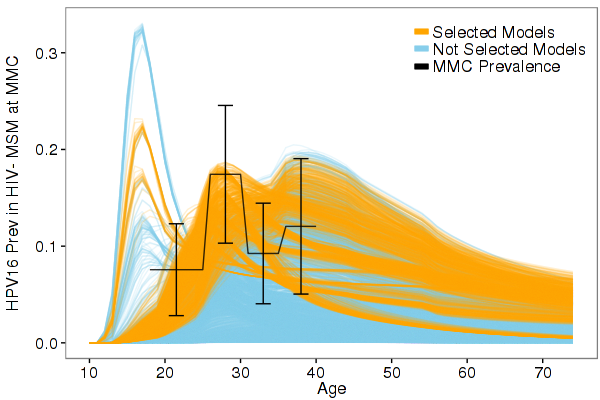

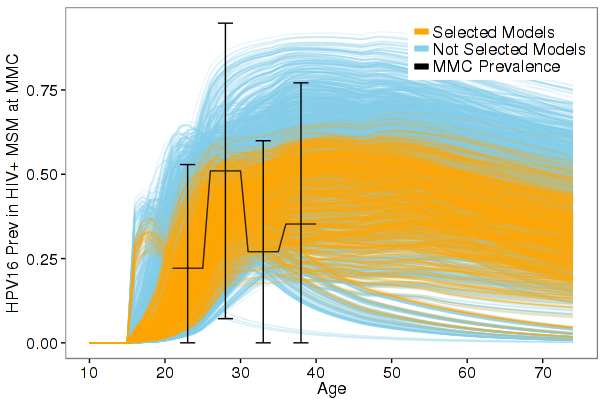


**Figure**. HPV 18 fits.


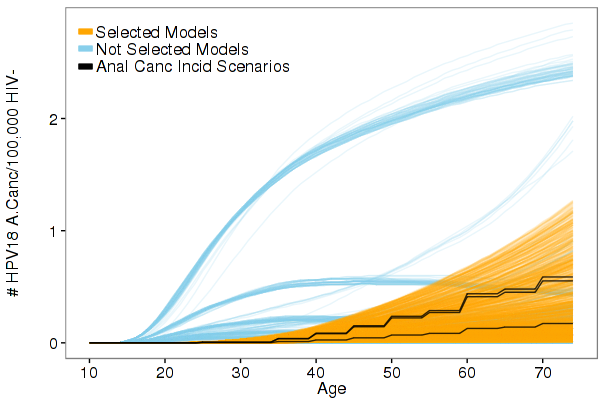

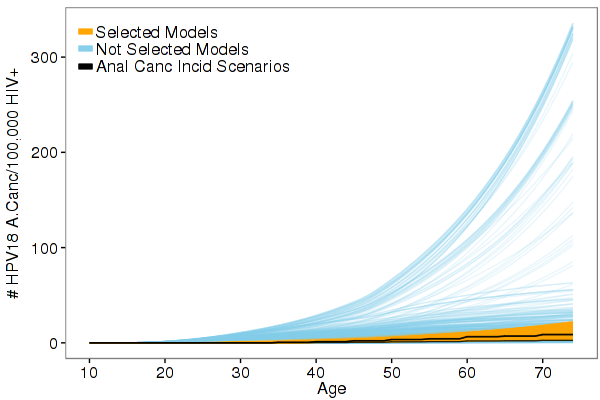


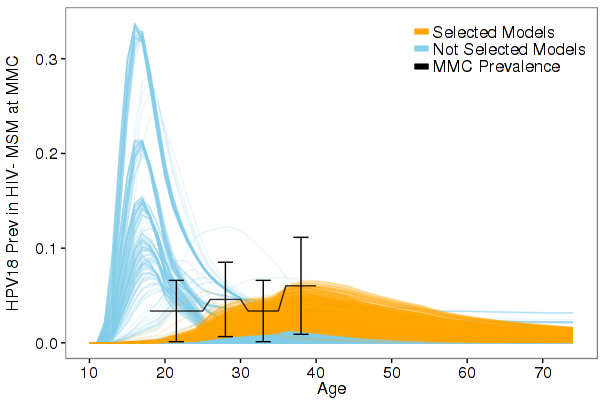

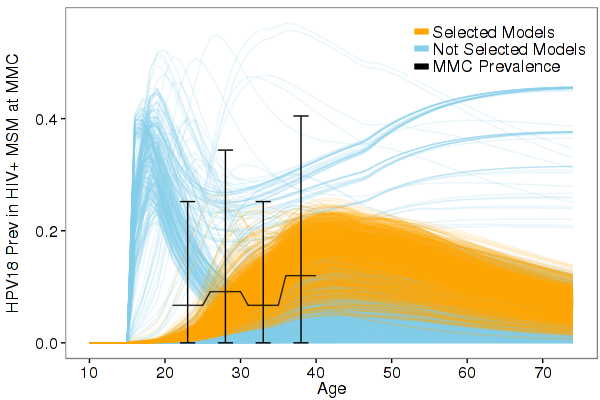


**Figure**. Number of warts or anal cancers per year in selected and not selected meta-scenarios.


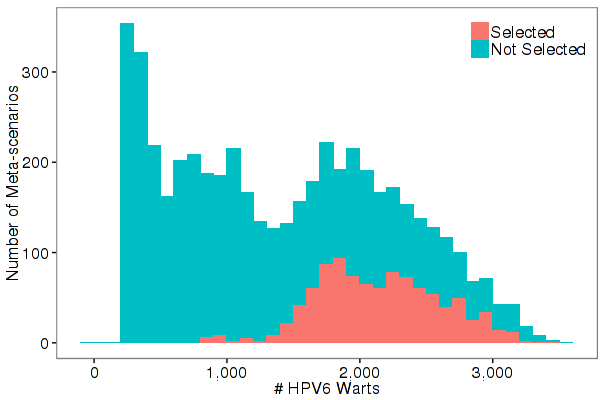

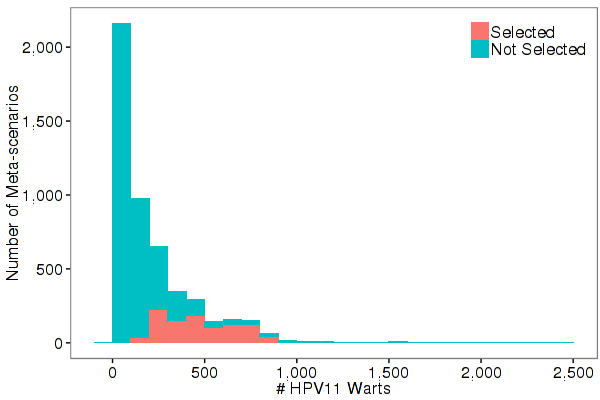


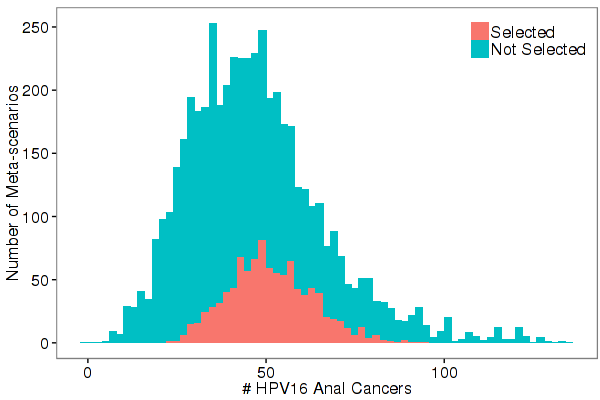

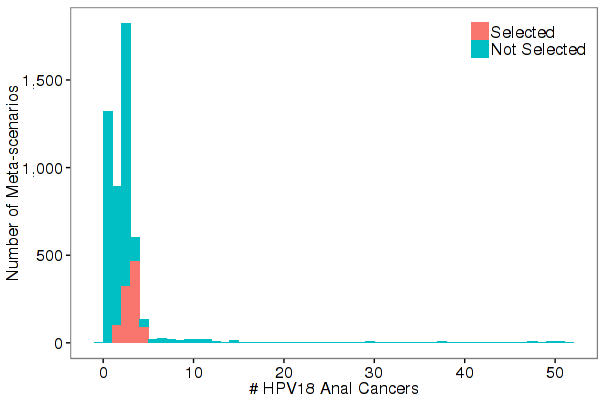


**Table**. Median (50% range) of same-sex transmission probabilities for each activity group across selected meta-scenarios.

|  | **Low Activity Group** | **Mid Activity Group** | **High Activity Group** |
| --- | --- | --- | --- |
| **HPV 6** | 0.52 (0.36 - 0.69) | 0.21 (0.16 - 0.30) | 0.10 (0.07 - 0.15) |
| **HPV 11** | 0.10 (0.06 - 0.19) | 0.07 (0.05 - 0.10) | 0.04 (0.03 - 0.06) |
| **HPV 16** | 0.19 (0.06 - 0.28) | 0.08 (0.05 - 0.14) | 0.04 (0.03 - 0.07) |
| **HPV 18** | 0.06 (0.06 - 0.06) | 0.03 (0.03 - 0.04) | 0.01 (0.02 - 0.03) |

For reference, the table below shows the heterosexual transmission probabilities fitted in previous work [4]:

**Table**. Median (50% range) of heterosexual transmission probabilities for each activity group across selected meta-scenarios, from previous model [4].

|  | **Low Activity Group** | **Mid Activity Group** | **High Activity Group** |
| --- | --- | --- | --- |
| **HPV 6** | 0.33 (0.22 – 0.49) | 0.08 (0.05 – 0.16) | 0.02 (0.01 – 0.07) |
| **HPV 11** | 0.13 (0.09 – 0.19) | 0.05 (0.04 – 0.07) | 0.02 (0.02 – 0.03) |
| **HPV 16** | 0.36 (0.21 – 0.71) | 0.15 (0.07 – 0.40) | 0.08 (0.16 – 0.38) |
| **HPV 18** | 0.18 (0.06 – 0.36) | 0.06 (0.04 – 0.09) | 0.03 (0.02 – 0.04) |

**Figure**. Same-sex transmission probabilities for HPV-6 meta-scenarios.


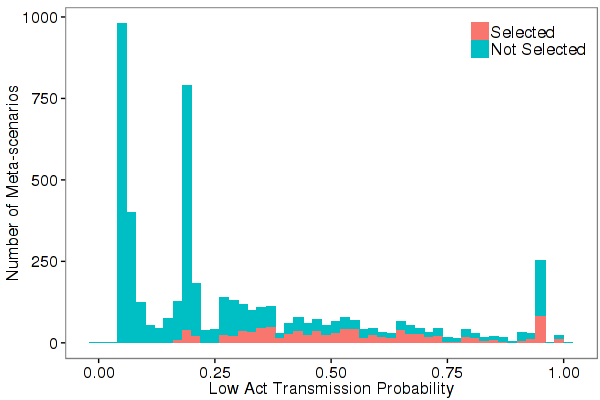

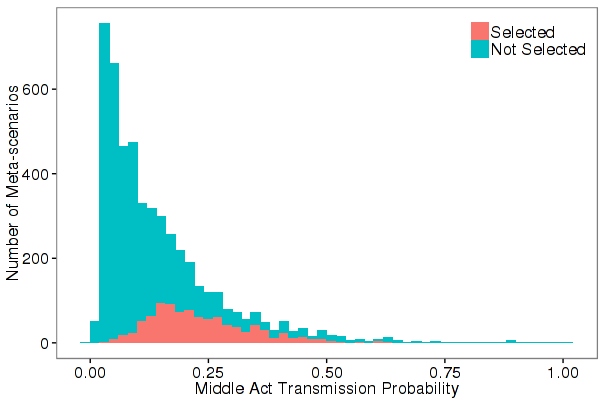


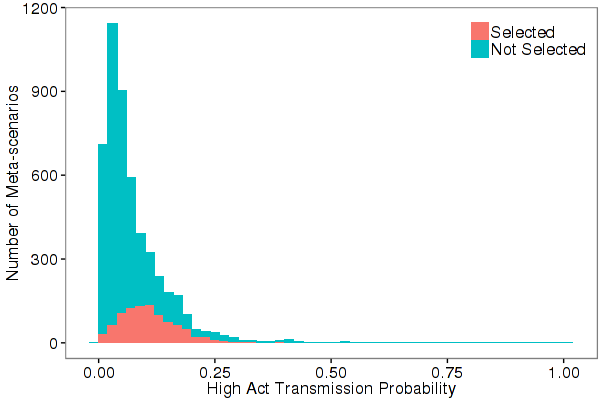


**Figure**. Same-sex transmission probabilities for HPV-11 meta-scenarios.


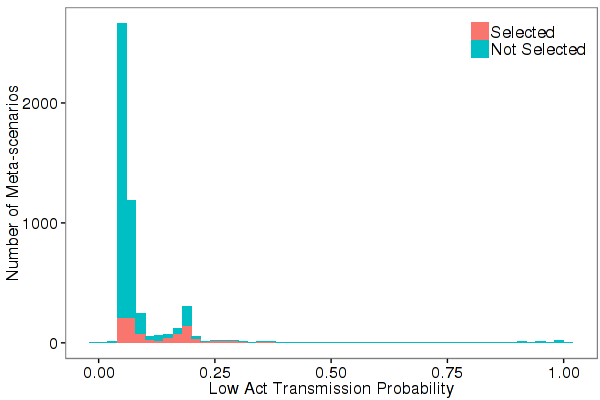

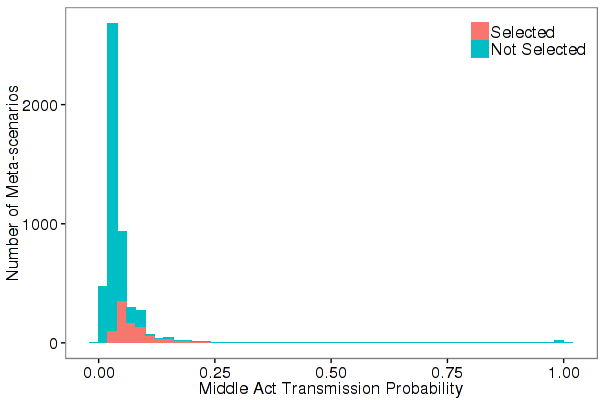


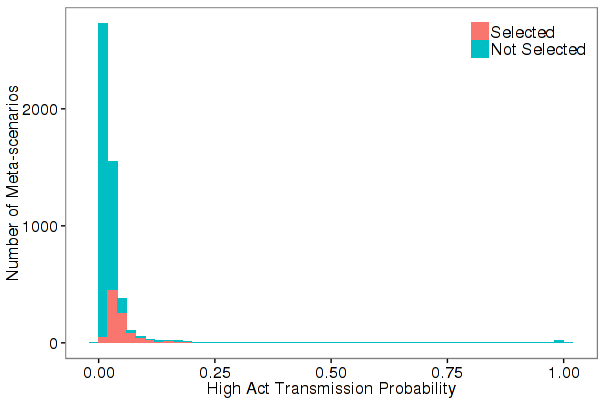


**Figure**. Same-sex transmission probabilities for HPV-16 meta-scenarios.


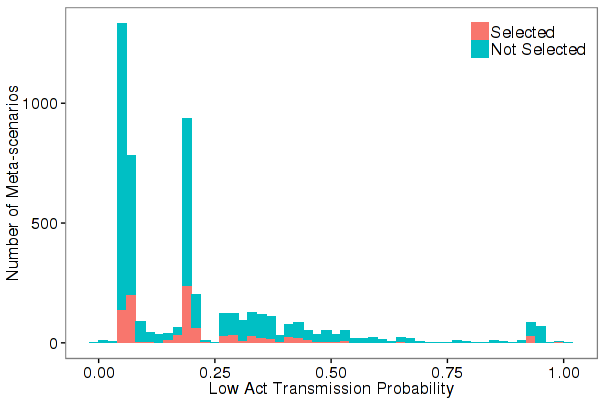

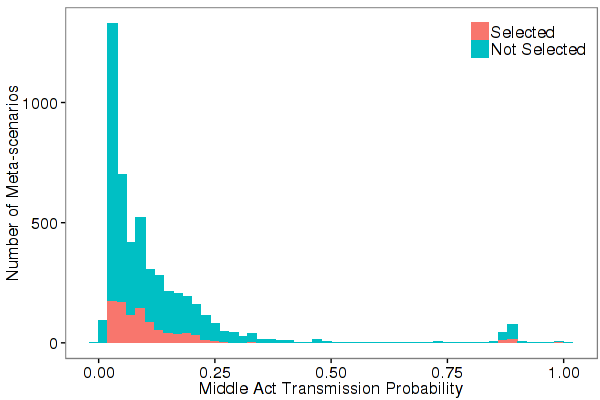


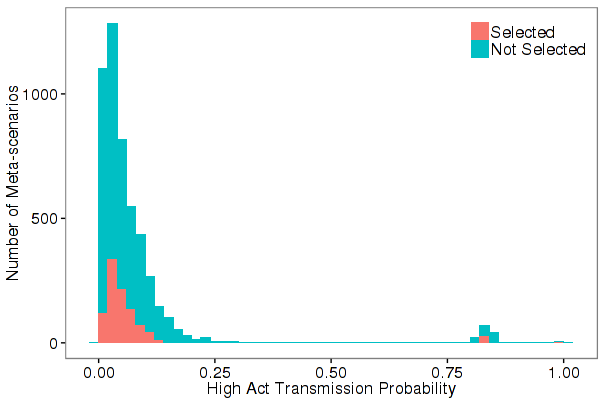


**Figure**. Same-sex transmission probabilities for HPV-18 meta-scenarios.


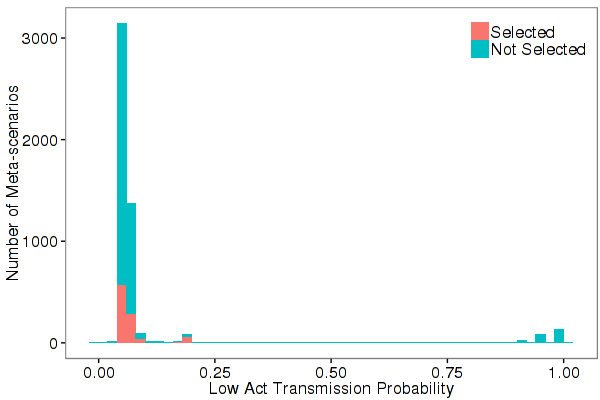

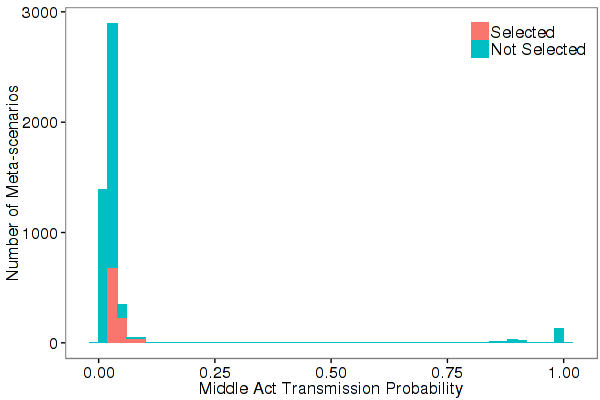


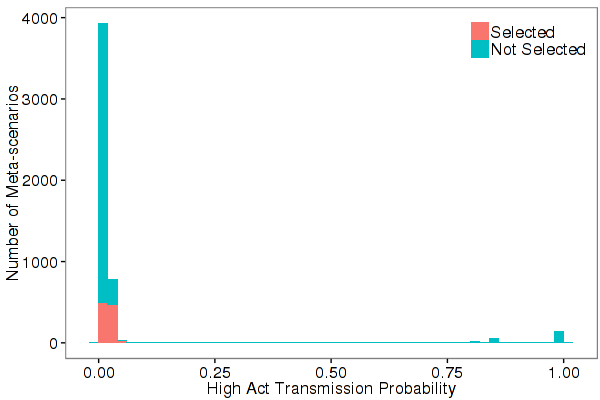


# A8. Best fitting meta-scenarios: assumptions selected

**Figure.** Distribution of clearance rate of infection in HIV-negative and HIV-positive MSM in best-fitting scenarios.

**
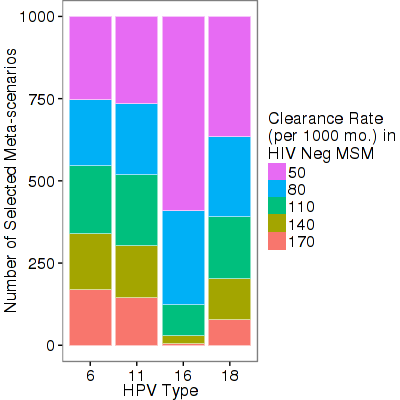

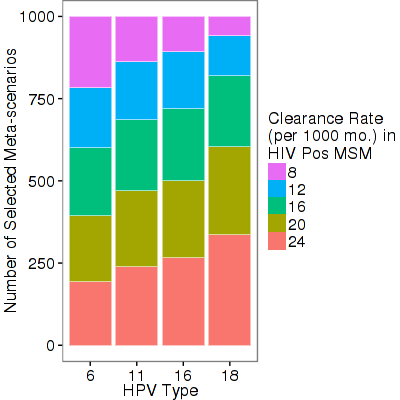
**

**Figure.** Distribution of duration of natural immunity in HIV-negative and HIV-positive MSM in best-fitting scenarios.


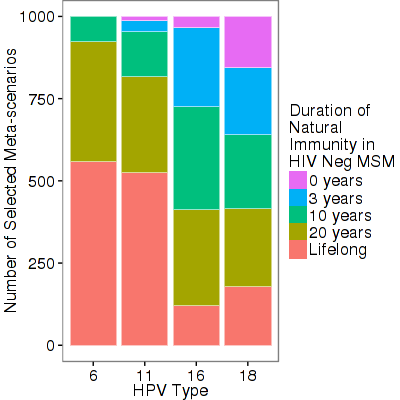

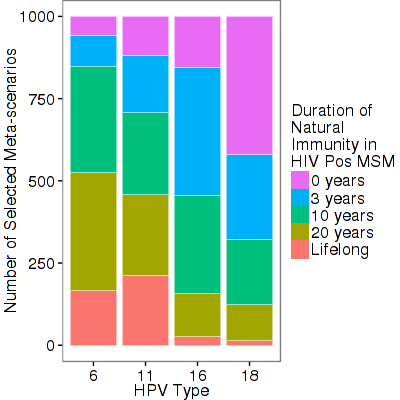


**Figure.** Distribution of activity group and HIV assortativeness in best-fitting scenarios.


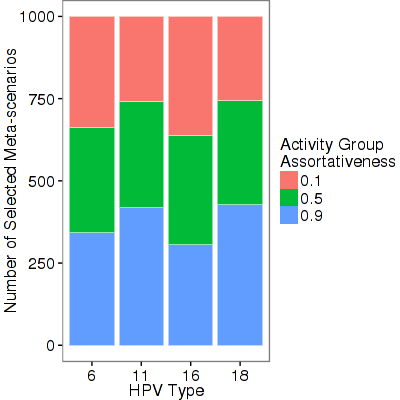

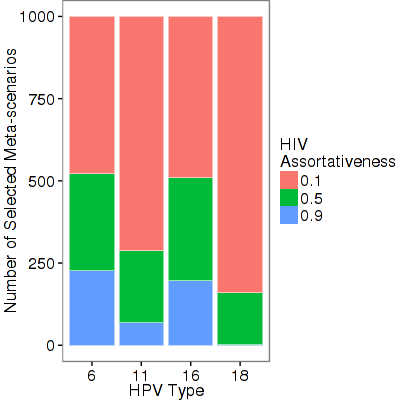


**Figure.** Distribution of percent of newly HPV-infected HIV-negative and HIV-positive with anogenital warts in best-fitting scenarios.


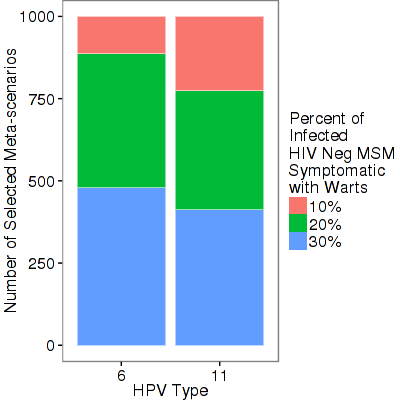

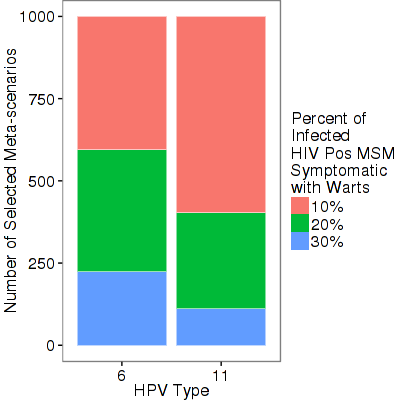


**Figure.** Distribution of percent of HPV-6/11-related wart lesions that are HPV-11-related and percent of HPV-16/18-related anal cancers HPV-18-related in best-fitting scenarios.


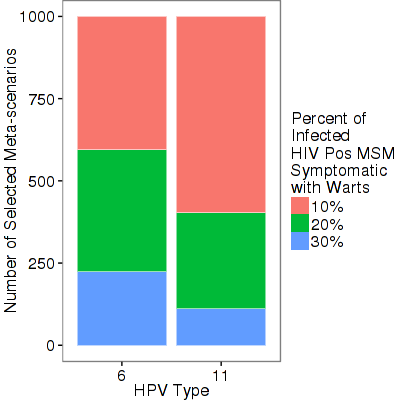

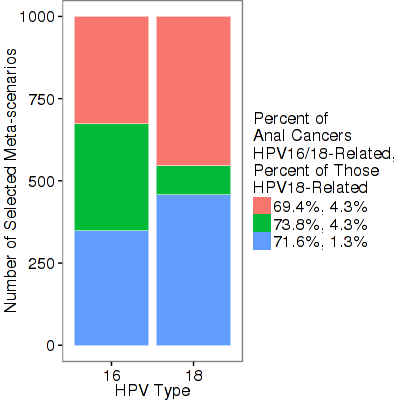


**Figure.** Distribution of partner governing transmission parameter in best-fitting scenarios.


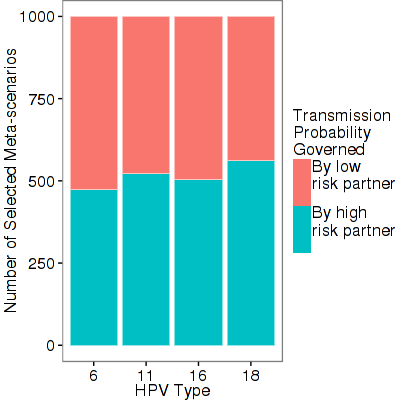


**Figure**. Joint distribution of clearance rate versus duration of natural immunity in HIV- MSM in selected meta-scenarios.


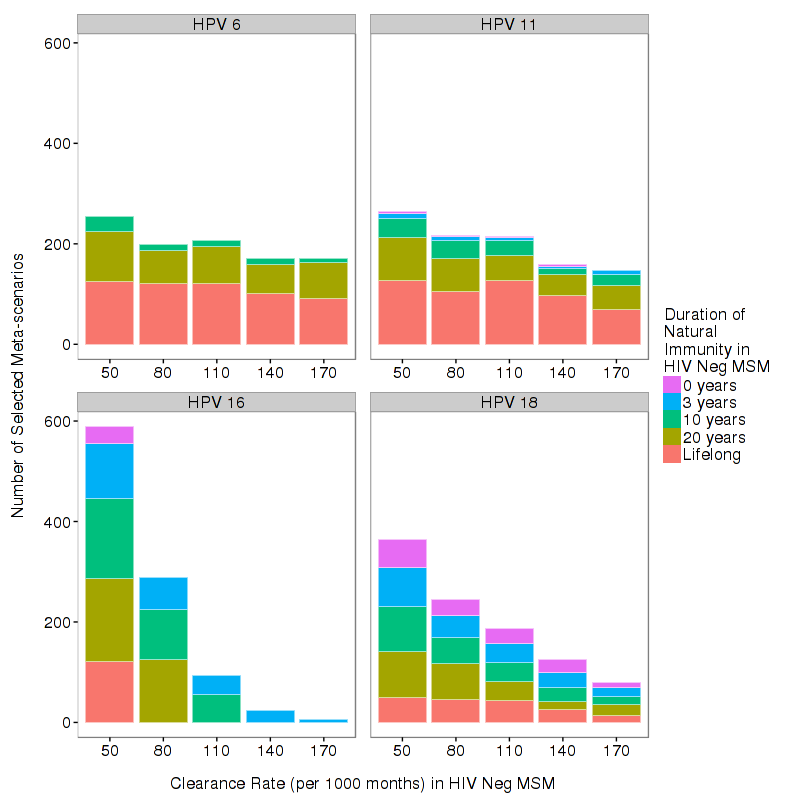


**Figure**. Joint distribution of clearance rate versus duration of natural immunity in HIV+ MSM in selected meta-scenarios.


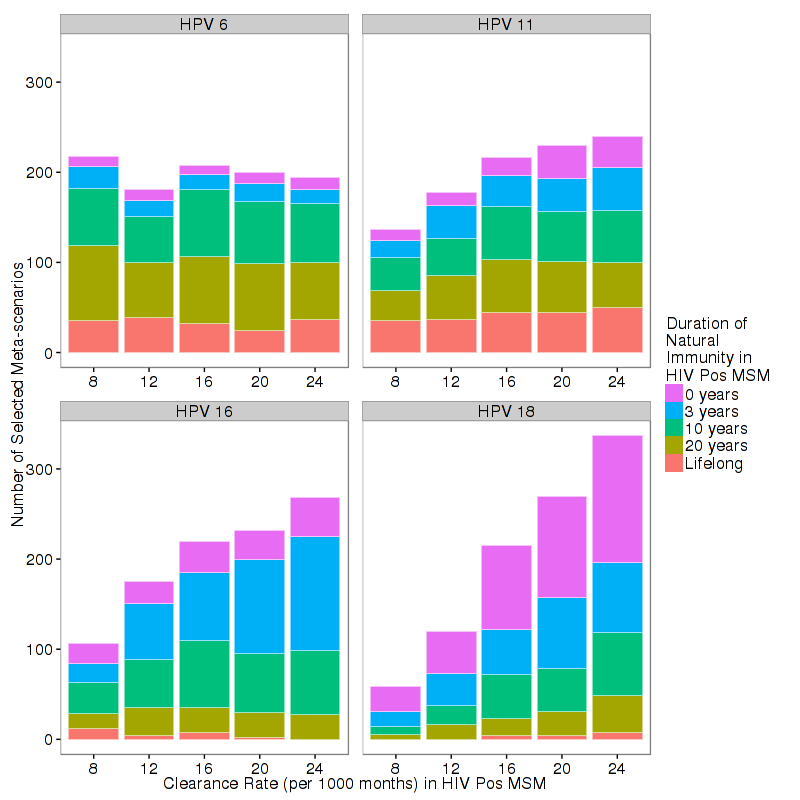


# A9. Sample model outcomes

The following figures display the number of individuals in each HPV-related state at the pre-vaccination equilibrium for the best-fitting HPV-6 and HPV-16 model. The figures also display the number of individuals only actively infected, or infected or immune, by activity group and by HIV status for these two best-fitting models.

For the best-fitting HPV-6 model, for HIV-negative MSM, natural immunity duration is lifelong, HPV clearance rate is 110 episodes/1000 person-months, and the percentage of HPV-6/11 infections causing warts is 30%. For HIV-positive MSM, natural immunity duration is also lifelong, HPV clearance rate is 12 episodes/1000 person-months, and the percentage of HPV-6/11 infections causing warts is 30%. Assortativeness by activity group is 0.1, and assortativeness by HIV status is 0.9. Transmission probability per partnership is governed by the higher-activity partner, and 25% of HPV-6/11-related warts are due to HPV-11.

For the best-fitting HPV-16 model, for HIV-negative MSM, natural immunity duration is 20 years long, and HPV clearance rate is 50 episodes/1000 person-months. For HIV-positive MSM, natural immunity duration is also 20 years long, and HPV clearance rate is 16 episodes/1000 person-months. Assortativeness by activity group is 0.9, and assortativeness by HIV status is 0.9. Transmission probability per partnership is governed by the lower-activity partner, 73.8% of anal cancers are HPV-16/18-related, and 4.3% of HPV-16/18-related anal cancers are due to HPV-16.

**Figure.** Number of individuals in each HPV-related state at baseline equilibrium (without vaccination) for the best-fitting HPV-6 model.

**Figure.** Number of individuals in each HPV-related state at baseline equilibrium (without vaccination) for the best-fitting HPV-16 model.

**Figure.** Number of individuals actively infected by activity group and by HIV status (top row) and the number of individuals either actively infected or immune by activity group and HIV status (bottom row) for the best-fitting HPV-6 model.

**Figure.** Number of individuals actively infected by activity group and by HIV status (top row) and the number of individuals either actively infected or immune by activity group and HIV status (bottom row) for the best-fitting HPV-16 model.

# A10. Model outcomes by HPV type

The following plots show the mean prevalence of HPV-6, 11, 16, or 18, number of anogenital warts cases (for HPV-6/11), and number of anal cancer cases (for HPV-16/18) across ages by HIV status for 100 years following the introduction of one of two strategies – vaccinating HIV-positive 16-to-40-year-old MSM and vaccinating all 16-to-40-year-old MSM – assuming lifelong vaccine vaccine-induced immunity. The plots also show the percent change in disease outcomes as a result of vaccinating either only HIV-positive MSM or all MSM in the age bands of 16-to-25-year-olds, 16-to-30-year-olds, 16-to-35-year-olds, 16-to-40-year-olds as a function of years since vaccination introduction.

a) Vaccinating HIV-positive 16-to-40-year-old MSM


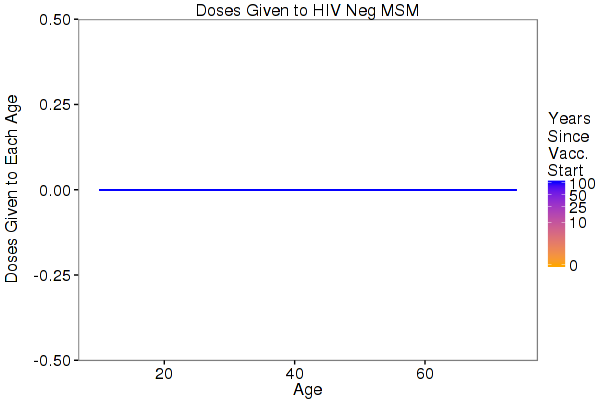

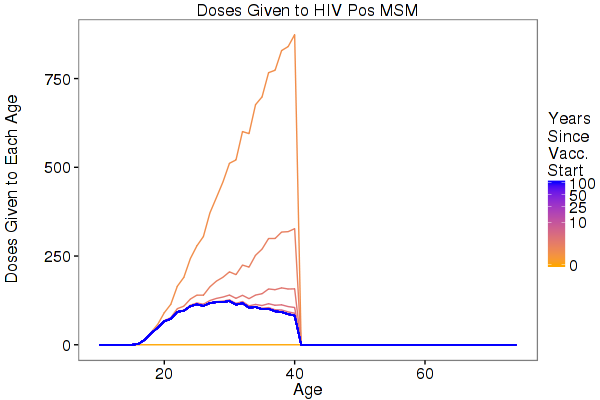


b) Vaccinating all 16-to-40-year-old MSM


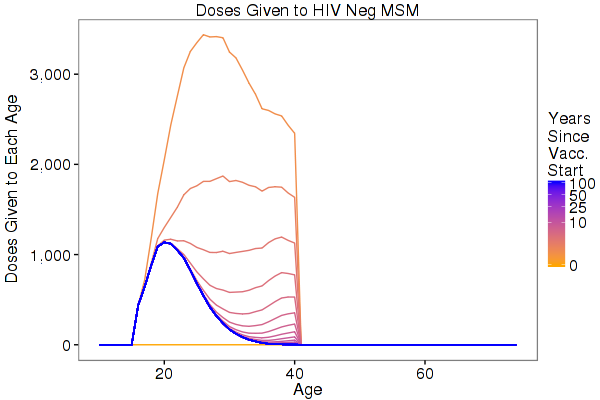

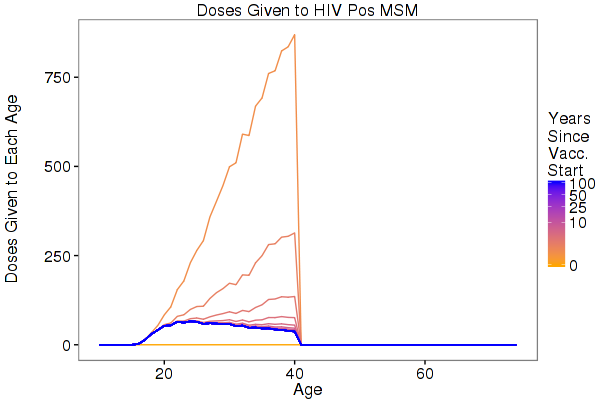


Figure. Number of annual doses given to each age by HIV status.

a) Vaccinating HIV+ 16-to-40-year-old MSM with lifelong vaccine-induced immunity


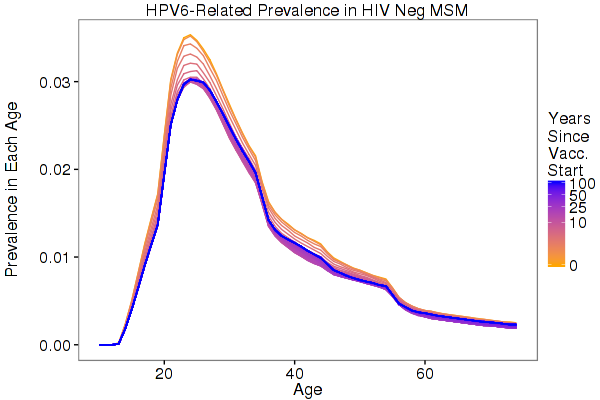

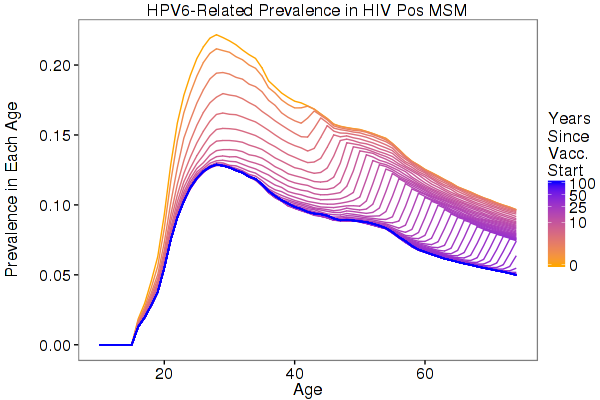


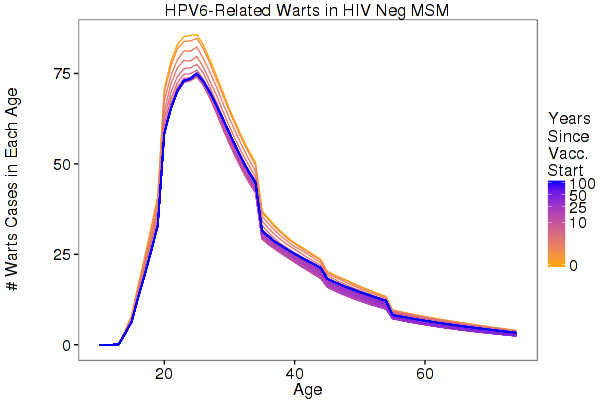

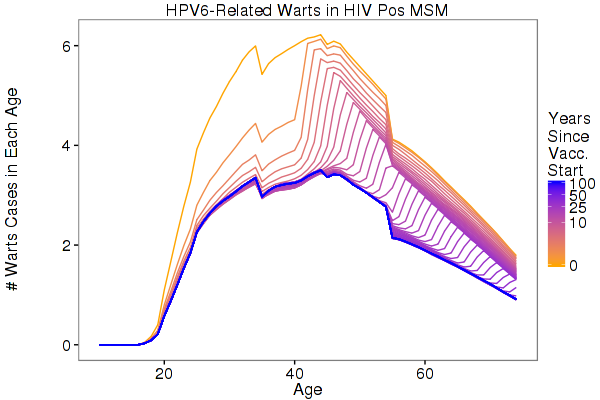


b) Vaccinating All 16-to-40-year-old MSM with lifelong vaccine-induced immunity


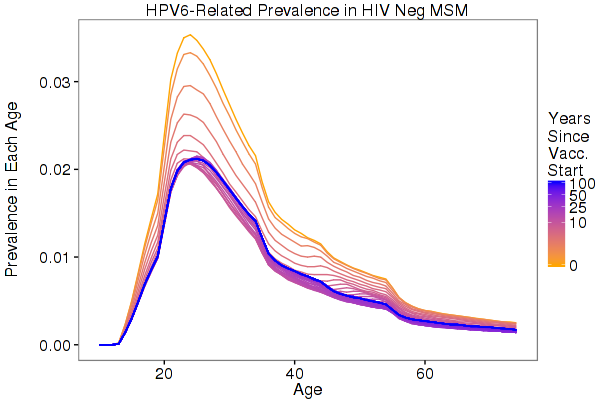

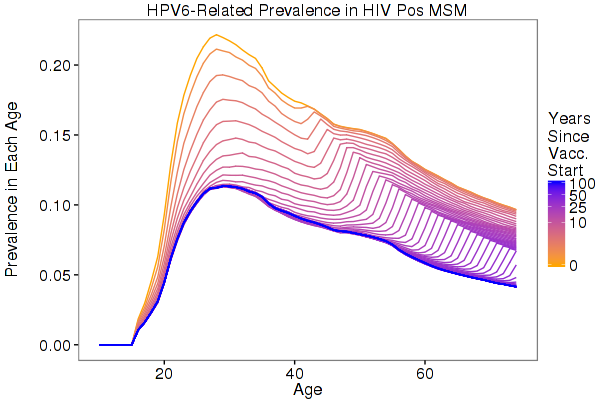


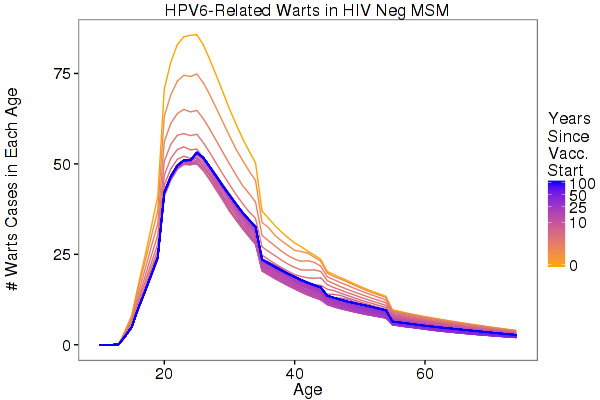

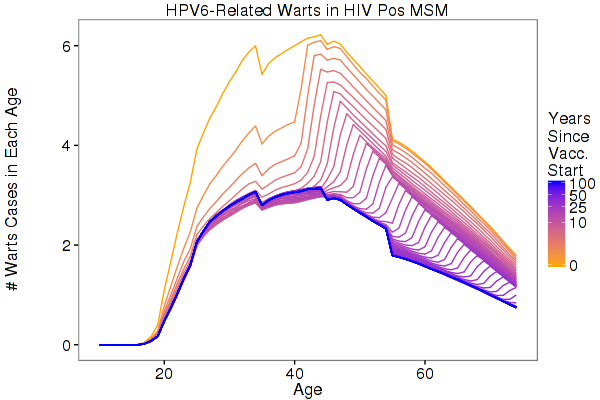


Figure. Effect of vaccination on HPV-6 prevalence and anogenital warts over 100 years with lifelong vaccine-induced immunity.

a) Vaccinating only HIV+ MSM in specified age-bands


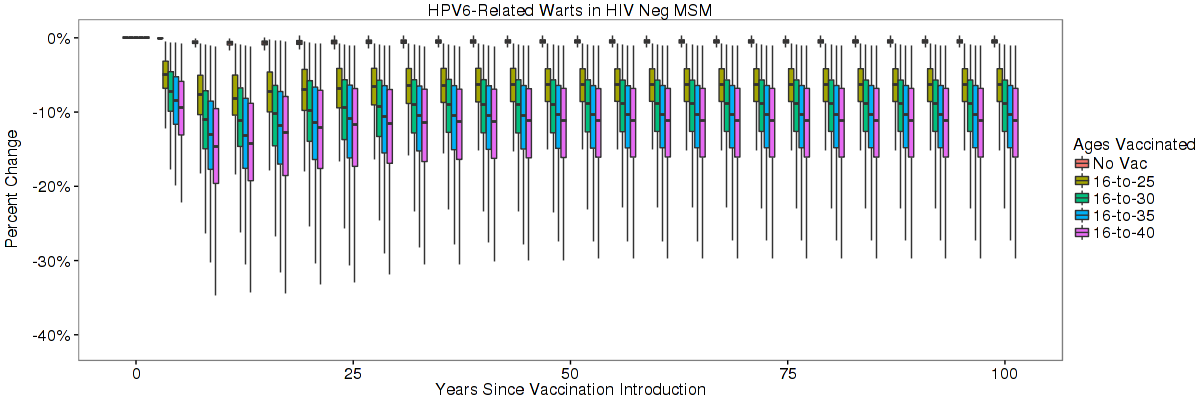


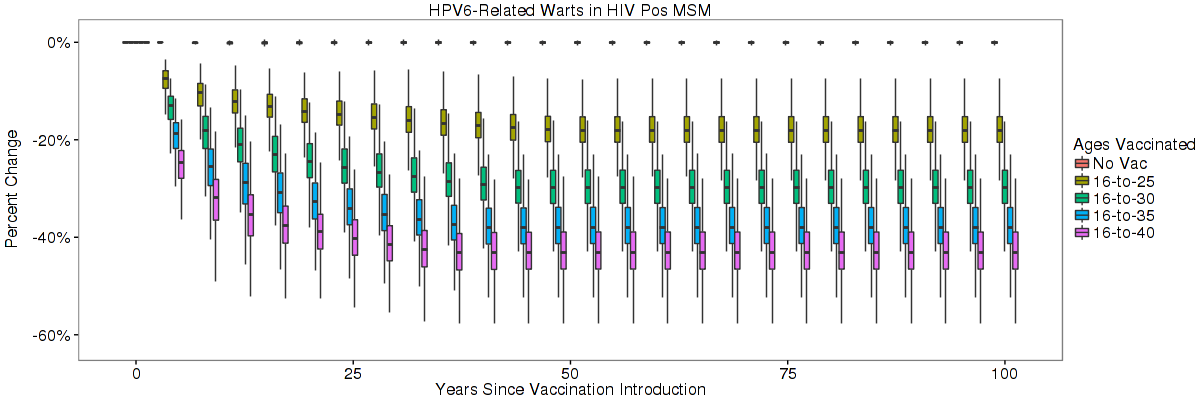


b) Vaccinating all MSM in specified age-bands


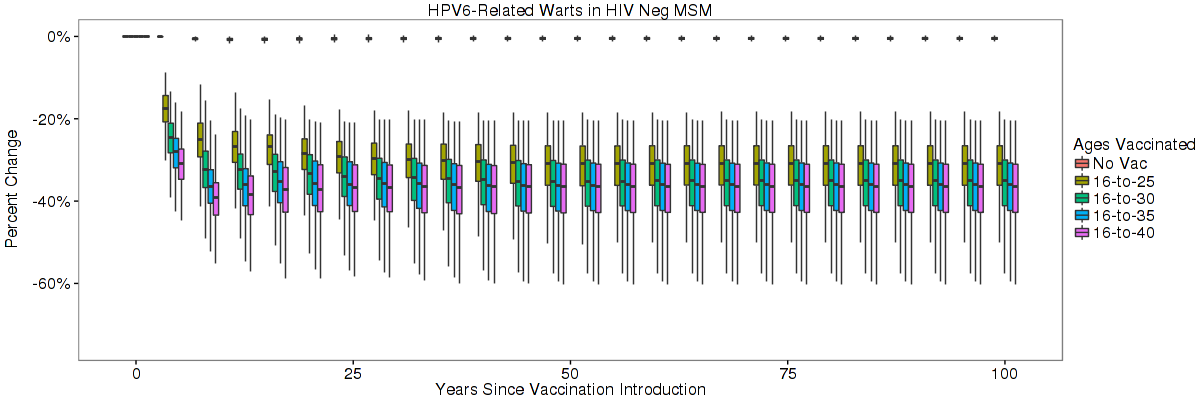


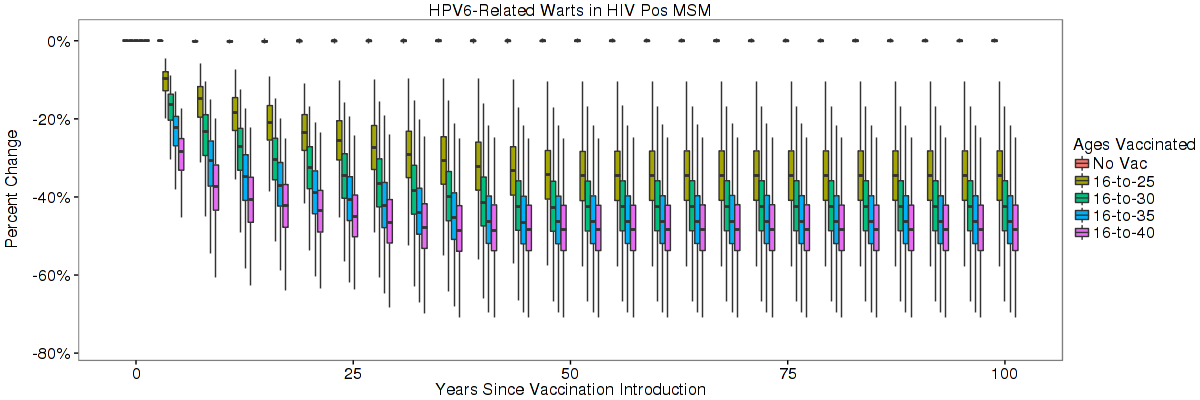


Figure. Effect of vaccination on HPV-6-related anogenital warts over 100 years with lifelong vaccine-induced immunity.

a) Vaccinating HIV+ 16-to-40-year-old MSM with lifelong vaccine-induced immunity


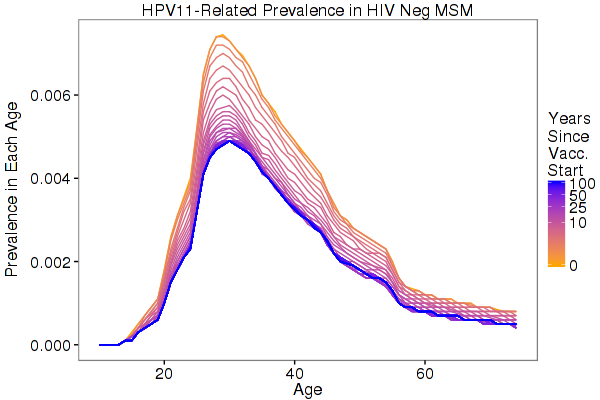

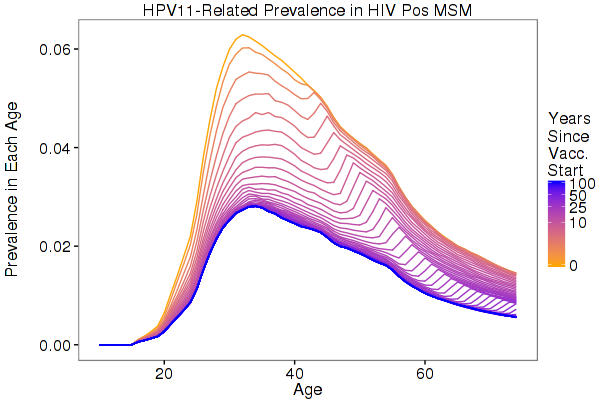


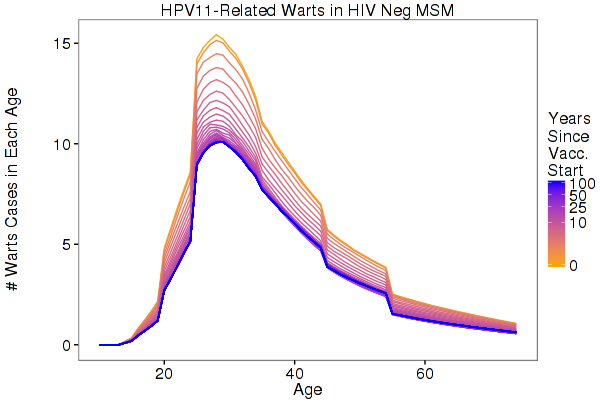

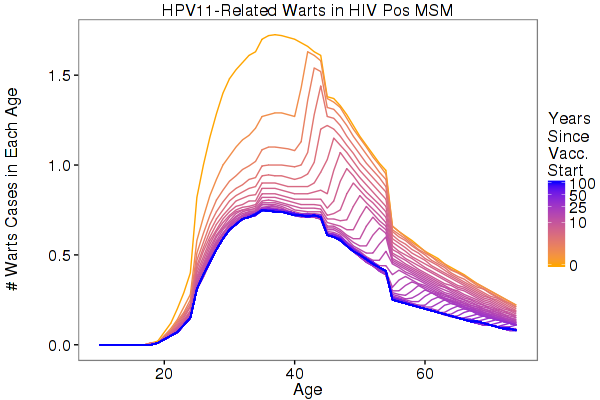


b) Vaccinating all 16-to-40-year-old MSM with lifelong vaccine-induced immunity


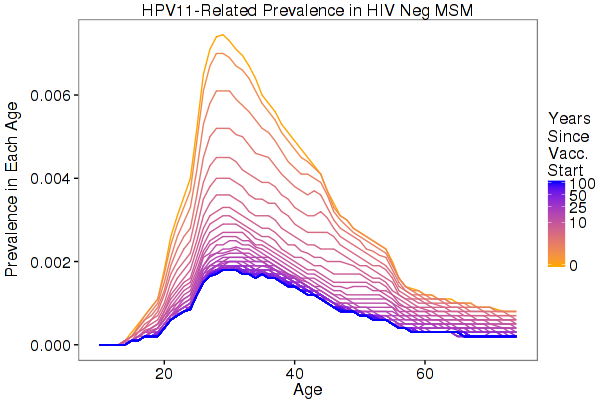

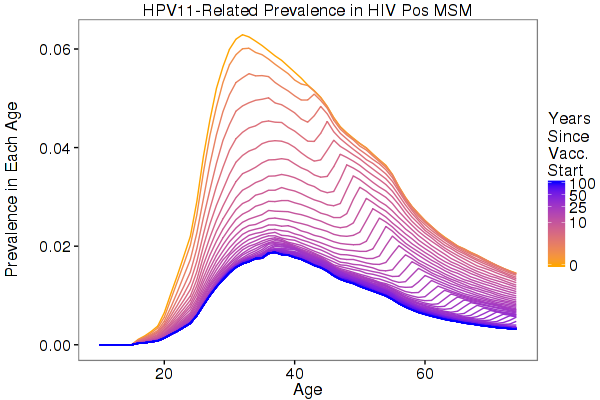


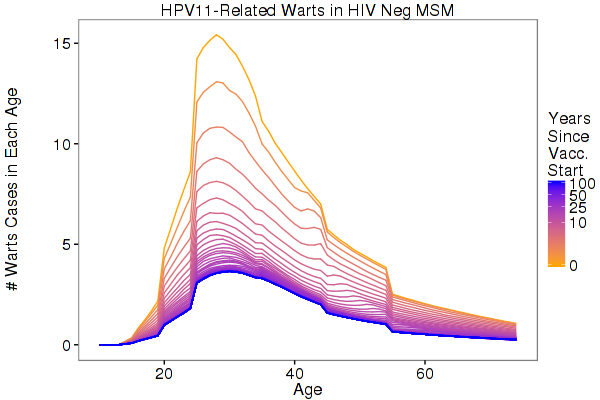

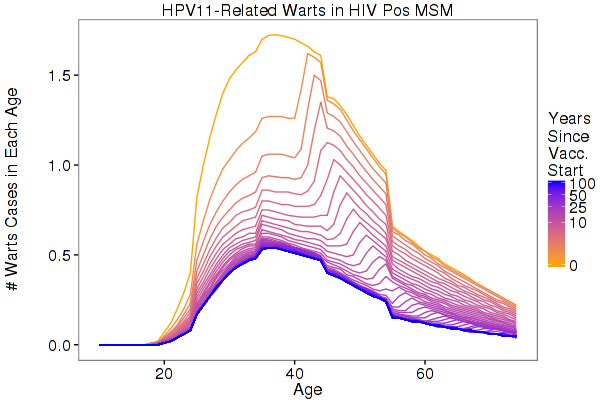


**Figure.** Effect of vaccination on HPV-11 prevalence and anogenital warts over 100 years with lifelong vaccine-induced immunity.

a) Vaccinating only HIV+ MSM in specified age-bands


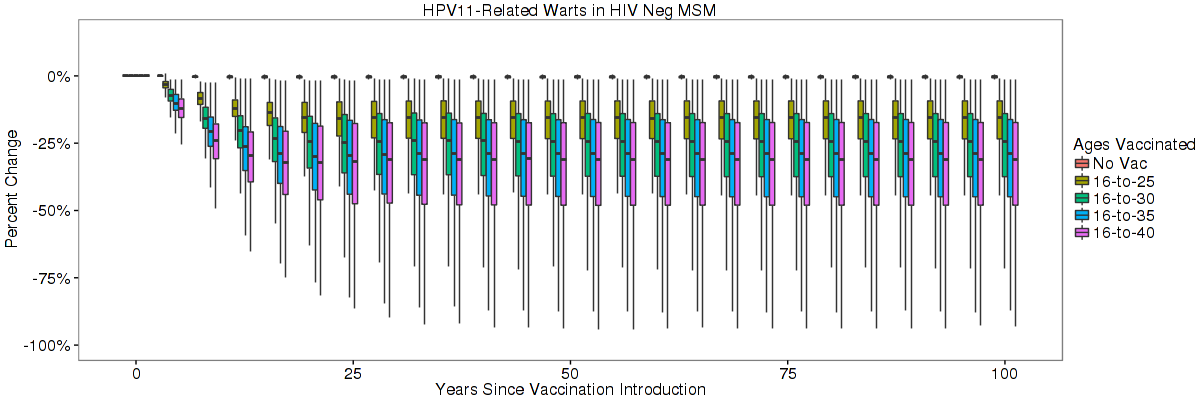

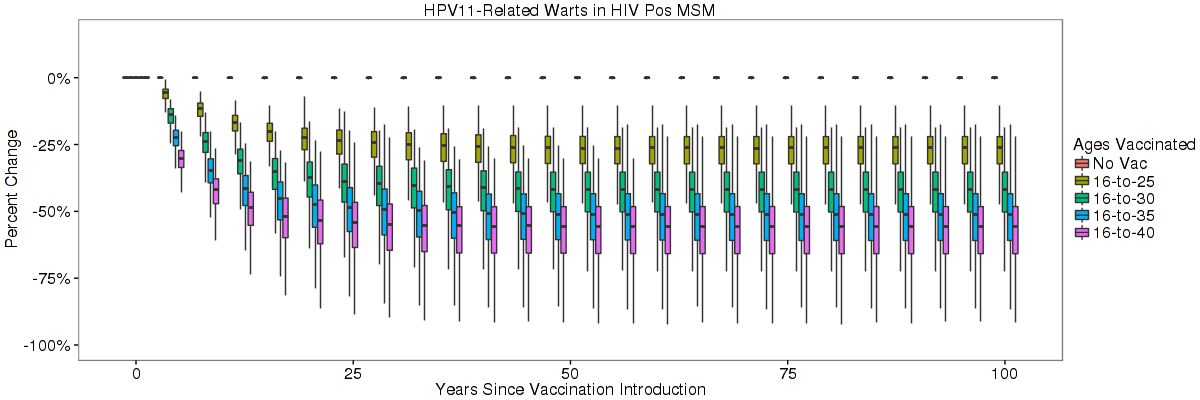


b) Vaccinating all MSM in specified age-bands


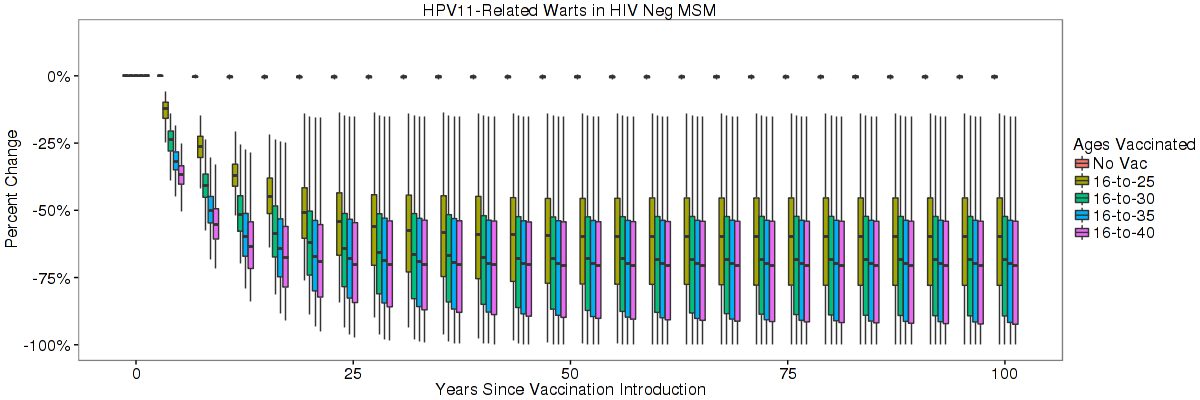


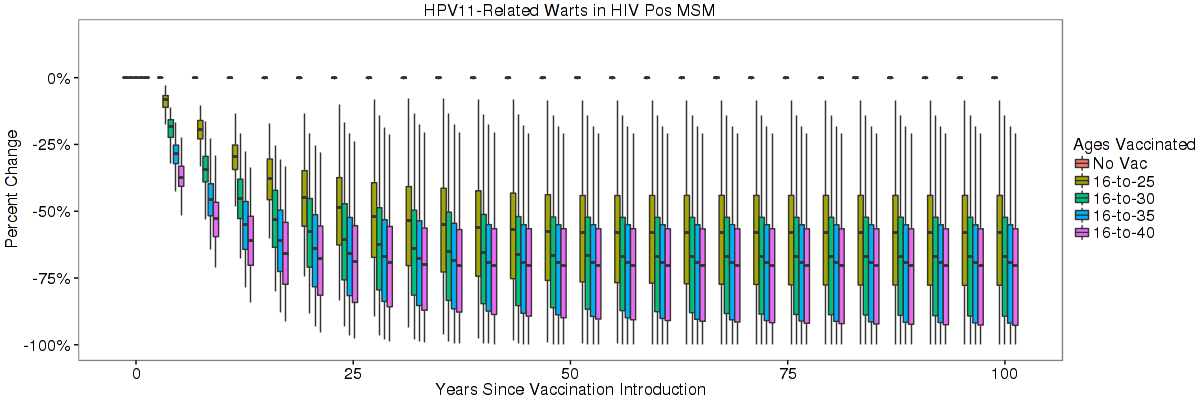


Figure. Effect of vaccination on HPV-11-related anogenital warts over 100 years with lifelong vaccine-induced immunity.

a) Vaccinating HIV+ 16-to-40-year-old MSM with lifelong vaccine-induced immunity


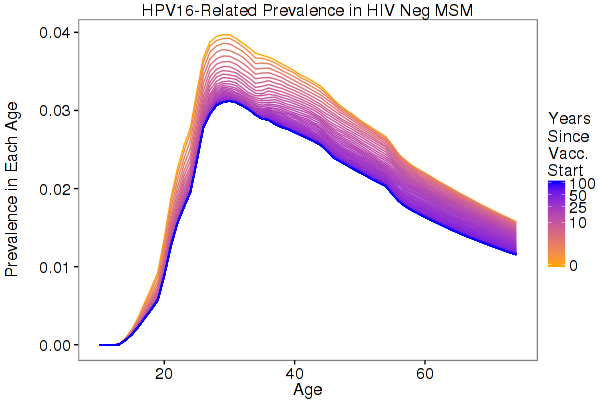

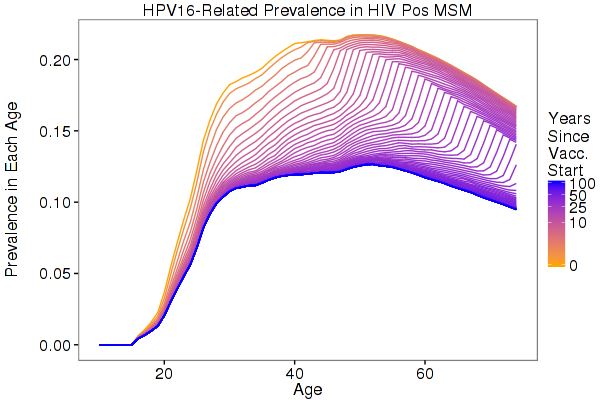


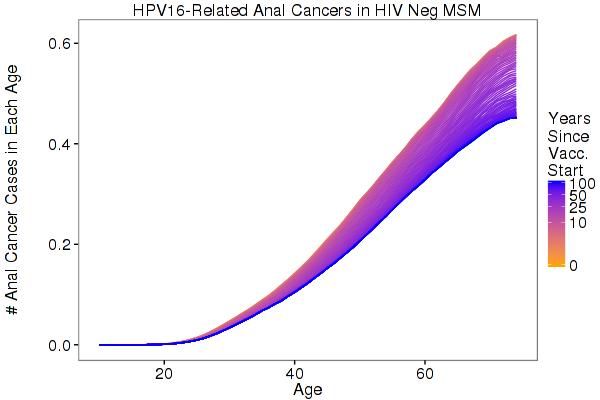

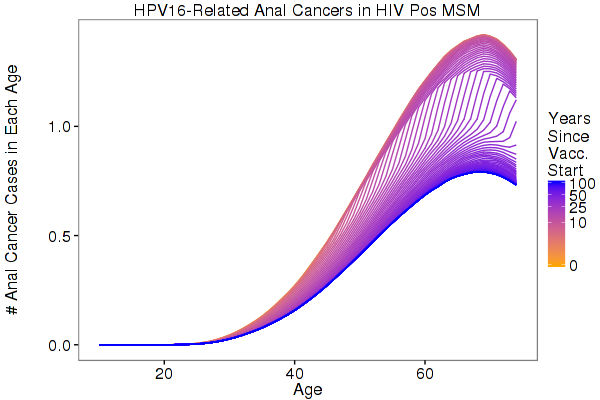


b) Vaccinating all 16-to-40-year-old MSM with lifelong vaccine-induced immunity


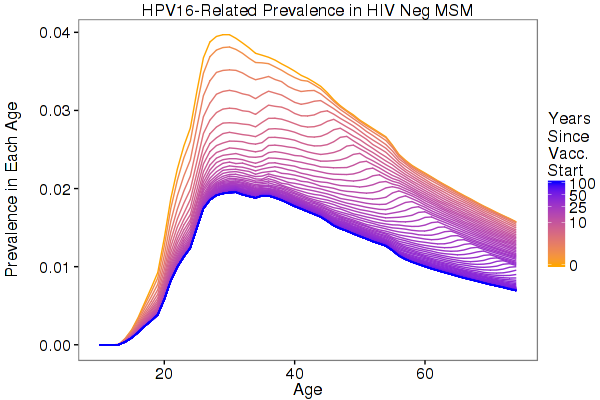

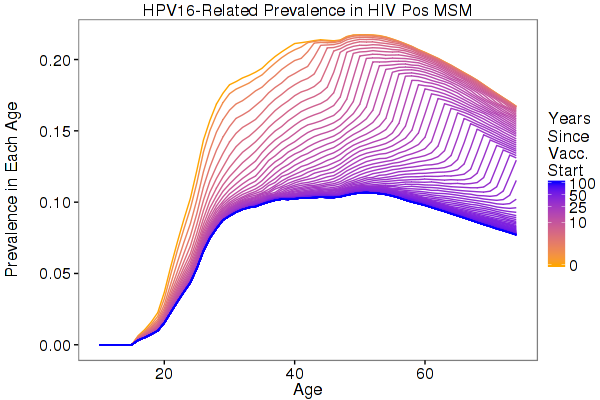

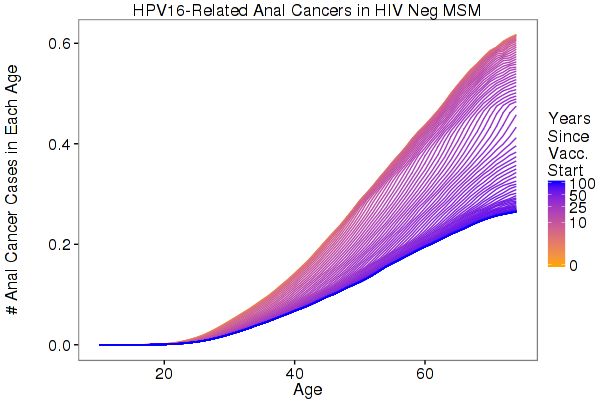

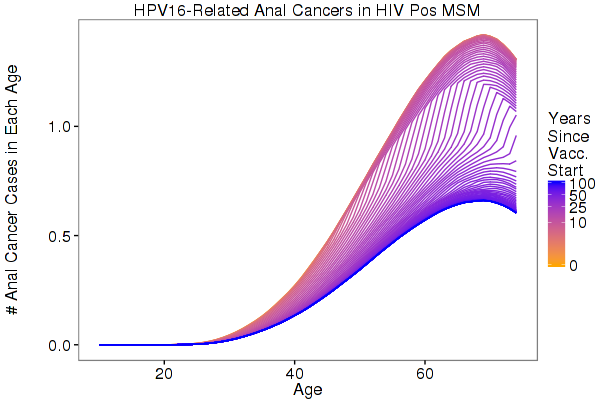


Figure. Effect of vaccination on HPV-16 prevalence and anal cancers over 100 years with lifelong vaccine-induced immunity.

a) Vaccinating only HIV+ MSM in specified age-bands


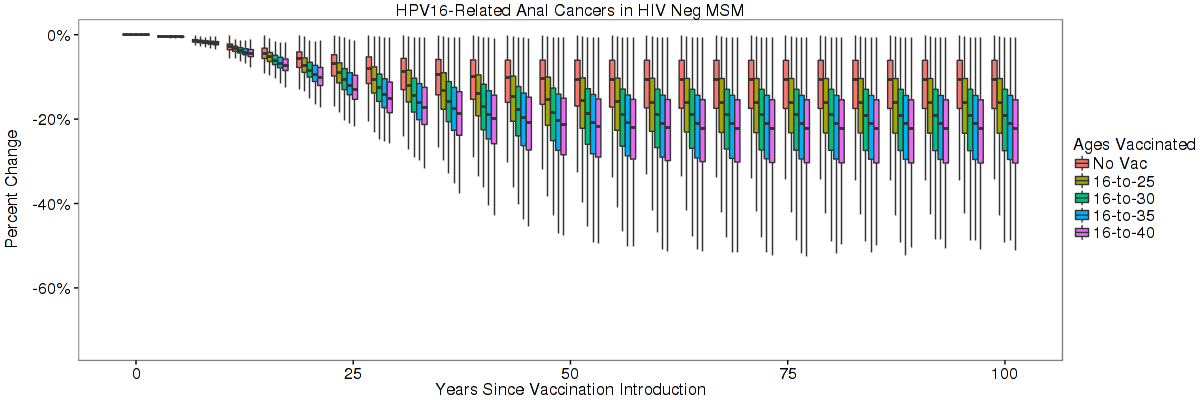


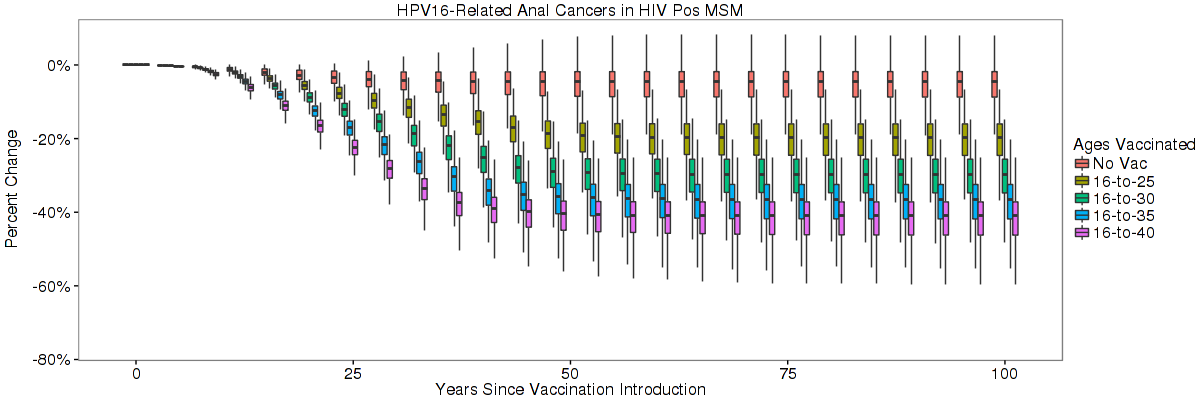


b) Vaccinating all MSM in specified age-bands


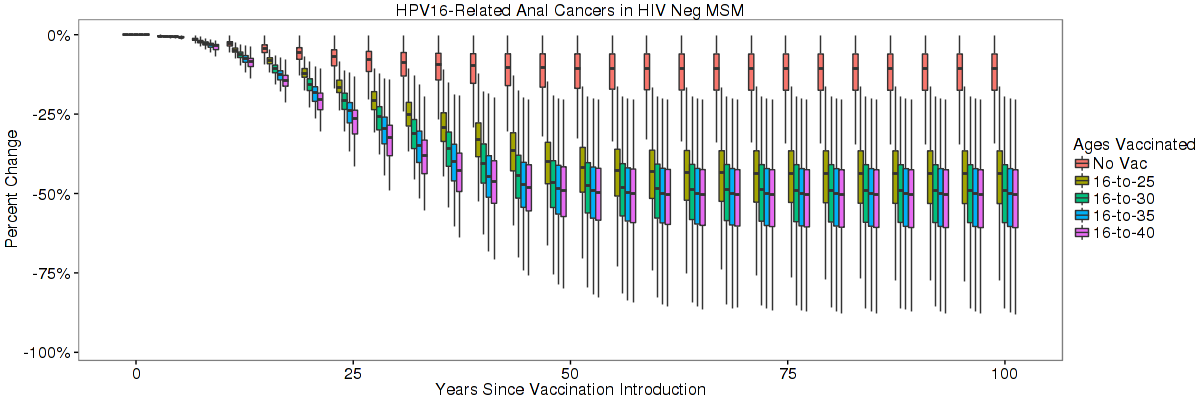


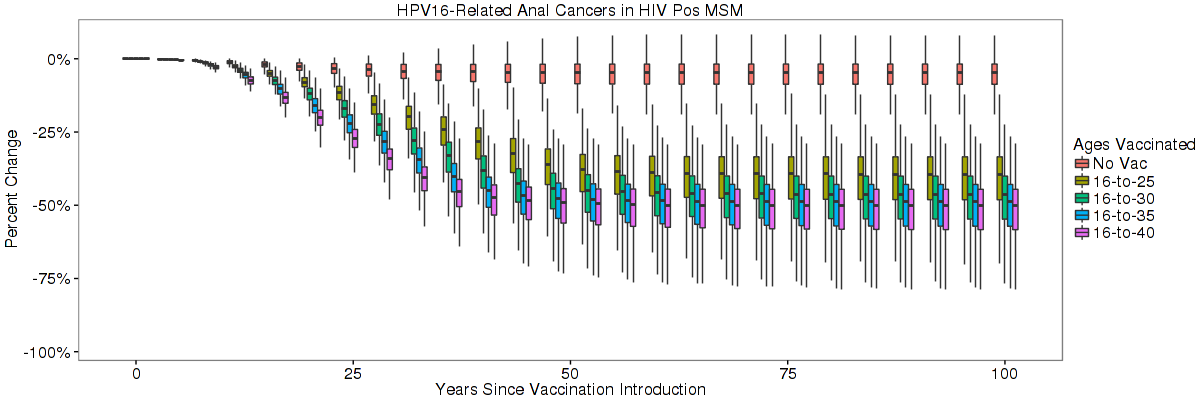


Figure. Effect of vaccination on HPV-16-related anal cancers over 100 years with lifelong vaccine-induced immunity.

a) Vaccinating HIV+ 16-to-40-year-old MSM with lifelong vaccine-induced immunity


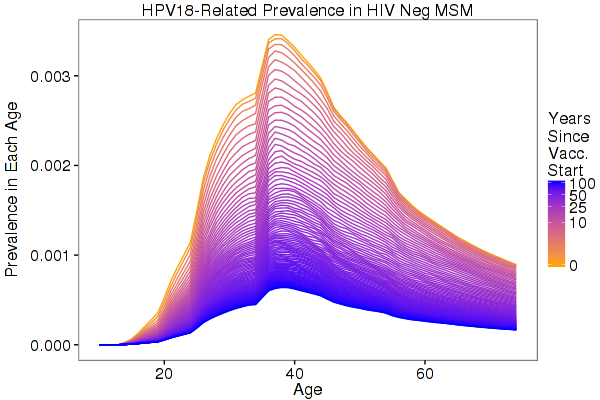

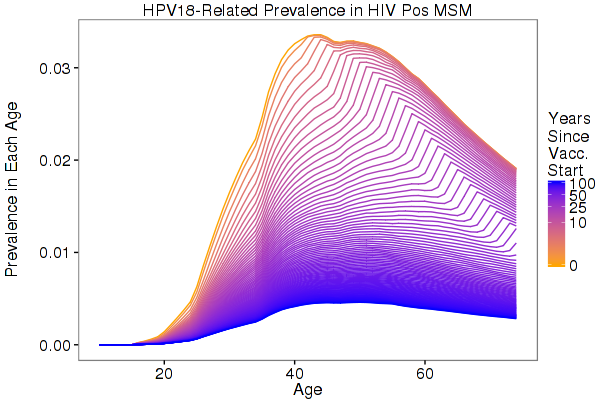


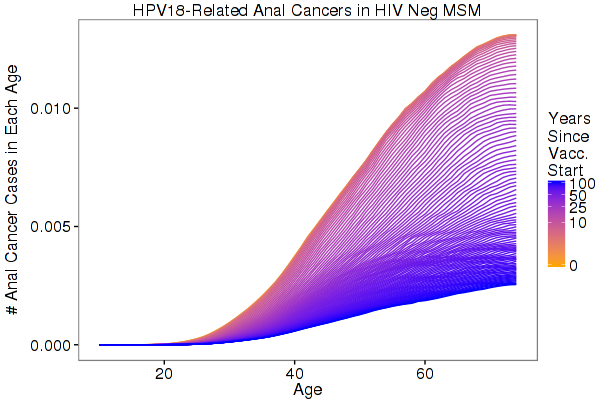

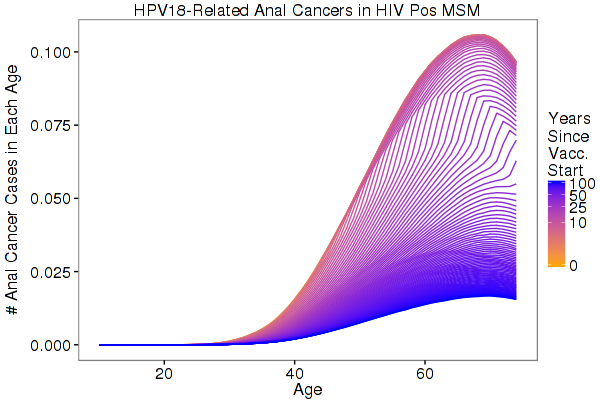


b) Vaccinating all 16-to-40-year-old MSM with lifelong vaccine-induced immunity

**
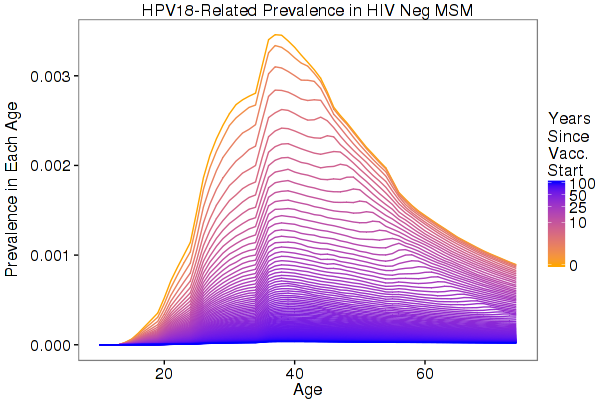

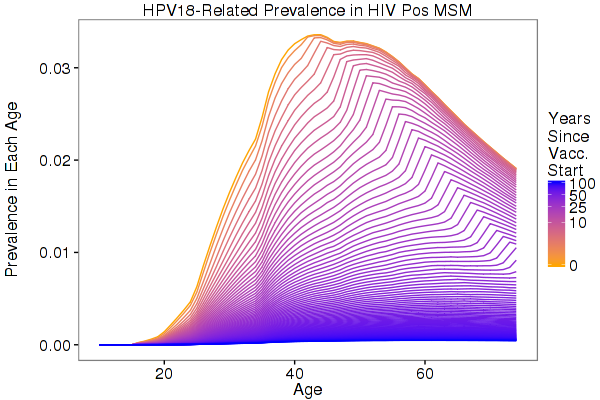
**


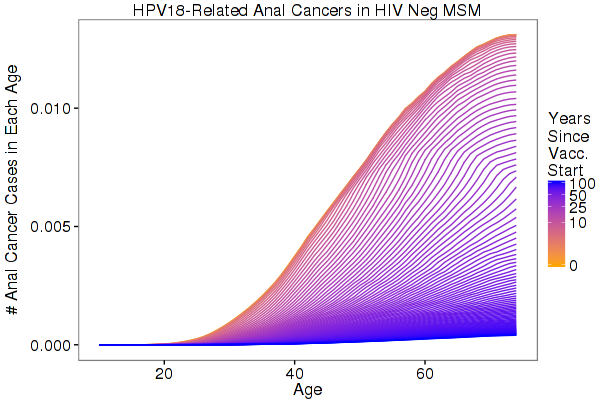

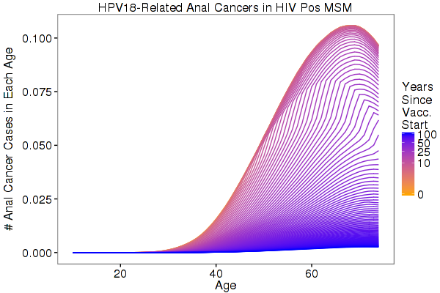


Figure. Effect of vaccination on HPV-18 prevalence and anal cancers over 100 years with lifelong vaccine-induced immunity.

a) Vaccinating only HIV+ MSM in specified age-bands


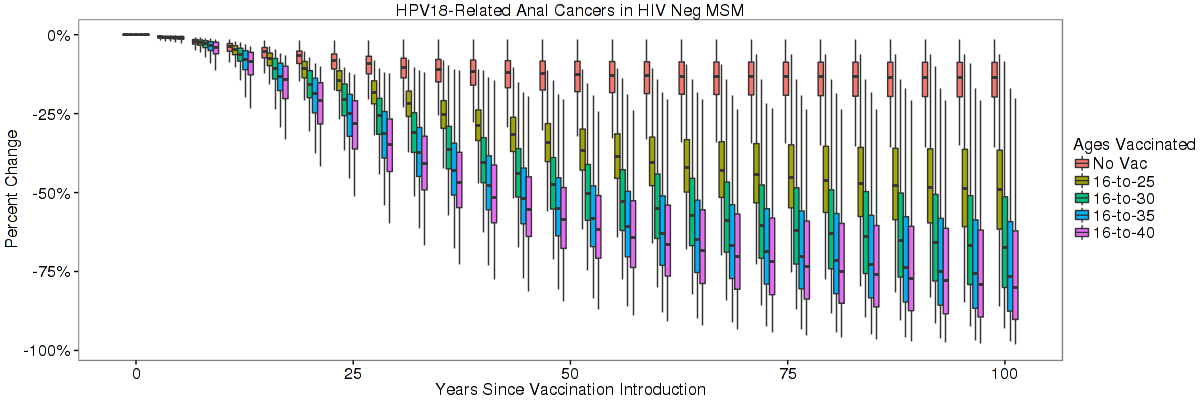


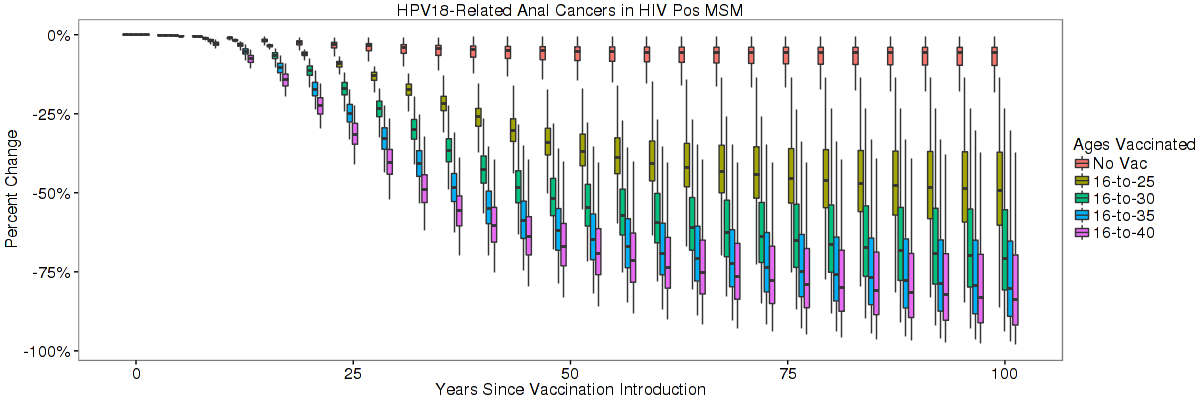


b) Vaccinating all MSM in specified age-bands


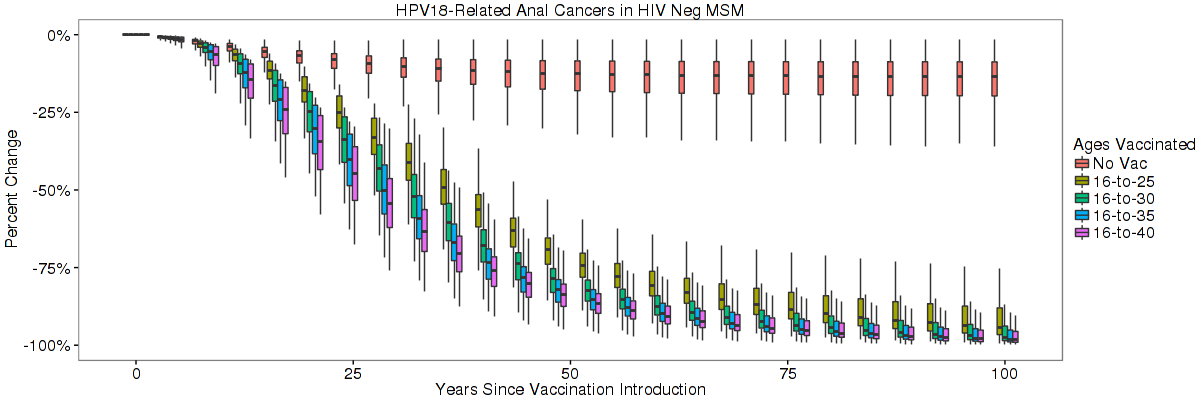


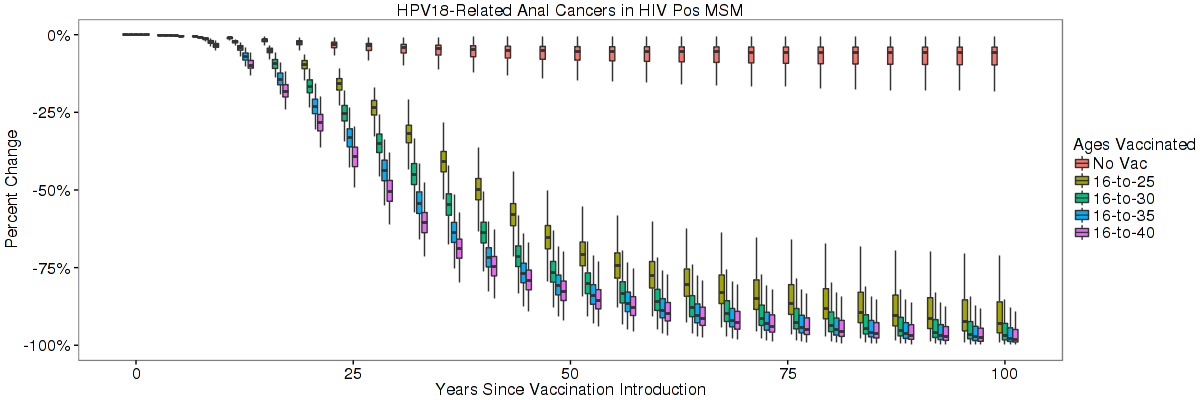


Figure. Effect of vaccination on HPV-18-related anal cancers over 100 years with lifelong vaccine-induced immunity.

# A11. Model outcomes summarized

**Figure.** Total change over 100 years in cases of anogenital warts and anal cancer (discounted at 3.5% a year) following quadrivalent HPV vaccination of MSM attending GUM clinics. Error bars show interquartile range across 1000 meta-scenarios.

# A12. One-way sensitivity analyses

In these sensitivity analyses, we vary economic parameters (except for vaccine cost) by their ranges in their table in appendix A5 for two comparisons (vaccinating 16-40 year old HIV-positive MSM vs 16-35 year old HIV-positive MSM, and vaccinating all 16-40 year old MSM versus 16-40 year old HIV-positive MSM) at two vaccine costs (£96.50 per dose or £48 per dose). In plots where the vaccine cost is £96.50, we show the cost per QALY gained when the vaccine cost is lowered to £48 per dose.

**Figure.** Vaccinating 16-40-year-old HIV-positive MSM versus 16-35-year-old HIV-positive MSM, in base case scenario with a vaccine cost of £96.50 per dose.

**Figure.** Vaccinating 16-40-year-old HIV-positive MSM versus 16-35-year-old HIV-positive MSM, in base case scenario with a vaccine cost of £48 per dose.

**Figure.** Vaccinating all 16-40 year old MSM versus 16-40 year old HIV-positive MSM, in base case scenario with a vaccine cost of £96.50 per dose.

**Figure.** Vaccinating all 16-40 year old MSM versus 16-40 year old HIV-positive MSM, in base case scenario with a vaccine cost of £48 per dose.

# A13. Cost-effectiveness acceptability curves

Vaccination programmes are considered to be cost-effective in England by JCVI if the most plausible ICER falls below £20,000 per QALY gained, and there is no more than a 10% probability that the ICER exceeds £30,000 per QALY gained [62].

At a vaccine cost of £96.50 a dose, vaccinating 16-40 year old HIV-positive MSM is more cost-effective than the next best alternative (vaccinating 16-35 year old HIV-positive MSM) in 99.7% of scenarios when the threshold is £20,000 per QALY gained, and in all scenarios when the threshold is £30,000 per QALY gained. Thus, at £96.50 per dose, vaccinating 16-40 year old HIV-positive MSM would satisfy these conditions. As a note, at £30,000 per QALY gained, vaccinating all 16-40 year old MSM is more cost-effective than the next best alternative (vaccinating 16-40 year old HIV-positive MSM) in 29.4% of scenarios.

At a lower vaccine cost of £48 a dose, vaccinating all 16-40 year old MSM is more cost-effective than the next best alternative (vaccinating 16-40 year old HIV-positive MSM) in 85.4% of scenarios when the threshold is £20,000 per QALY gained, and in 99.3% of scenarios when the threshold is £30,000 per QALY gained. Hence, at £48 per dose, vaccinating 16-40 year old MSM would satisfy these conditions.

In the figures below, we present the cost-effectiveness acceptability curves across 1,000 meta-scenarios for both £96.50 and £48 a dose. We also present the acceptability curves when only HIV-neutral vaccination is permissible.

**Figure**. Cost-effectiveness acceptability curves for £96.50 a vaccine dose.

**Figure**. Cost-effectiveness acceptability curves for £48 a vaccine dose.

**Figure**. Cost-effectiveness acceptability curves for £96.50 a vaccine dose for HIV-neutral vaccination scenarios only.

**Figure**. Cost-effectiveness acceptability curves for £48 a vaccine dose for HIV-neutral vaccination scenarios only.

# References

1. Mercer CH, Tanton C, Prah P, et al. Changes in sexual attitudes and lifestyles in Britain through the life course and over time: findings from the National Surveys of Sexual Attitudes and Lifestyles (Natsal). Lancet **2013**; 382:1781–94.

2. Erens B, Phelps A, Clifton S, et al. Methodology of the third British National Survey of Sexual Attitudes and Lifestyles (Natsal-3). Sex. Transm. Infect. **2014**; 90:84–9.

3. Mercer CH, Fenton KA, Copas AJ, et al. Increasing prevalence of male homosexual partnerships and practices in Britain 1990-2000: evidence from national probability surveys. AIDS **2004**; 18:1453–8.

4. Choi YH, Jit M, Gay N, Cox A, Garnett GP, Edmunds WJ. Transmission dynamic modelling of the impact of human papillomavirus vaccination in the United Kingdom. Vaccine **2010**; 28:4091–4102.

5. Office for National Statistics. Population Estimates for UK, England and Wales, Scotland and Northern Ireland, Mid-2011 and Mid-2012. Newport, United Kingdom: 2012.

6. Yin Z, Brown AE, Hughes G, Nardone A, Gill ON, Delpech VC. HIV in the United Kingdom 2014 Report: data to end 2013. 2014. Available at: https://www.gov.uk/government/uploads/system/uploads/attachment_data/file/401662/2014_PHE_HIV_annual_report_draft_Final_07-01-2015.pdf. Accessed 7 September 2016.

7. Erens B, Mcmanus S, Prescott A, et al. National Survey of Sexual Attitudes and Lifestyles II Reference tables and summary report with National Survey of Sexual Attitudes and Lifestyles II.

8. Aghaizu A, Wayal S, Nardone A, et al. Sexual behaviours, HIV testing, and the proportion of men at risk of transmitting and acquiring HIV in London, UK, 2000-13: a serial cross-sectional study. lancet. HIV **2016**; 3:e431-40.

9. Mesher D, Panwar K, Thomas SL, Beddows S, Soldan K. Continuing reductions in HPV 16/18 in a population with high coverage of bivalent HPV vaccination in England: an ongoing cross-sectional study. BMJ Open **2016**; 6:e009915.

10. Savage EJ, Mohammed H, Leong G, Duffell S, Hughes G. Improving surveillance of sexually transmitted infections using mandatory electronic clinical reporting: the genitourinary medicine clinic activity dataset, England, 2009 to 2013. Euro Surveill. Bull. Eur. sur les Mal. Transm. = Eur. Commun. Dis. Bull. **2014**; 19:20981.

11. Brown AE, Gill ON, Delpech VC. HIV treatment as prevention among men who have sex with men in the UK: is transmission controlled by universal access to HIV treatment and care? HIV Med. **2013**; 14:563–70.

12. Milne C, Bayley J, Taylor C. A retrospective case note analysis of hepatitis B vaccination prescribing in a genito-urinary clinic setting: what is the role of the fourth vaccine? HIV Med. **2014**; 15:154.

13. King EM, Gilson R, Beddows S, et al. Human papillomavirus DNA in men who have sex with men: type-specific prevalence, risk factors and implications for vaccination strategies. Br. J. Cancer **2015**; 112:1585–93.

14. British HIV Association. Routine investigation and monitoring of adult HIV-1-infected individuals (consultation draft version). 2016. Available at: http://www.bhiva.org/documents/Guidelines/Monitoring/160606-Monitoring-gl-draft-for-Consultation.pdf. Accessed 6 September 2016.

15. Fakoya A, Lamba H, Mackie N, et al. British HIV Association, BASHH and FSRH guidelines for the management of the sexual and reproductive health of people living with HIV infection 2008. HIV Med. **2008**; 9:681–720.

16. Desai S, Wetten S, Woodhall SC, Peters L, Hughes G, Soldan K. Genital warts and cost of care in England. Sex. Transm. Infect. **2011**; 87:464–8.

17. de Angelis D, Presanis A, Conti S, Ades A. Estimation of HIV burden through Bayesian evidence synthesis. Stat. Sci. **2014**; 29:9–17.

18. Public Health England. HIV surveillance systems. 2008. Available at: https://www.gov.uk/hiv-surveillance-systems. Accessed 16 February 2016.

19. Aghaizu A, Brown AE, Nardone A, Gill ON, Delpech VC. HIV in the United Kingdom 2013 Report: data to end 2012. London, United Kingdom: 2013. Available at: https://www.gov.uk/government/uploads/system/uploads/attachment_data/file/326601/HIV_annual_report_2013.pdf. Accessed 6 September 2016.

20. Hickson F, Bourne A, Weatherburn P, Reid D, Jessup K, Hammond G. Tactical dangers: Findings from the United Kingdom Gay Men’s Sex Survey 2008. 2010. Available at: http://sigmaresearch.org.uk/files/report2010b.pdf. Accessed 7 September 2016.

21. Giuliano AR, Palefsky JM, Goldstone S, et al. Efficacy of quadrivalent HPV vaccine against HPV infection and disease in males. Lancet **2011**; 364:401–411.

22. Kojic EM, Kang M, Cespedes MS, et al. Immunogenicity and Safety of the Quadrivalent Human Papillomavirus Vaccine in HIV-1-Infected Women. Clin. Infect. Dis. **2014**; 59:127–135.

23. De Vuyst H, Clifford GM, Nascimento MC, Madeleine MM, Franceschi S. Prevalence and type distribution of human papillomavirus in carcinoma and intraepithelial neoplasia of the vulva, vagina and anus: a meta-analysis. Int. J. Cancer **2009**; 124:1626–36.

24. Machalek DA, Poynten M, Jin F, et al. Anal human papillomavirus infection and associated neoplastic lesions in men who have sex with men: a systematic review and meta-analysis. Lancet Oncol. **2012**; 13:487–500.

25. Nyitray AG, Carvalho da Silva RJ, Baggio ML, et al. Age-specific prevalence of and risk factors for anal human papillomavirus (HPV) among men who have sex with women and men who have sex with men: the HPV in men (HIM) study. J. Infect. Dis. **2011**; 203:49–57.

26. Kreimer AR, Pierce Campbell CM, Lin H-Y, et al. Incidence and clearance of oral human papillomavirus infection in men: the HIM cohort study. Lancet **2013**; 382:877–87.

27. Grulich AE, van Leeuwen MT, Falster MO, Vajdic CM. Incidence of cancers in people with HIV/AIDS compared with immunosuppressed transplant recipients: a meta-analysis. Lancet **2007**; 370:59–67.

28. Miralles-Guri C, Bruni L, Cubilla a L, Castellsagué X, Bosch FX, de Sanjosé S. Human papillomavirus prevalence and type distribution in penile carcinoma. J. Clin. Pathol. **2009**; 62:870–8.

29. Kreimer AR, Clifford GM, Boyle P, Franceschi S. Human papillomavirus types in head and neck squamous cell carcinomas worldwide: a systematic review. Cancer Epidemiol.Biomarkers Prev. **2005**; 14:467–475.

30. Evans M, Newcombe R, Fiander A, et al. Human Papillomavirus-associated oropharyngeal cancer: An observational study of diagnosis, prevalence and prognosis in a UK population. BMC Cancer **2013**; 13.

31. de Pokomandy A, Rouleau D, Ghattas G, et al. Prevalence, clearance, and incidence of anal human papillomavirus infection in HIV-infected men: the HIPVIRG cohort study. J. Infect. Dis. **2009**; 199:965–73.

32. Giuliano A, Lee J, Fulp W, Villa L. Incidence and clearance of genital human papillomavirus infection in men (HIM): a cohort study. Lancet **2011**; 377:932–940.

33. Gravitt PE. The known unknowns of HPV natural history. J. Clin. Invest. **2011**; 121:4593–9.

34. Alemany L, Saunier M, Alvarado-Cabrero I, et al. Human papillomavirus DNA prevalence and type distribution in anal carcinomas worldwide. Int. J. Cancer **2015**; 136:98–107.

35. Hawkins MG, Winder DM, Ball SLR, et al. Detection of specific HPV subtypes responsible for the pathogenesis of condylomata acuminata. Virol. J. **2013**; 10:1.

36. Ball SLR, Winder DM, Vaughan K, et al. Analyses of Human Papillomavirus Genotypes and Viral Loads in Anogenital Warts. **2011**; 1350:1345–1350.

37. Brown DR, Schroeder JM, Bryan JT, Stoler MH, Fife KH. Detection of multiple human papillomavirus types in Condylomata acuminata lesions from otherwise healthy and immunosuppressed patients. J Clin Microbiol **1999**; 37:3316–3322.

38. Palefsky JM, Giuliano AR, Goldstone S, et al. HPV vaccine against anal HPV infection and anal intraepithelial neoplasia. N. Engl. J. Med. **2011**; 365:1576–85.

39. Palefsky JM, Holly E, Hogeboom C, Ralston M, Da Costa M, Botts R. Virologic, Immunologic, and Clinical Parameters in the Incidence and Progression of Anal Squamous Intraepithelial Lesions in HIV-Positive and HIV-Negative Homosexual Men. JAIDS J. Acquir. Immune Defic. Syndr. **1998**; 17:314–9.

40. Woodhall SC, Jit M, Soldan K, et al. The impact of genital warts: loss of quality of life and cost of treatment in eight sexual health clinics in the UK. Sex. Transm. Infect. **2011**; 87:458–63.

41. Jit M, Chapman R, Hughes O, Choi YH. Comparing bivalent and quadrivalent human papillomavirus vaccines: economic evaluation based on transmission model. BMJ **2011**; 343:d5775.

42. Rogers SN, Miller RD, Ali K, Minhas AB, Williams HF, Lowe D. Patients’ perceived health status following primary surgery for oral and oropharyngeal cancer. Int.J.Oral Maxillofac.Surg. **2006**; 35:913–919.

43. Office for National Statistics. ONS One- and five-year survival of patients diagnosed in 1991-95 and 1996-99: less common cancers, sex and age, England and Wales. 2005. Available at: https://www.ons.gov.uk/file?uri=/peoplepopulationandcommunity/healthandsocialcare/conditionsanddiseases/datasets/cancersurvivalratescancersurvivalenglandandwaleslesscommoncancersbyagegroup/19911999/lesscommoncancersbyagegroup_tcm77-252734.xls. Accessed 6 September 2016.

44. Office for National Statistics. ONS: Cancer survival rates, Cancer Survival in England: Patients Diagnosed 2007-2011 and Followed up to 2012. 2013. Available at: http://www.ons.gov.uk/ons/rel/cancer-unit/cancer-survival/cancer-survival-in-england--patients-diagnosed-2007-2011-and-followed-up-to-2012/stb-cancer-survival-in-england--patients-diagnosed-2007-2011-and-followed-up-to-2012.html. Accessed 1 December 2015.

45. Ragin CCR, Taioli E. Survival of squamous cell carcinoma of the head and neck in relation to human papillomavirus infection: review and meta-analysis. Int. J. Cancer **2007**; 121:1813–20.

46. Arya M, Li R, Pegler K, et al. Long-term trends in incidence, survival and mortality of primary penile cancer in England. Cancer Causes Control **2013**; 24:2169–76.

47. Jeffreys M, Rachet B, McDowell S, Habib AG, Lepage C, Coleman MP. Survival from rectal and anal cancers in England and Wales, 1986-2001. Eur. J. Cancer **2006**; 42:1434–40.

48. James RD, Glynne-Jones R, Meadows HM, et al. Mitomycin or cisplatin chemoradiation with or without maintenance chemotherapy for treatment of squamous-cell carcinoma of the anus (ACT II): a randomised, phase 3, open-label, 2 × 2 factorial trial. Lancet. Oncol. **2013**; 14:516–24.

49. Fakhry C, Zhang Q, Nguyen-Tan PF, et al. Human Papillomavirus and Overall Survival After Progression of Oropharyngeal Squamous Cell Carcinoma. J. Clin. Oncol. **2014**; :1–14.

50. Lassen P. The role of Human papillomavirus in head and neck cancer and the impact on radiotherapy outcome. Radiother. Oncol. **2010**; 95:371–80.

51. Ang KK, Harris J, Wheeler R, et al. Human papillomavirus and survival of patients with oropharyngeal cancer. N. Engl. J. Med. **2010**; 363:24–35.

52. Jick H, Hagberg KW. Effectiveness of influenza vaccination in the United kingdom, 1996-2007. Pharmacotherapy **2010**; 30:1199–1206.

53. Institute of Medicine Committee to Study Priorities for Vaccine Development. Vaccines for the 21st Century: A Tool for Decisionmaking. 2003. Washington, DC: Institute of Medicine. Washington, D.C.: National Academies Press (US), 2000.

54. Bleeker MCG, Heideman DAM, Snijders PJF, Horenblas S, Dillner J, Meijer CJLM. Penile cancer: epidemiology, pathogenesis and prevention. World J. Urol. **2009**; 27:141–150.

55. Préaud E, Largeron N. Economic burden of non-cervical cancers attributable to human papillomavirus: a European scoping review. J. Med. Econ. **2013**; 16:763–76.

56. Keeping ST, Tempest MJ, Stephens SJ, Carroll SM, Sangar VK. Penile cancer treatment costs in England. BMC Public Health **2015**; 15:1305.

57. Palefsky JM, Holly EA, Ralston ML, et al. Anal squamous intraepithelial lesions in HIV-positive and HIV-negative homosexual and bisexual men: prevalence and risk factors. J. Acquir. Immune Defic. Syndr. Hum. Retrovirol. **1998**; 17:320–6.

58. de Pokomandy A, Rouleau D, Ghattas G, et al. HAART and progression to high-grade anal intraepithelial neoplasia in men who have sex with men and are infected with HIV. Clin. Infect. Dis. **2011**; 52:1174–81.

59. Curtis L. Unit Costs of Health & Social Care 2014. Kent, United Kingdom: 2014.

60. British Medical Association, Royal Pharmaceutical Society. British National Formulary - August 2015. London, United Kingdom: British National Formulary Publications, 2015.

61. Conway EL, Farmer KC, Lynch WJ, Rees GL, Wain G, Adams J. Quality of life valuations of HPV-associated cancer health states by the general population. Sex. Transm. Infect. **2012**; 88:517–21.

62. Joint Committee on Vaccination and Immunisation. Joint Committee on Vaccination and Immunisation - Code of Practice June 2013. 2013. Available at: https://www.gov.uk/government/uploads/system/uploads/attachment_data/file/224864/JCVI_Code_of_Practice_revision_2013_-_final.pdf. Accessed 15 January 2016.
